# An extensive experimental analysis for heart disease prediction using artificial intelligence techniques

**DOI:** 10.1038/s41598-025-90530-1

**Published:** 2025-02-20

**Authors:** D. Rohan, G. Pradeep Reddy, Y. V. Pavan Kumar, K. Purna Prakash, Ch. Pradeep Reddy

**Affiliations:** 1 School of Computer Science and Engineering, VIT-AP University, Amaravati, 522241 Andhra Pradesh India; 2 Department of Information and Communication Technology, Manipal Institute of Technology, Manipal Academy of Higher Education, Manipal, 576104 Karnataka India; 3 School of Electronics Engineering, VIT-AP University, Amaravati, 522241 Andhra Pradesh India; 4 Department of Computer Science and Engineering, Koneru Lakshmaiah Education Foundation, Vaddeswaram, 522502 Andhra Pradesh India; 5 Department of Computer Science, Chandigarh Engineering College, Chandigarh Group of Colleges Jhanjeri, Mohali, 140307 Punjab India

**Keywords:** Heart disease prediction, Artificial intelligence, Feature selection, Machine learning, Deep learning, XGBoost, Performance metrics, Heart failure, Biomedical engineering

## Abstract

The heart is an important organ that plays a crucial role in maintaining life. Unfortunately, heart disease is one of the major causes of mortality globally. Early and accurate detection can significantly improve the situation by enabling preventive measures and personalized healthcare recommendations. Artificial intelligence is emerging as a powerful tool for healthcare applications, particularly in predicting heart diseases. Researchers are actively working on this, but challenges remain in achieving accurate heart disease prediction. Therefore, experimenting with various models to identify the most effective one for heart disease prediction is crucial. In this view, this paper addresses this need by conducting an extensive investigation of various models. The proposed research considered 11 feature selection techniques and 21 classifiers for the experiment. The feature selection techniques considered for the research are Information Gain, Chi-Square Test, Fisher Discriminant Analysis (FDA), Variance Threshold, Mean Absolute Difference (MAD), Dispersion Ratio, Relief, LASSO, Random Forest Importance, Linear Discriminant Analysis (LDA), and Principal Component Analysis (PCA). The classifiers considered for the research are Logistic Regression, Decision Tree, Random Forest, K-Nearest Neighbors (KNN), Support Vector Machine (SVM), Gaussian Naïve Bayes (GNB), XGBoost, AdaBoost, Stochastic Gradient Descent (SGD), Gradient Boosting Classifier, Extra Tree Classifier, CatBoost, LightGBM, Multilayer Perceptron (MLP), Recurrent Neural Network (RNN), Long Short-Term Memory (LSTM), Gated Recurrent Unit (GRU), Bidirectional LSTM (BiLSTM), Bidirectional GRU (BiGRU), Convolutional Neural Network (CNN), and Hybrid Model (CNN, RNN, LSTM, GRU, BiLSTM, BiGRU). Among all the extensive experiments, XGBoost outperformed all others, achieving an accuracy of 0.97, precision of 0.97, sensitivity of 0.98, specificity of 0.98, F1 score of 0.98, and AUC of 0.98.

## Introduction

As per the recent World Heart Report, 20.5 million deaths were accounted to cardiovascular disease (CVD) globally^1^. Heart disease can be caused due to reduced flow of blood to the heart, infection, atherosclerosis, high blood pressure, or uncontrolled diabetes. The common types are heart failure, heart attack, myocarditis, sudden cardiac arrest, atrial septal defect, atrial fibrillation, coronary heart disease, angina, ventricular tachycardia, and pericarditis.Heart failure impacts the hearts’ ability to pump blood effectively. Heart attack is due to the block in arteries which causes loss of blood supply. Inflammation of the myocardium is myocarditis, which is usually caused by a viral infection or bacterial infection or can be due to a fungal infection. Sudden cardiac arrest is different from a heart attack. As the name says, it is a condition where the heart suddenly stops beating. The atrial septal defect occurs due to the presence of a hole in the wall in between atria. An irregular heartbeat leads to atrial fibrillation. A condition where the major blood vessels or coronary arteries are narrowed leads to coronary heart disease. Receiving insufficient oxygen-rich blood may lead to chest discomfort, often referred to as angina, a type of chest pain. The fast heartbeat rhythm of the lower chambers of the heart or the ventricles causes ventricular tachycardia. Pericarditis is caused by the inflammation of a thin membrane around the heart called pericardium. Early detection of heart disease is important to prevent adverse outcomes and reduce the burden on healthcare systems.

The term “Artificial Intelligence” was coined at the Dartmouth conference in 1956. In the 1950s, Alan Turing proposed the Turing Test, a benchmark for machine intelligence. Knowledge-based systems emerged in the 1970s, with MYCIN (designed to identify infection-causing bacteria) as a notable example. Artificial intelligence gained further prominence in the 1990s with the development of neural networks and backpropagation. Advances in computing power in the 2000s led to the development of techniques such as natural language processing (NLP) and image recognition. From the 2010s, deep learning has become a dominant force in AI development. Machine learning algorithms, particularly supervised learning, unsupervised learning, and reinforcement learning have shown great promise in healthcare. Deep learning techniques such as neural networks, multilayer perceptron (MLP), convolutional neural networks (CNNs), and recurrent neural networks (RNNs) are also being actively used. Long Short-Term Memory (LSTM) and Gated Recurrent Unit (GRU) are the variants of RNN. RNNs and their variants have a wide range of applications beyond healthcare, including natural language processing, time series prediction, video analysis, music generation, and robotics. Deep learning has recently demonstrated significant potential in detecting COVID-19^2^. Explainable AI (XAI) plays a major role in healthcare systems by addressing the transparency and interpretability of the models^3^. It comes into play when the decisions made by these models may lead to high consequences. It helps in clinical decision support, treatment recommendation, ensuring fairness, and avoiding bias. Federated learning is also widely used in healthcare systems^4^. Various research works that are related to the objective of this paper are segregated in terms of literature related to Artificial Intelligence, the Internet of Things (IoT) and a combination of these are discussed as follows. With the current advancements in neural network architectures and computing power, researchers used models such as artificial neural network (ANN) and deep neural network (DNN) for heart disease classification tasks^5,6^. The integration of the IoT with deep learning-modified neural networks (DLMNN) was designed to predict the presence of heart disease. This involves three phases - authentication, encryption, and classification. The DLMNN was trained on the Hungarian heart disease dataset. With 100 nodes, the DLMNN achieved 92% accuracy, a 92.59% F1 score, with 500 nodes it achieved 96.8% accuracy, a 98.25% F1 score. The IoT-centered DLMNN also achieved 95.82% security during the data transfer and exhibited the lowest encryption and decryption time^7^. Similarly, IoT-based heart failure prediction complex event processing for heart failure prediction (CEP4HFP) was presented^8^. This consists of three modules namely monitoring, analysis, and visualization. Arduino MEGA microcontroller and Raspberry Pi were used for monitoring, and NoSQL and CEP engines were used for data storage and analysis respectively. CEP4HFP achieved 84.75% precision and 91.74% F1 score. For heart disease-related works, the Cleveland dataset is widely used by researchers^9–11^. A hybrid random forest with a linear model was developed and implemented on the Cleveland dataset to predict heart disease^12,13^. Similarly, another work based on random forest, namely the machine intelligence framework for heart disease diagnosis (MIFH) was proposed in^14^. MIFH is a random forest classifier with a factor analysis of mixed data (FAMD). FAMD was used on the Cleveland dataset as a feature selection algorithm with classifiers such as logistic regression (LR), K-Nearest Neighbors (KNN), Support Vector Machine (SVM), decision tree (DT), and random forest (RF). Data mining is a vital step in machine learning, especially for classification tasks to discover important patterns, insights, and relationships from the raw data is essential in building effective machine learning models. Various classifiers were implemented after applying data mining techniques on the Cleveland dataset^15,16^. Boosting is an ensemble learning technique that combines the predictions of multiple weak learners to create a strong learner^17^. XGBoost is a well-known member of its family and a widely used boosting technique. Various research works that used boosting were discussed^18–21^. Several methods such as decision tree classifier, K-means clustering, and SVM were also considered for heart disease-related research^22–25^. Further, to improve the performance of various models, numerous feature selection and oversampling techniques were used^26,27^. Apart from the Cleveland dataset, researchers also considered other datasets such as the Z-Alizadeh Sani dataset, and the CHD dataset for the experiments^28,29^. Recent advancements in hybrid optimization and rule-mining techniques have also contributed to the prediction of heart diseases. One such approach is the Grey Wolf Levy Updated-Dragonfly Algorithm (GWU-DA), which integrates Grey Wolf Optimization (GWO) with the Dragonfly Algorithm (DA) for optimized feature selection^30^. This model leverages weighted coalesce rule generation and hybrid classifiers, combining SVM with Deep Belief Networks (DBN), to effectively predict the presence of heart disease and other conditions such as breast cancer. Furthermore, two-phase parallel frameworks employing weighted coalesce rule mining have been developed to accelerate disease prediction tasks, efficiently handling large datasets^31,32^. Although there were several works on heart disease prediction, attaining precise heart disease prediction is still a challenge. In healthcare, making a model with high performance is highly important. To address this challenge, this paper proposes an extensive experimental analysis for heart disease prediction using artificial intelligence techniques to identify the best model for heart disease prediction. The contributions of this work are given as follows.

- Comprehensive experimentation: 11 feature selection techniques and 21 classifiers were implemented on the heart disease dataset (Comprehensive)^33^.
- Two-phase methodology: The whole experiment was performed in two phases.
  - In the first phase, classifiers were directly implemented on the dataset.
  - In the second phase, the feature selection techniques are employed and later the classifiers are implemented.
- Optimized model performance: The XGBoost model has achieved 97.3% accuracy without feature selection and after hyperparameter tuning. This model’s performance is the highest among the other classifiers.

The rest of the paper is organized into the following sections: “Methodology”, results and discussion, and conclusion. Section 2 describes the methodology, Section 3 presents the results of feature selection techniques and classifiers, and Section 4 summarizes the “Results”.

## Methodology

**Fig. 1:**
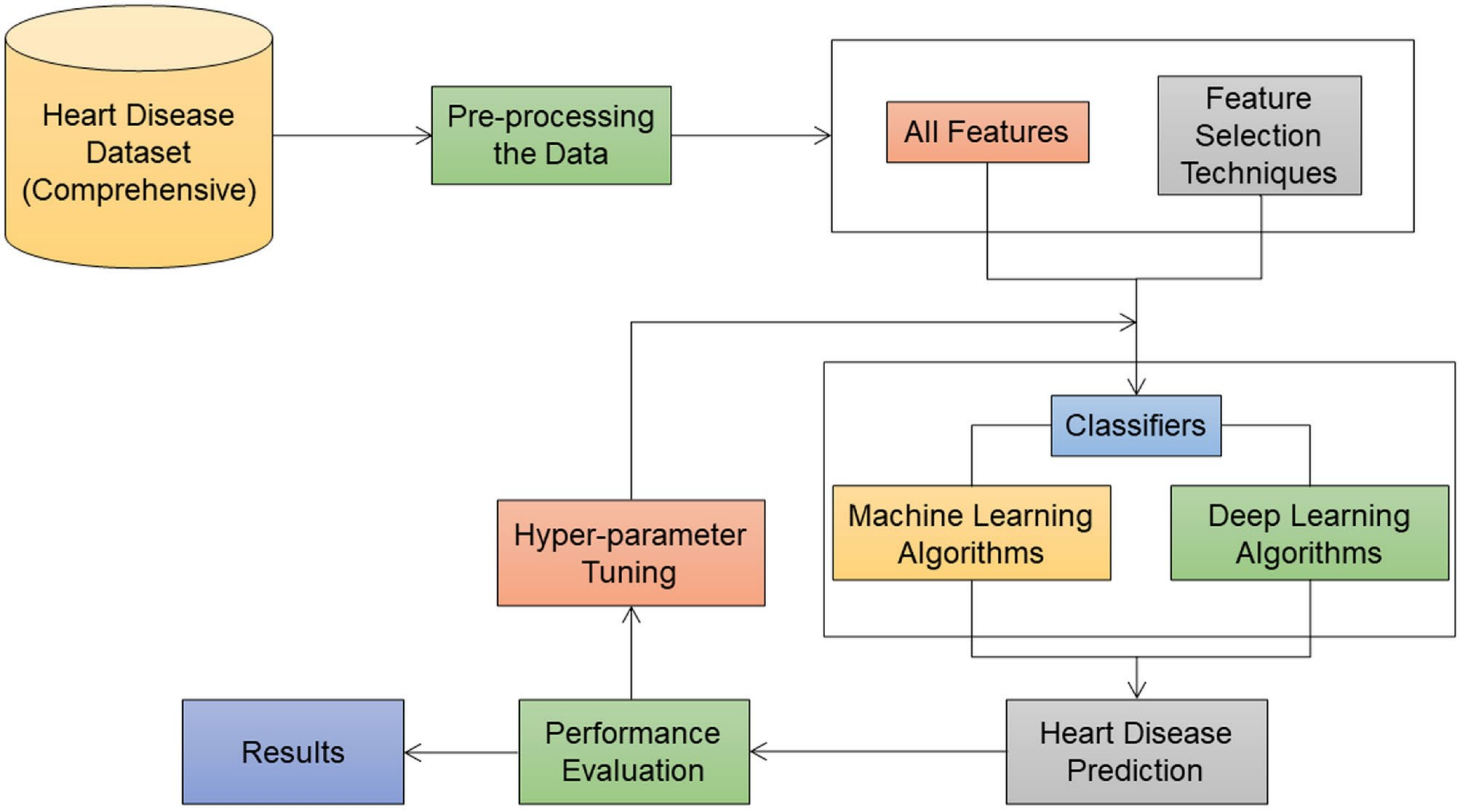
Execution flow of the experiment.

The proposed research is performed in two approaches as shown in Fig. 1. In the first approach, 21 classifiers are directly applied to the dataset to predict heart disease and then the performance evaluation is done. Further, hyperparameter tuning is performed on the classifiers. In the second approach, 11 feature selection techniques are applied to the dataset and 21 classifiers are applied to the features selected. The heart disease dataset (Comprehensive) is considered for conducting the proposed research^33^. This dataset consists of 11 features and 1190 instances. This dataset is curated by combining the 5 datasets with over 11 common features. The datasets used for the curation are Cleveland, Hungarian, Switzerland, Long Beach VA, and Statlog heart disease datasets. The 11 features of the dataset are tabulated in Table 1.

### Performance measures

Six metrics were considered for evaluating the classifiers: accuracy, precision, sensitivity, specificity, F1 score, and AUC. Accuracy measures overall correctness, i.e., the ratio of correctly predicted instances to the total number of instances in the dataset. Precision is used to find out the number of actual positives out of predicted positives. Sensitivity gives the proportion of actual positive cases correctly identified by the model, i.e., the true positive rate. Specificity gives the proportion of actual negative cases the model identifies, i.e., the true negative rate. F1 score is the harmonic mean of precision and sensitivity, which balances the false positives and false negatives. AUC measures the ability of the model to distinguish between the classes, which plots the true positive rate against the false positive rate.

### Statistical analysis

The dataset consists of both continuous and categorical features. The baseline characteristics were analyzed to compare two groups: patients with (group-1) and without (group-2) heart disease. For continuous features such as age, resting bp s, cholesterol, max heart rate, and old peak, mean ± standard deviation (SD) was calculated for both groups, and the p-values were calculated using the t-test. For categorical features such as sex, chest pain type, fasting blood sugar, resting ecg, exercise angina, and ST slope, the counts were calculated within two groups, and p-values were calculated using the chi-square test. The baseline characteristics p-values were calculated to determine if there was a statistically significant difference in a feature between the two groups. If the p-values of the features are less than 0.05, they are statistically significant. The p-values of both continuous and categorical features tabulated in Tables 2 and 3 are less than 0.05, indicating that they are statistically significant.

**Table 1:** Features of the dataset.

S. No.	Feature name	Feature description
1	Age	Age (in years)
2	Sex	Sex (1 = Male, 0 = Female)
3	Chest pain type	Type of chest pain (1 = typical angina, 2 = atypical angina, 3 = non-anginal pain, 4 = symptomatic)
4	Resting bp s	Resting BP (in mm Hg)
5	Cholesterol	Serum cholesterol (in mg/dl)
6	Fasting blood sugar	Fasting blood sugar > 120 mg/dl (1 = Yes, 0 = No)
7	Resting ecg	Resting electrocardiographic results (0 = Normal, 1 = ST-T wave abnormality, 2 = Presence of left ventricular hypertrophy)
8	Max heart rate	Maximum heart rate achieved
9	Exercise angina	Exercise-induced angina (1 = Yes, 0 = No)
10	Old peak	ST depression induced by exercise relative to rest
11	ST slope	The slope of the peak exercise ST segment (1 = Inclined upward, 2 = Flat, 3 = Inclined downward)

**Table 2:** Significance of continuous features.

S. No.	Feature	Group-1 (Mean ± SD)	Group-2 (Mean ± SD)	P-value	Significant
1	Age	56.03 ± 8.61	51.12 ± 9.49	6.61$\times 10^{-20}$	Yes
2	Resting bp s	134.26 ± 19.67	129.79 ± 16.49	2.22$\times 10^{-05}$	Yes
3	Cholesterol	191.37 ± 119.73	231.66 ± 70.02	1.39$\times 10^{-12}$	Yes
4	Max heart rate	129.78 ± 23.72	150.89 ± 22.70	1.70$\times 10^{-50}$	Yes
5	Oldpeak	1.33 ± 1.18	0.46 ± 0.73	3.18$\times 10^{-48}$	Yes

**Table 3:** Significance of categorical features.

S. No.	Feature	Group-1 (Counts)	Group-2 (Counts)	P-value	Significant
1	Age	1:559, 0:70	1: 350, 0: 211	1.42$\times 10^{-26}$	Yes
2	Chest pain type	4:483, 3:90, 2:31, 1:25	3:193, 2:185, 4:142, 1:41	3.53$\times 10^{-72}$	Yes
3	Fasting blood sugar	0:442, 1:187	0:494, 1:67	1.32$\times 10^{-13}$	Yes
4	Resting ecg	0:331, 2:179, 1:119	0:353, 2:146, 1:62	1.13$\times 10^{-04}$	Yes
5	Exercise angina	1:383, 0:246	0:483, 1:78	1.62$\times 10^{-61}$	Yes
6	ST slope	2:459, 1:110, 3:59, 0:1	1:416, 2:123, 3:22	1.26$\times 10^{-83}$	Yes

### Implementation environment

The experiment was carried out using various software and hardware resources. Python 3.10.14 was used to carry out the implementation of the experiment. The Scikit-Learn library was used to implement machine learning algorithms, while TensorFlow and Keras were utilized to build and implement the deep learning classifiers. Numerical computation and pre-processing of the dataset were carried out using the NumPy and Pandas libraries. Statistical analysis was performed using SciPy, and data visualization was conducted with the Matplotlib and Seaborn libraries. The hardware setup includes a PC with Intel$\circledR$ $\text {Core}^{\text {TM}}$ i5-10300H CPU @ 2.50GHz processor, 8GB RAM, 237 GB storage, and NVIDIA GeForce GTX 1650 GPU.

### Feature selection techniques

For feature selection, 11 selection techniques of various types such as filter and embedded methods are chosen and implemented on the dataset. These feature selection techniques such as information gain^13^, Chi-square test^5^, Fisher’s discriminant analysis^34^, variance threshold^35^, mean absolute difference^36^, dispersion ratio^37^, relief^17^, Lasso regularization^17^, random forest importance^35^, linear discriminant analysis^13^, and principal component analysis^38^ are considered for the research and the key feature selection techniques are described as follows.

#### Information gain

Information gain finds out how much information a feature is providing or the contribution of a feature in identifying the target value. It is the measure of reduction in entropy. Information gain talks about the relevance of a feature concerning the target variable. The pseudocode for calculating information gain is given in Algorithm 1.

**Algorithm 1:**
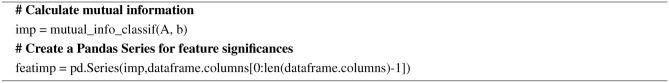
Pseudocode for calculating information gain

#### Chi-square test

The Chi-square test helps to test how categorical variables are related. It is done by comparing the observed values to the expected values. First, the chi-square is calculated using Eq. (1) between the target variable and the features, and then the desired features will be selected. The pseudocode for Chi-square test is given in Algorithm 2.

1 $$\begin{aligned} x^2=\sum \frac{(\text { Observed value }- \text { Expected value })^2}{\text { Expected value }} \end{aligned}$$

**Algorithm 2:**
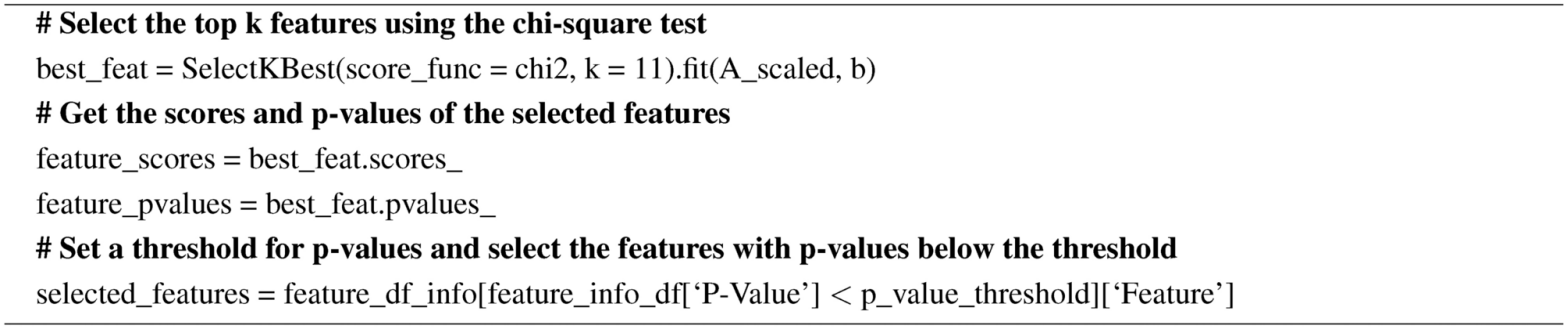
Pseudocode for Chi-square test

#### FDA

The FDA is widely used in feature selection for classification problems. A scalar combination of features that separates two or more classes is often aimed to be found in the FDA. The Fisher’s score is derived from the Fisher’s ratio which can be calculated using Eq. (2). The pseudocode for FDA is given in Algorithm 3.

2 $$\begin{aligned} F_i=\frac{\left( \text { Mean }_{\text {Class } 1}-\text { Mean }_{\text {Class } 2}\right) ^2}{\text { Variance }_{\text {Class } 1}+\text { Variance }_{\text {Class } 2}} \end{aligned}$$

$\text { Mean }_{\text {Class } 1}$ and $\text { Mean }_{\text {Class } 2}$ are the means of the feature Xi in both classes respectively. Similarly, $\text { Variance }_{\text {Class } 1}$ and $\text { Variance }_{\text {Class } 2}$ are the variances of the feature Xi in both classes respectively.

**Algorithm 3:**
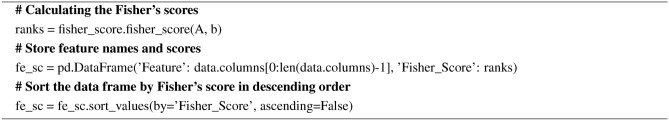
Pseudocode for FDA

#### MAD

The MAD and the variance threshold are similar, but the absence of a square makes the difference. It is calculated between each point and the mean which is also a scaled variant. The higher the MAD the higher the information carried by the feature or the better the discriminatory power. The mean absolute difference is calculated using Eq. (3). The pseudocode for MAD is given in Algorithm 4.

3 $$\begin{aligned} \operatorname {MAD}\left( x_i\right) =\frac{1}{n} \sum _{j=1}^n\left| x_{i j}-\operatorname {Mean}\left( x_i\right) \right| \end{aligned}$$

**Algorithm 4:**
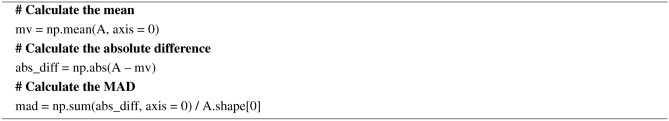
Pseudocode for the MAD

#### DR

DR for a given feature is the relationship between the arithmetic mean (AM) and the geometric mean (GM). The higher the dispersion ratio, the more relevant the feature is. The AM, GM, and DR are calculated using Eqs. (4), (5), and (6). The pseudocode for the dispersion ratio is given in Algorithm 5.

4 $$\begin{aligned} A M_i= & \frac{1}{n} \sum _{j=1}^n x_{i j} \end{aligned}$$

5 $$\begin{aligned} G M_i= & \left( \prod _{j=1}^n x_{i j}\right) ^{1 / n} \end{aligned}$$

6 $$\begin{aligned} D R= & \frac{A M_i}{G M_i} \end{aligned}$$

The DR holds for ${AM_i}$ $\ge$ ${GM_i}$ and lies in the interval $[1, +\infty )$.

**Algorithm 5:**
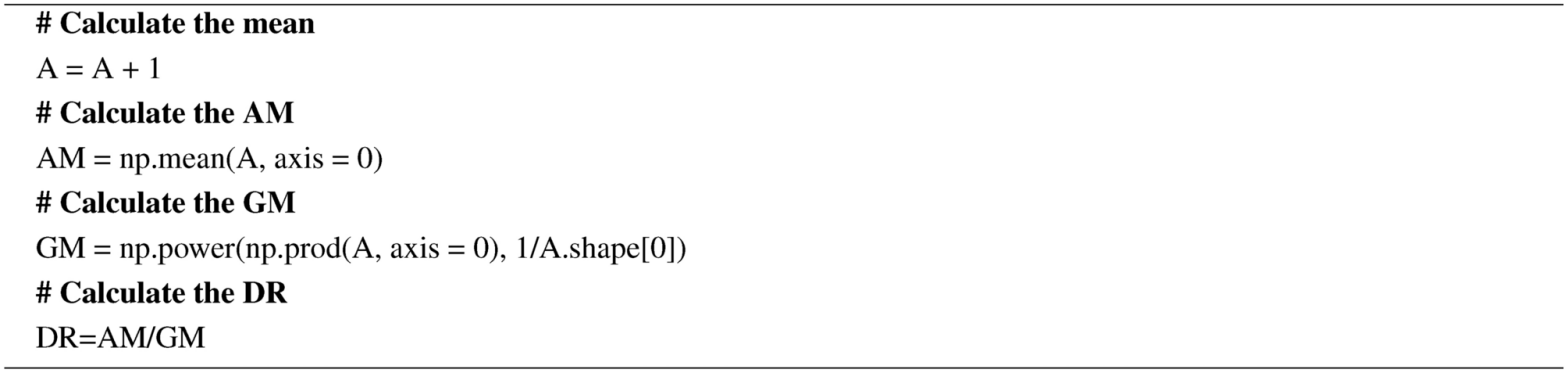
Pseudocode for the DR

#### Relief

In the relief technique, weights are assigned to the features according to how well they can differentiate between the instances of the same class and different classes. Relief and ReliefF are the two variants of the relief feature selection technique. Relief is used for binary classification and ReliefF is used for binary and multi-class classification. The weights are updated using the Eq. (7). The pseudocode for the Relief is given in Algorithm 6.

7 $$\begin{aligned} W_i=W_i-\frac{\left( x_i-x_i^{\prime }\right) }{k}+\frac{\sum _{c=1}^C \mid x_i-x_i^{\prime \prime }(C)}{C . k} \end{aligned}$$

Where *C* is the number of classes and $x_i^{\prime \prime }(C)$ is the value of feature *i* in the closest class C miss instance.

**Algorithm 6:**
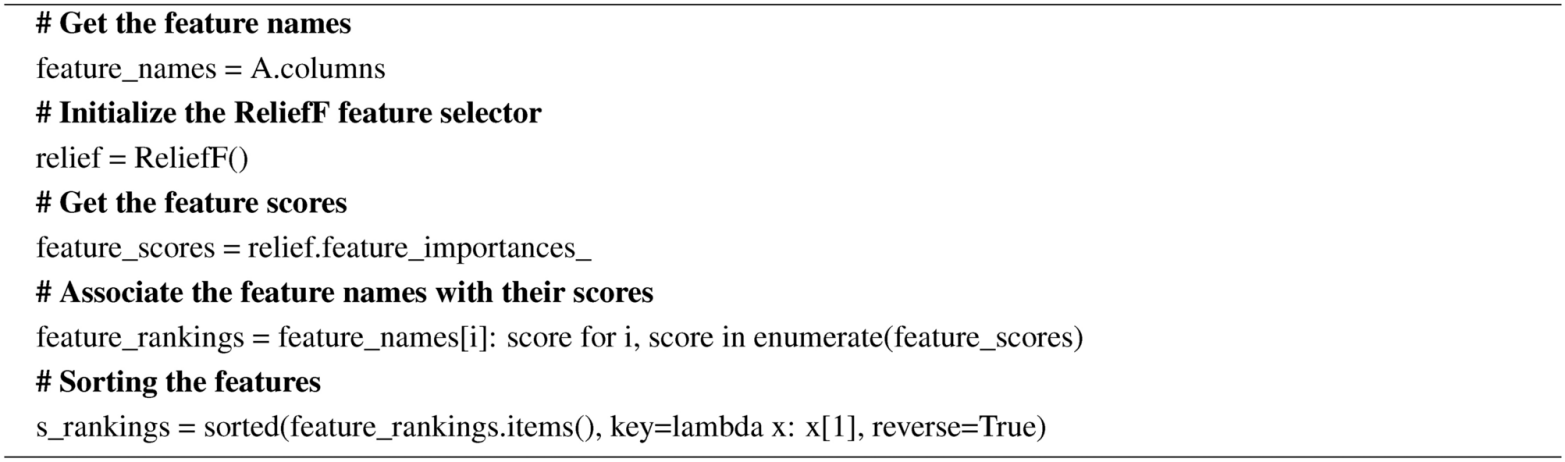
Pseudocode for the Relief

#### Random forest importance

Random forest aggregates predetermined decision trees. The ranking is based on how well the node’s purity is improving. The beginning of the trees contains the nodes with the largest decrement in impurity. Trees below a specific node can therefore be pruned to produce a set of the most significant features. The pseudocode for the Random Forest Importance is given in Algorithm 7.

**Algorithm 7:**
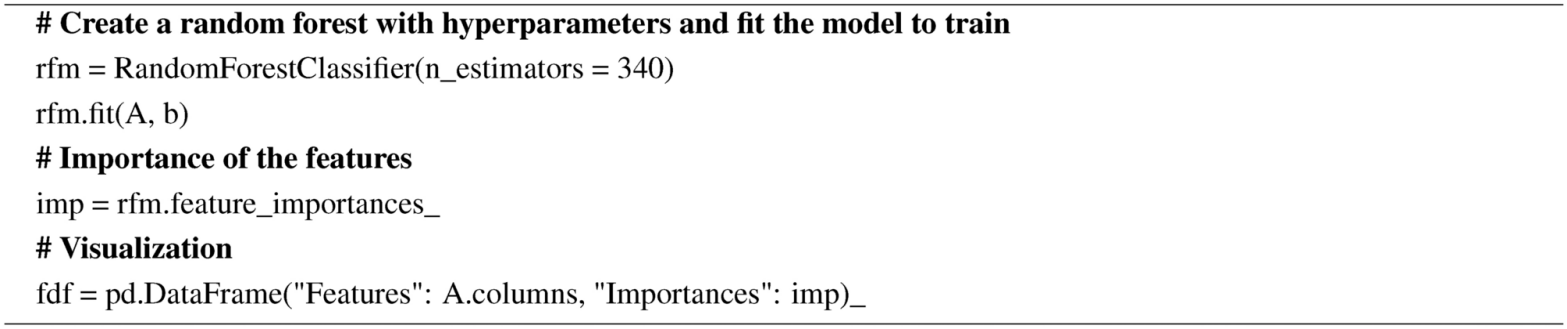
Pseudocode for the Random Forest Importance

### Classifiers

Classifiers such as boosting algorithms, ensemble learning algorithms, tree-based algorithms, and neural networks are applied in this experiment to predict heart disease, namely, logistic regression^39^, decision tree^6^, random forest^12^, k-nearest neighbors^6^, support vector machine^12^, gaussian naïve bayes^12^, extreme gradient boosting^40^, adaptive boosting^6^, stochastic gradient descent^41^, gradient boosting classifier^13^, extra tree classifier^42^, categorical boosting^40^, light gradient boosting machine^29^, multi-layered perceptron^43^, recurrent neural network^44^, long short-term memory^44^, gated recurrent unit^45^, bidirectional long short-term memory^44^, bidirectional gated recurrent unit^45^, convolutional neural network^36^, and hybrid model. The ‘window_size’ was set to 1 while implementing LSTM, GRU, Bi-LSTM, Bi-GRU, and CNN. The key techniques are described as follows.

#### RF

The class that serves as a mode for all the other classes will be the result of the random forest classifier’s training phase. The goal of the training is to reduce the error across the group of trees. When it comes to classification, the majority answer from each tree determines the random forest’s prediction. The Eq. (8) represents the predicted class. The pseudocode for RF is given in Algorithm 8.

8 $$\begin{aligned} {\hat{y}}=\arg \max _m\left( \sum _{n=1}^N I\left( y_{m n}=m\right) \right) \end{aligned}$$

Where $y_{m n}$ is the anticipated class for the $m^{th}$ sample in the $n^{th}$ tree.

**Algorithm 8:**
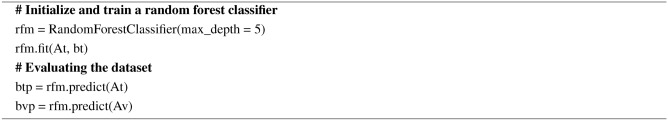
Pseudocode for RF

#### Extreme gradient boosting (XGBoost)

The XGBoost builds the model by combining the predictions of several weak learners. It optimizes the gradient descent algorithm for high efficiency and predictive power. The core of XGBoost is its objective function, which is optimized during the training process. The loss function for regression tasks is typically ‘mean_squared_error’ (MSE), and for classification tasks, it can be ‘Logloss’ (binary or multi-class) or other suitable functions. The objective function for a binary classification problem is given in Eq. (9). The pseudocode for XGBoost is given in Algorithm 9.

9 $$\begin{aligned} \operatorname {Obj}(\theta )=\sum _{i=1}^n L\left( y_i-{\hat{y}}_i\right) +\sum _{k=1}^K \Omega \left( f_k\right) \end{aligned}$$

Where the $\operatorname {Obj}(\theta )$ is the overall objective to be minimized, $L\left( y_i-{\hat{y}}_i\right)$ is the individual loss function, $\Omega \left( f_k\right)$ is the regularization term for each tree.

**Algorithm 9:**
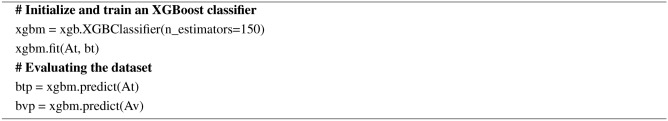
Pseudocode for XGBoost

#### AdaBoost

The ensemble learning algorithm AdaBoost builds the model as same as the XGBoost model. The performance of weak learners is improved by giving more importance or weight to the wrongly classified data points in each iteration. It adapts by emphasizing the training instances that are difficult to classify correctly. At its core is a formula given in Eq. (10) that calculates a weighted error rate for each classifier and assigns a “voting power” based on accuracy. The pseudocode for AdaBoost is given in Algorithm 10.

10 $$\begin{aligned} \varepsilon _t=\sum _{i=1}^N w_t^{(i)} I\left( h_t\left( x^{(i)} \ne y^{(i)}\right) \right) \end{aligned}$$

Where *N* is the total count of training instances,

$w_t^{(i)}$ represents the weight of instance *i* at iteration *t*

$h_t\left( x^{(i)} \ne y^{(i)}\right)$ is the prediction of the weak learner $h_t$ for instance $x^{(i)}$,

$y^{i}$ is the true label for instance *i*,

$I\left(h_t\left( x^{(i)} \ne y^{(i)}\right)\right)$ is an indicator function that equals one if the prediction is incorrect and zero otherwise.

The voting power of the weak learner at iteration *I* is calculated using Eq. (11).

11 $$\begin{aligned} \alpha _t=\frac{1}{2} \ln \left( \frac{1-\varepsilon _t}{\varepsilon _t}\right) \end{aligned}$$

**Algorithm 10:**
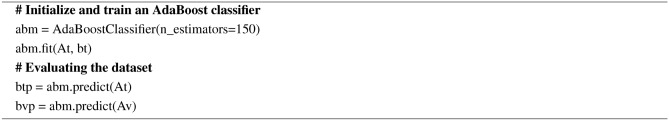
Pseudocode for AdaBoost

#### SGD

The basic principle of SGD is to update model parameters by calculating the gradient of the loss function concerning the parameters on a mini-batch of the training set in each iteration. The update rule for the model parameters in each iteration is given in Eq. (12). The pseudocode for SGD is given in Algorithm 11.

12 $$\begin{aligned} \theta _{t+1}=\theta _t-\eta _t \nabla J_t\left( \theta _t\right) \end{aligned}$$

Where $\nabla J_t\left( \theta _t\right)$ is the gradient of the objective function concerning $\theta$ at iteration *t*.

The loss function used for binary classification tasks in the SGD classifier is given in Eq. (13).

13 $$\begin{aligned} L(y, {\hat{y}})=-(y(\log {\hat{y}})+(1-y) \log (1-{\hat{y}})) \end{aligned}$$

Where *y* is the expected label and ${\hat{y}}$ is the predicted probability.

**Algorithm 11:**
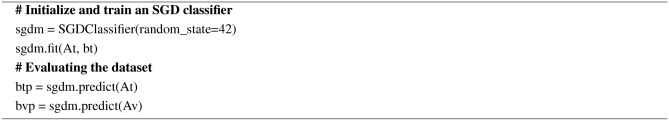
Pseudocode for SGD

#### GB

The gradient-boosting algorithm (GB) builds the model by combining the predictions of several weak learners. The loss function must be minimized, which is the sum of the individual losses for each instance. Each weak learner (typically a shallow decision tree) predicts a score for each instance. The class label of a new instance is obtained by summing the predictions from all weak learners as given in Eq. (14). The pseudocode for GB is given in Algorithm 12.

14 $$\begin{aligned} {\hat{y}}(x)=\sigma \left( \sum _{t=1}^T \eta h_t(x)\right) \end{aligned}$$

Where $h_t(x)$ is the prediction of the weak learner at iteration *t*, $\eta$ is the learning rate.

**Algorithm 12:**
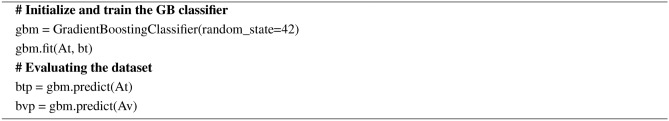
Pseudocode for GB

#### Extra tree classifier (ETC)

The ETC belongs to decision tree-based classifiers. Some additional randomness is introduced by the extra trees which reduce variance and overfitting. It is very similar to a random forest. The final prediction will be obtained by aggregating the individual predictions of all trees. A majority vote is considered for classification tasks and averaging the predictions is done for regression tasks. The pseudocode for ETC is given in Algorithm 13.

**Algorithm 13:**
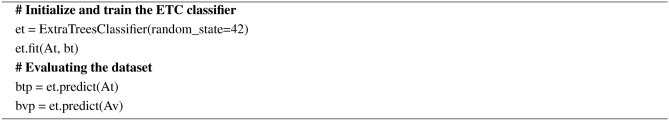
Pseudocode for ETC

#### Categorical boosting (CatBoost)

CatBoost is known for its efficient handling of categorical features, better performance, and high computational efficiency. Its major use case is that it can handle categorical features without one-hot encoding. Like the other gradient boosting algorithms, CatBoost also uses a gradient boosting framework and employs ‘ordered boosting’ to deal with the categorical variables. The final prediction is given by the Eq. (15).The pseudocode for CatBoost is given in Algorithm 14.

15 $$\begin{aligned} {\hat{y}}(x)=\sigma \left( \sum _{t=1}^T F_t(x)\right) \end{aligned}$$

Where $F_t(x)$ the $t^{th}$ tree’s prediction for input *x*

**Algorithm 14:**
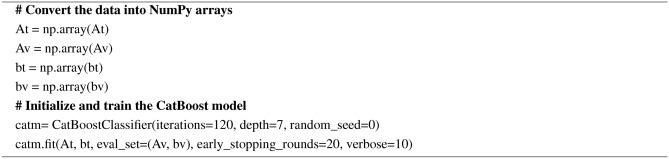
Pseudocode for CatBoost

#### Light gradient boosting machine (LightGBM)

Microsoft developed LightGBM. It is mostly suitable for large datasets and comes under the category of gradient boosting algorithms. The characteristics viz., efficiency, speed, and scalability set LightGBM separately from the other gradient-boosting algorithms. Like CatBoost, LightGBM fits each new tree to the negative gradient of the loss function. The ‘Logloss’ function is used for classification tasks and the ‘mean_squared_error’ function is used for regression tasks. The pseudocode for LightGBM is given in Algorithm 15.

**Algorithm 15:**
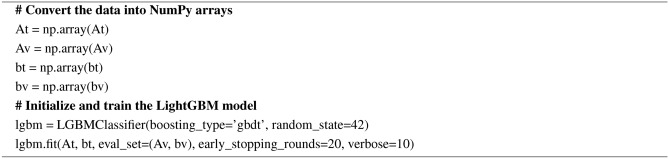
Pseudocode for LightGBM

#### MLP

**Fig. 2:**
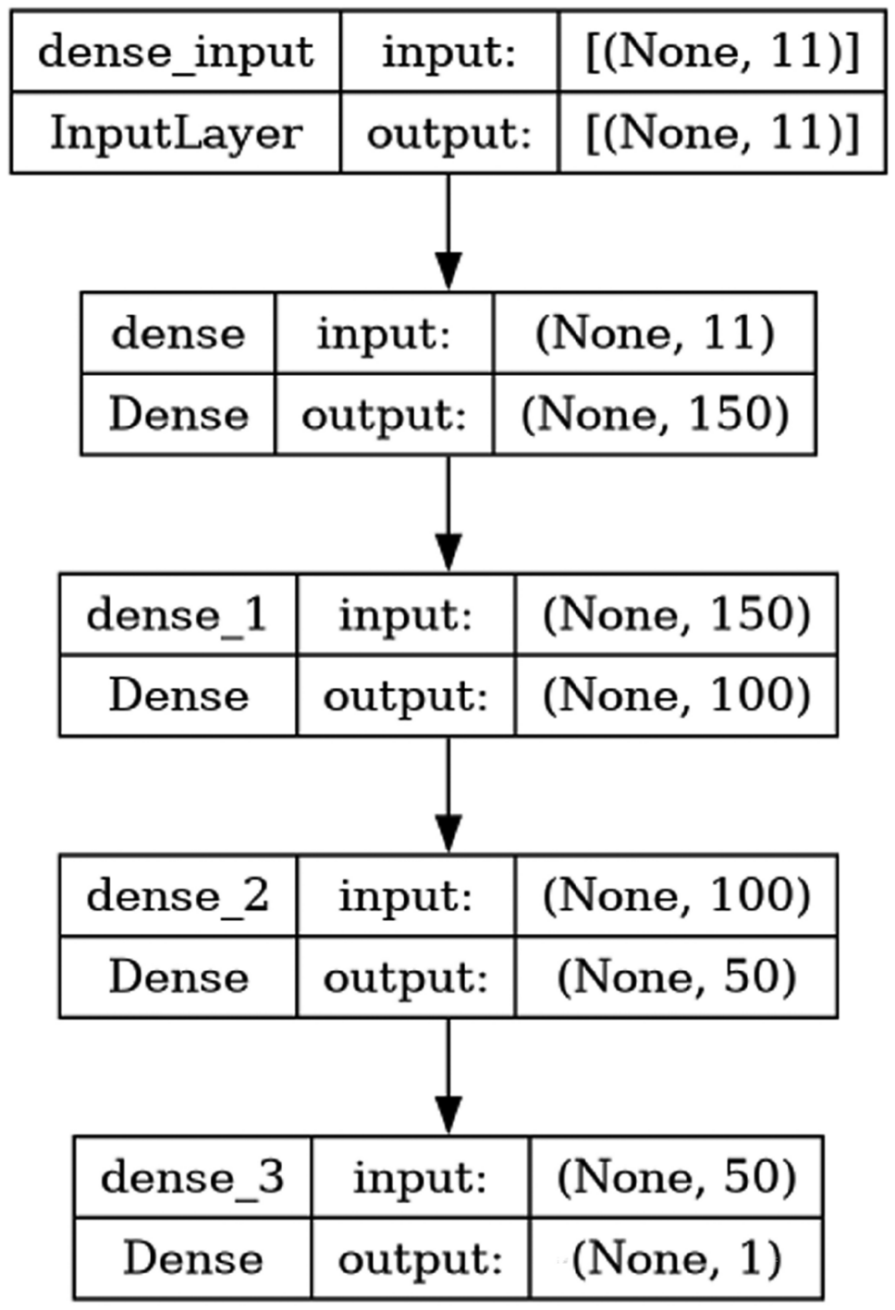
Model architecture of MLP.

MLP is an ANN that consists of more than one hidden layer. The model consists of hidden layers with 150, 100, and 50 neurons each and a ReLU activation function, which adapts to the input dimensions. The final layer employs sigmoid activation with a single neuron, offering probability predictions. The architecture of MLP is shown in Fig. 2, and The pseudocode for MLP is given in Algorithm 16.

**Algorithm 16:**
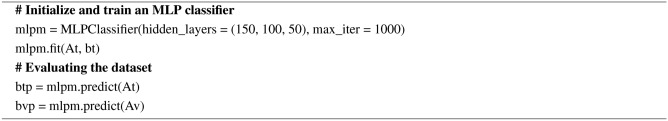
Pseudocode for MLP

#### RNN

**Fig. 3:**
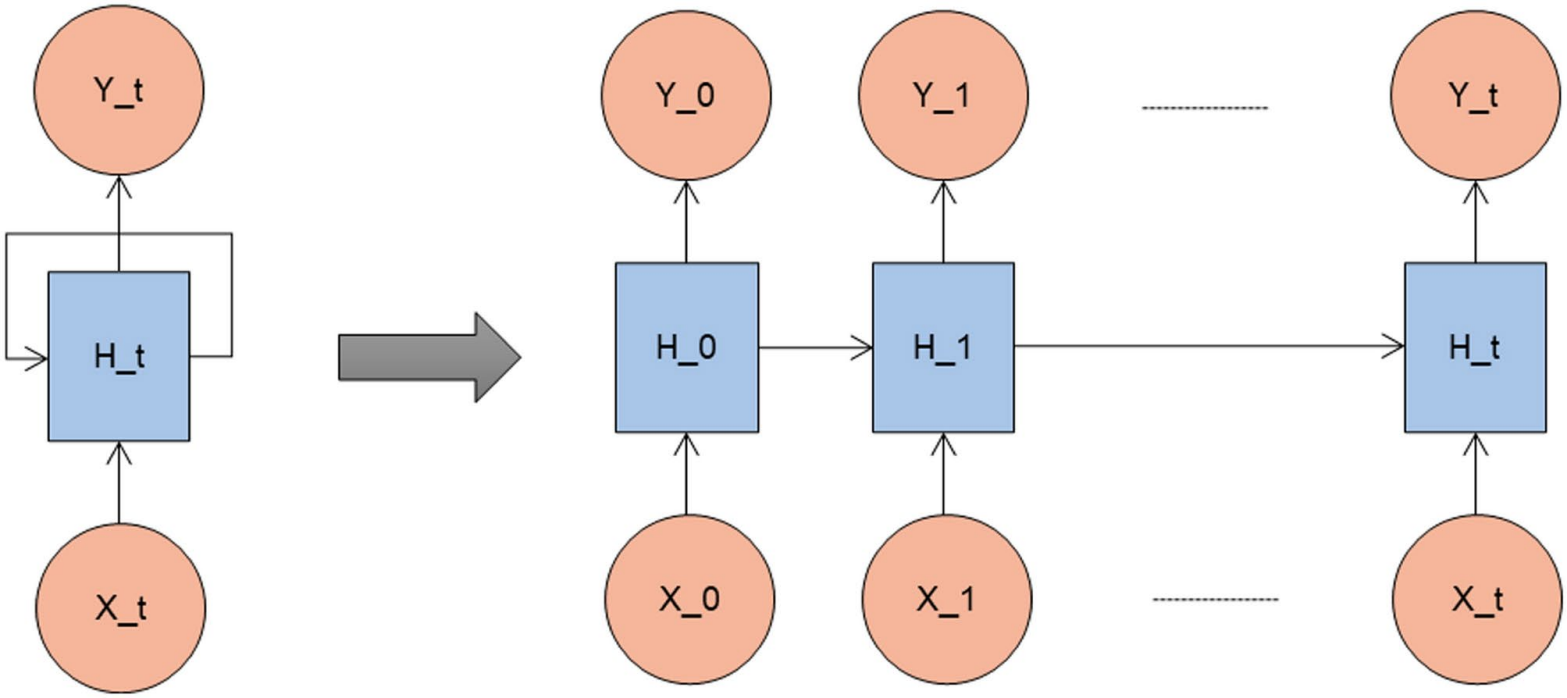
Structure of RNN.

**Fig. 4:**
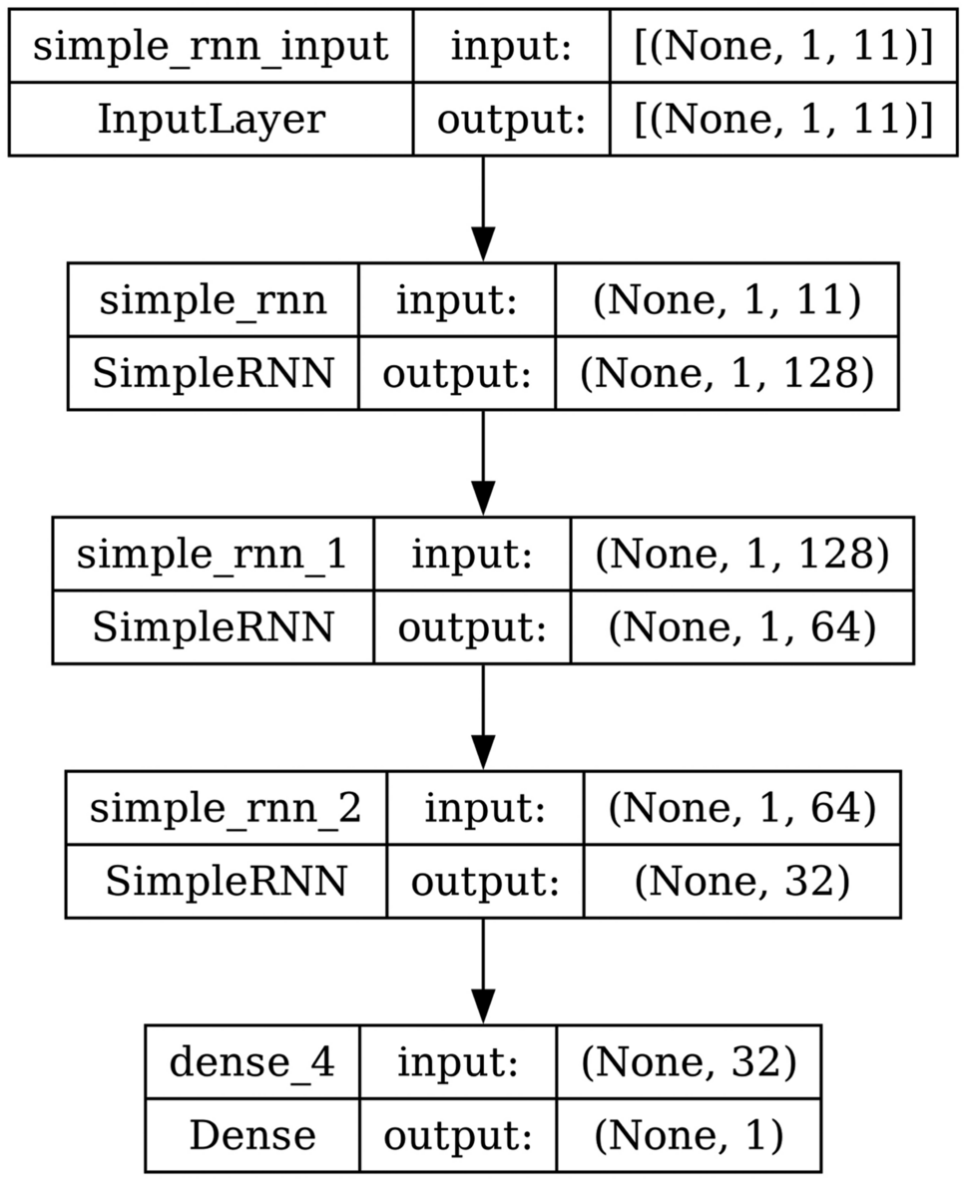
Model architecture of RNN.

A recurrent layer in an RNN is a layer that sequentially processes a sequence of inputs. Time series forecasting is one of the major applications of the RNNs, where the full input shape is 3D. It means batch size, time steps, and dimensionality of the inputs at every time step. A recurrent layer is composed of a single memory cell, which is used repeatedly to compute the outputs. A memory cell is a small neural network. It can be a simple dense layer or a complex memory cell such as an LSTM or GRU cell. The structure and the architecture of RNN are shown in Figs. 3 and 4 respectively. In this, the first layer outputs a sequence which is fed to the second recurrent layer and then to the third layer. In the third layer, ‘return_sequences’ is set to false and only outputs the final time step. The last output is sent to the dense layer.

#### LSTM

**Fig. 5:**
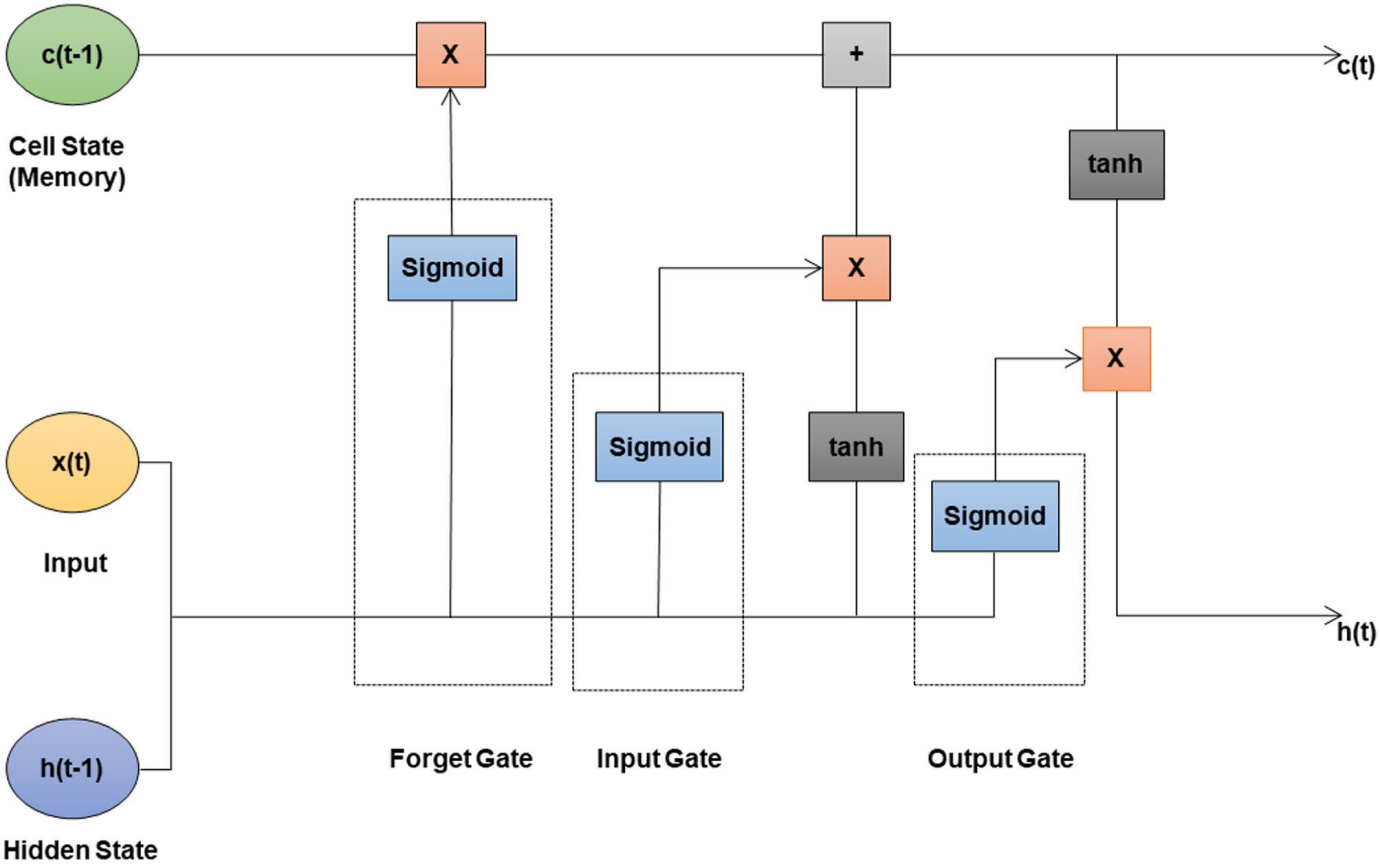
Structure of LSTM.

**Fig. 6:**
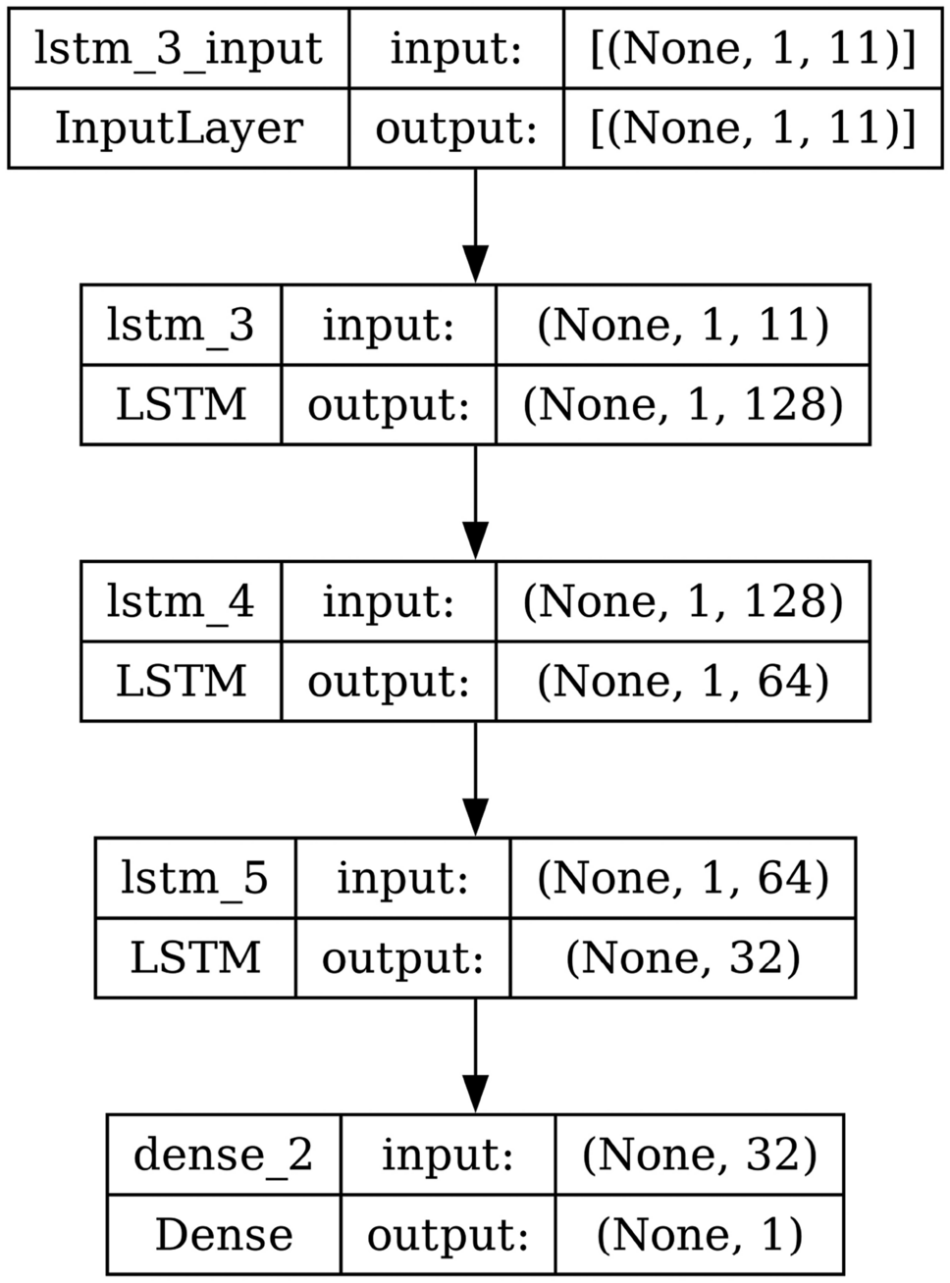
Model architecture of LSTM.

LSTM consists of three gates and one memory cell. During training, the forget gate will gradually learn when to erase part of the long-term memory. For example, if the long-term memory holds information that there is a string upward trend and the forget gate sees a severe drop in the inputs at the current time step, then it will probably learn to erase that part of the long-term memory since the upward trend is over. In short, the forget gate learns when to forget things and when to preserve them. The input gate learns when it should store information in the memory and the output gate learns which part of the long-term state factor it should output at each time step. The memory cell learns when it should forget the memories, when it should store new ones, and which part of the long-term state vector it should output at each time step. LSTM has a much longer memory than RNN. The structure and architecture of LSTM are shown in Figs. 5 and 6 respectively.

#### GRU

**Fig. 7:**
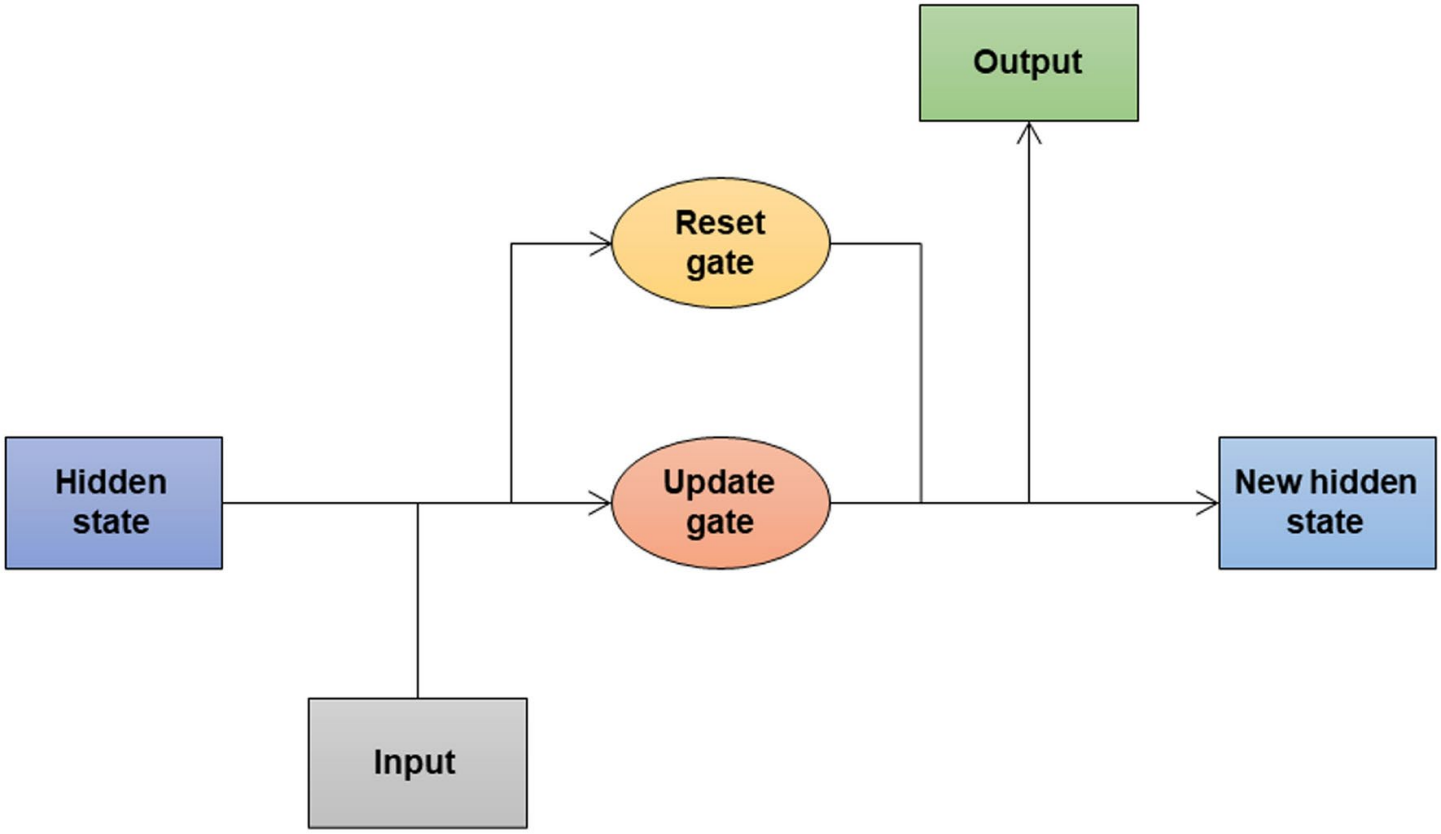
Structure of GRU.

**Fig. 8:**
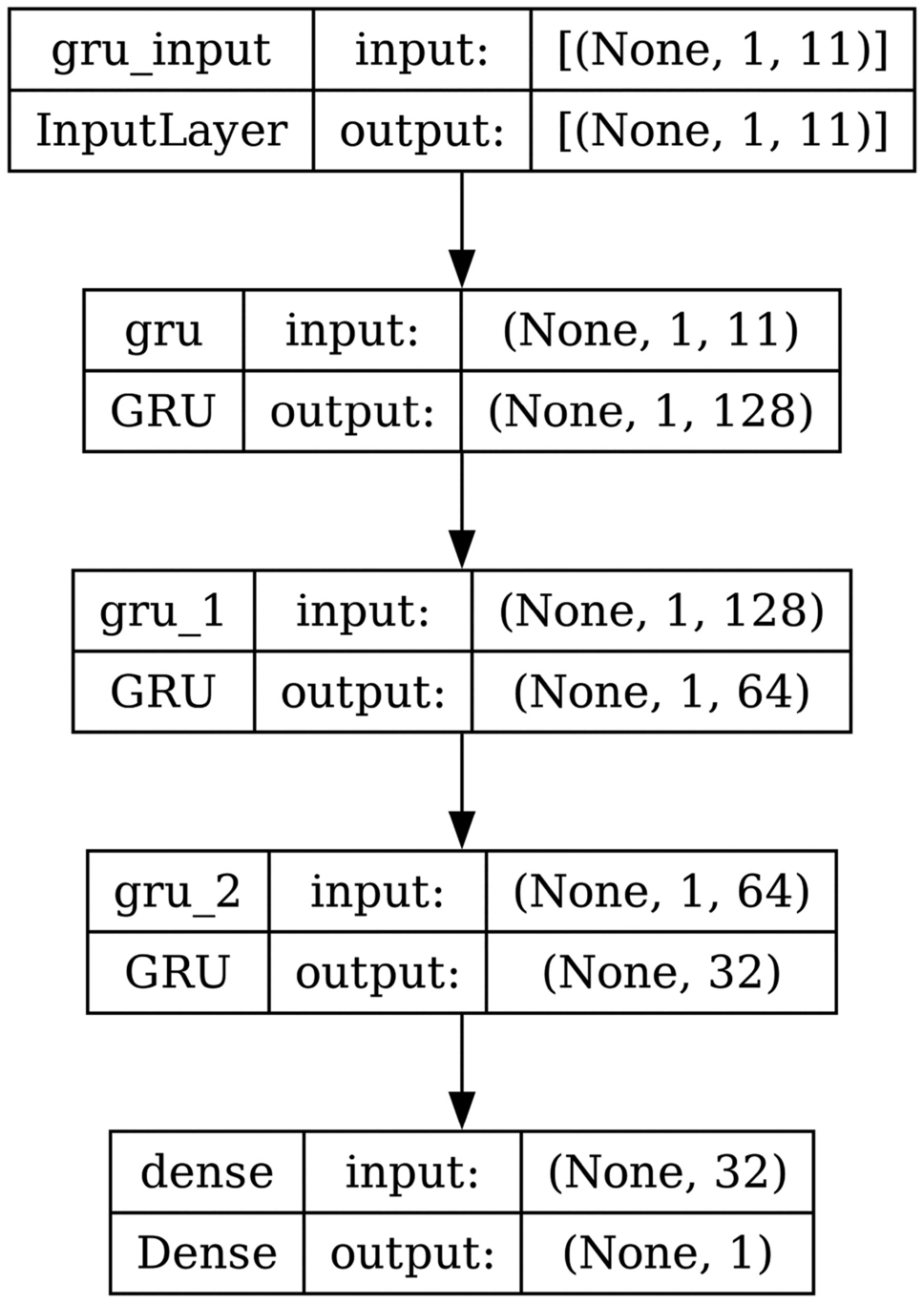
Model architecture of GRU.

A GRU processes sequential input data like LSTM. It has a reset gate and an update gate. These gates decide what information to remove and what to keep. GRU is like LSTM, but it doesn’t maintain a cell state. The structure and the architecture of GRU are shown in Fig. 7 and Fig. 8 respectively.

#### Bi-LSTM

**Fig. 9:**
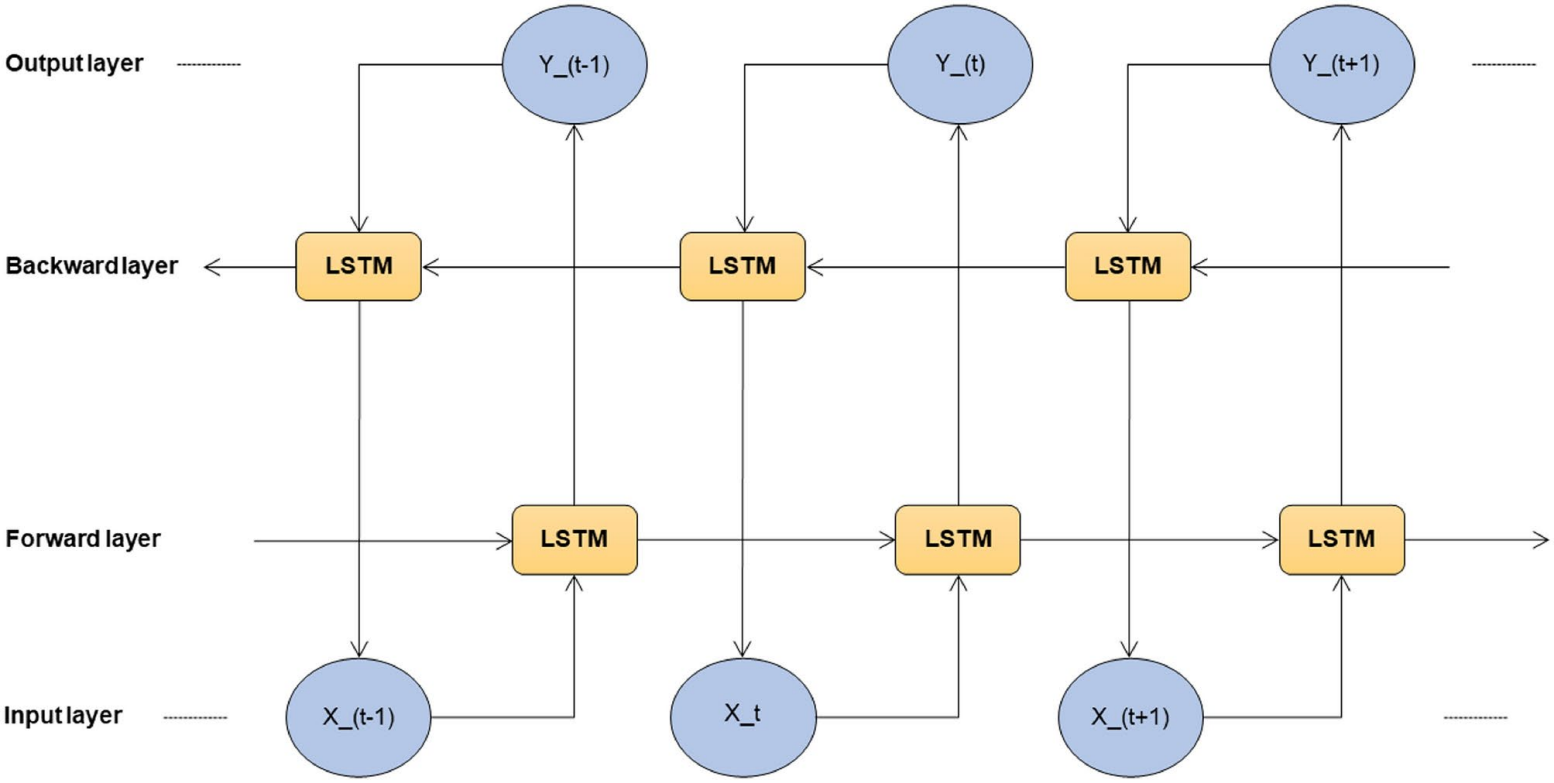
Structure of Bi-LSTM.

**Fig. 10:**
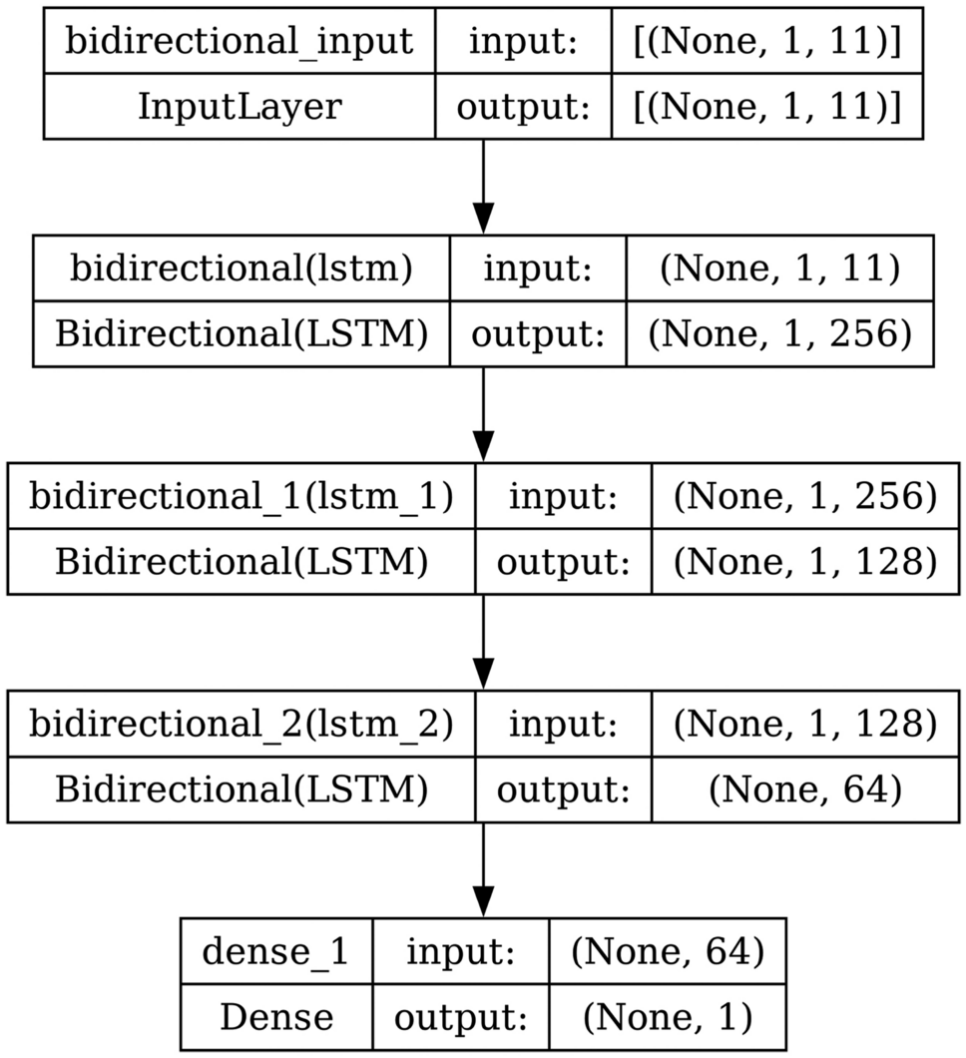
Model architecture of Bi-LSTM.

A Bi-LSTM consists of two LSTM models. The first model takes the input as it is and learns the sequence. The second model takes the backward sequence as input and learns the sequence. Bi-LSTM is more complex than Bi-GRU, which makes the training longer and has more parameters due to its complex nature. It is known for its long-term dependencies. The structure and the architecture of Bi-LSTM are shown in Figs. 9 and 10 respectively.

#### Bi-GRU

**Fig. 11:**
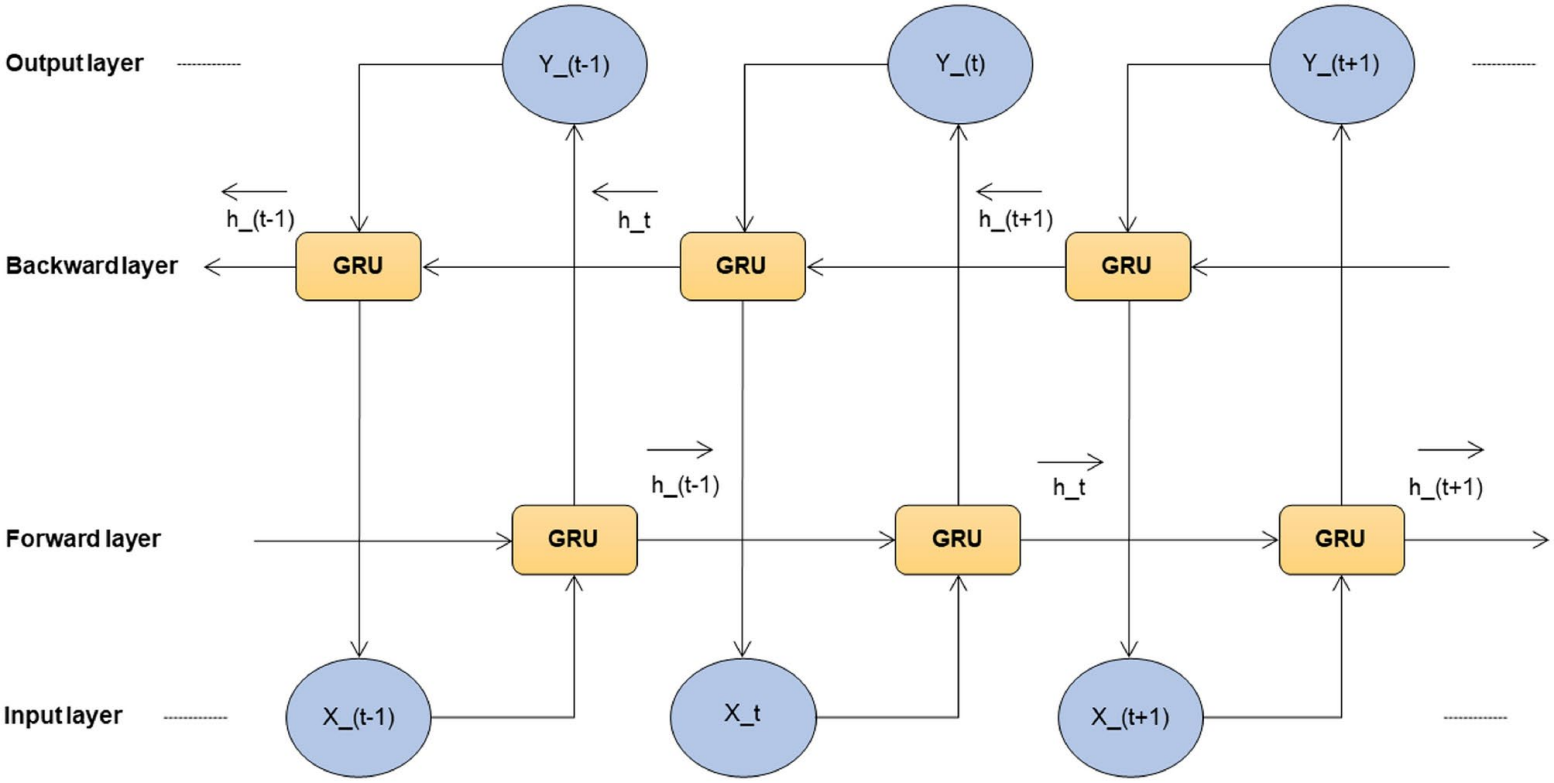
Structure of Bi-GRU.

**Fig. 12:**
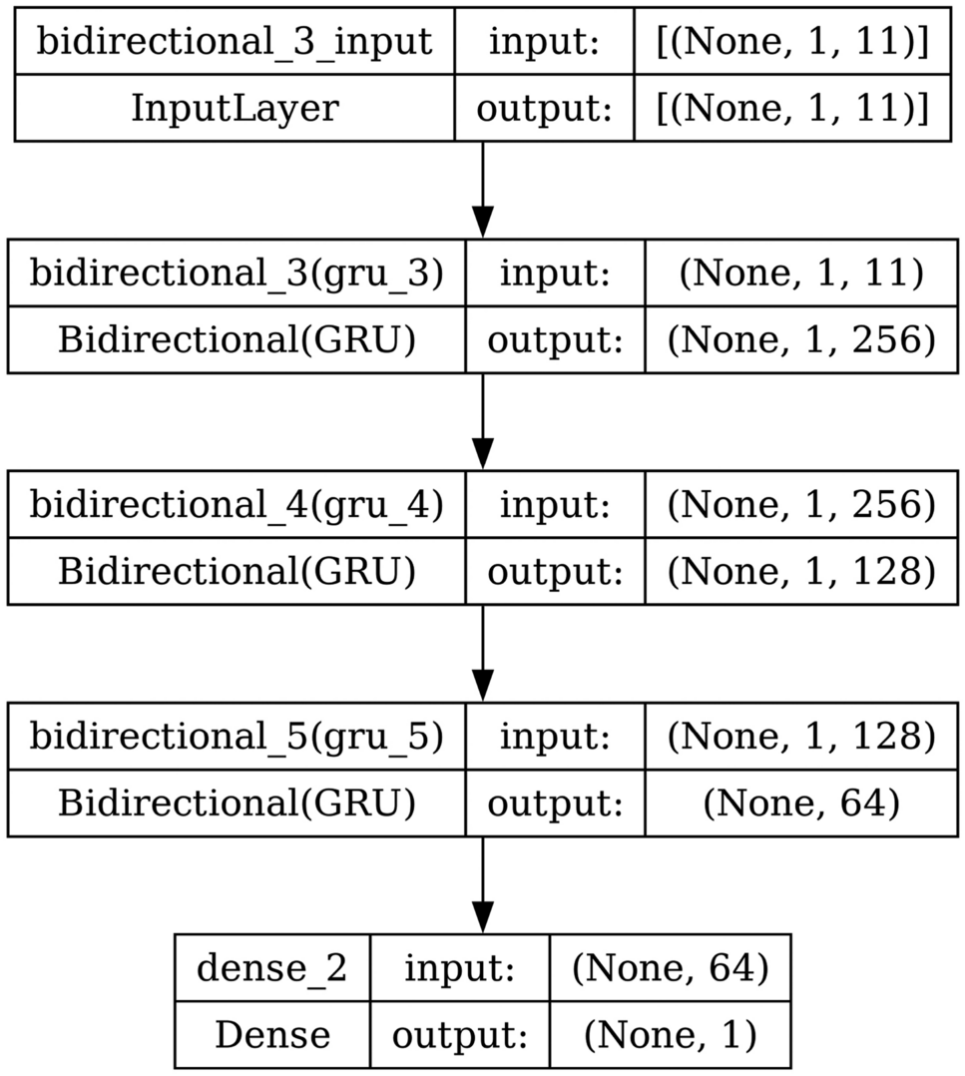
Model architecture of Bi-GRU.

Bi-GRU has a more straightforward structure since it merges the hidden state and the memory into one state. It uses two gates, the update gate, and the reset gate, but lacks the explicit memory cell found in LSTM. Due to its simpler structure, training takes place a bit faster and is computationally preferable. It is suitable where a balance between the model performance and computational efficiency is required. The structure and the architecture of Bi-GRU are shown in Figs. 11 and 12 respectively.

#### CNN

**Fig. 13:**
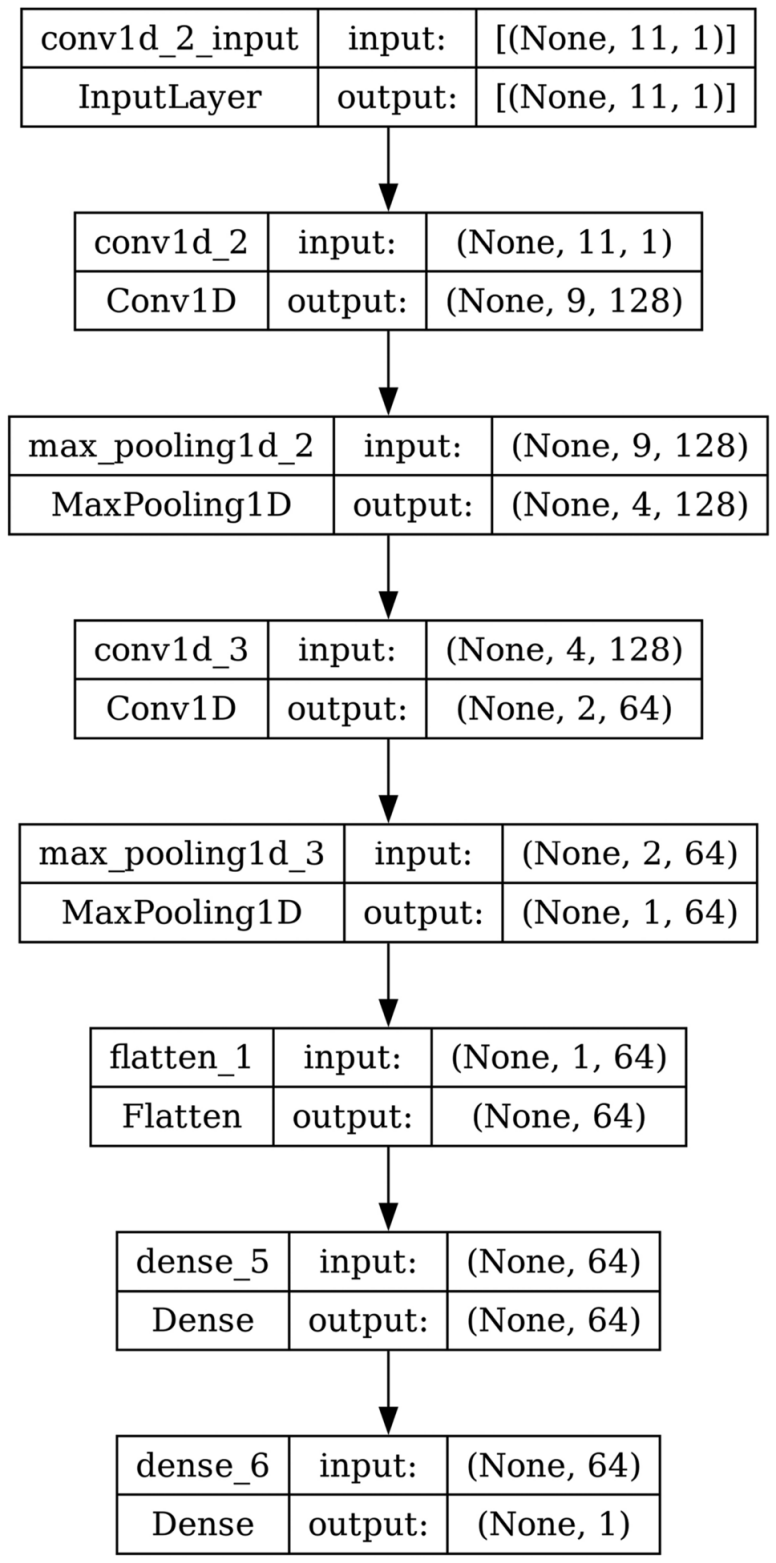
Model architecture of CNN.

A 1D CNN is exactly like a 2D CNN except it slides filters across just 1D instead of sliding them across 2Ds, typically the breadth and height of an image. Similar to an RNN, a 1D CNN can take input of any length. It has no memory at all. The computation of output is done based on the window of input time steps and the kernel size. Instead of using a single layer with a large kernel, it is better to stack multiple CNN layers each with a small kernel. The architecture of CNN is shown in Fig. 13.

#### Hybrid model

**Fig. 14:**
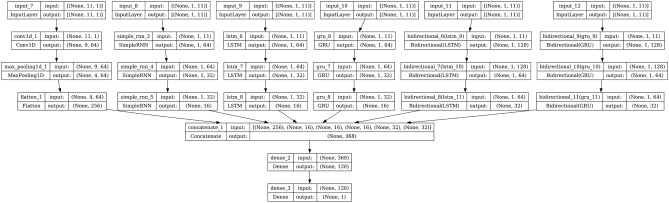
Model architecture of hybrid model.

The hybrid model is built using the 6 neural networks namely CNN, RNN, LSTM, GRU, Bi-LSTM, and Bi-GRU. A total of 3 hidden layers were introduced in the 6 individual models. After concatenation of the 6 models, a dense layer with 128 neurons and the ‘ReLU’ activation function was introduced. The output layer was made up of a dense layer with one neuron and a sigmoid activation function. The model architecture is shown in Fig. 14.

## Results and discussion

This section is classified into three sub-sections viz., the results of feature selection techniques, the results of classifiers without feature selection, and the results of classifiers with feature selection.

### Results of feature selection techniques

This section discusses how the feature selection is performed, the individual ranks and scores of the features, and how the desired features are selected for implementation.

#### Information gain

**Fig. 15:**
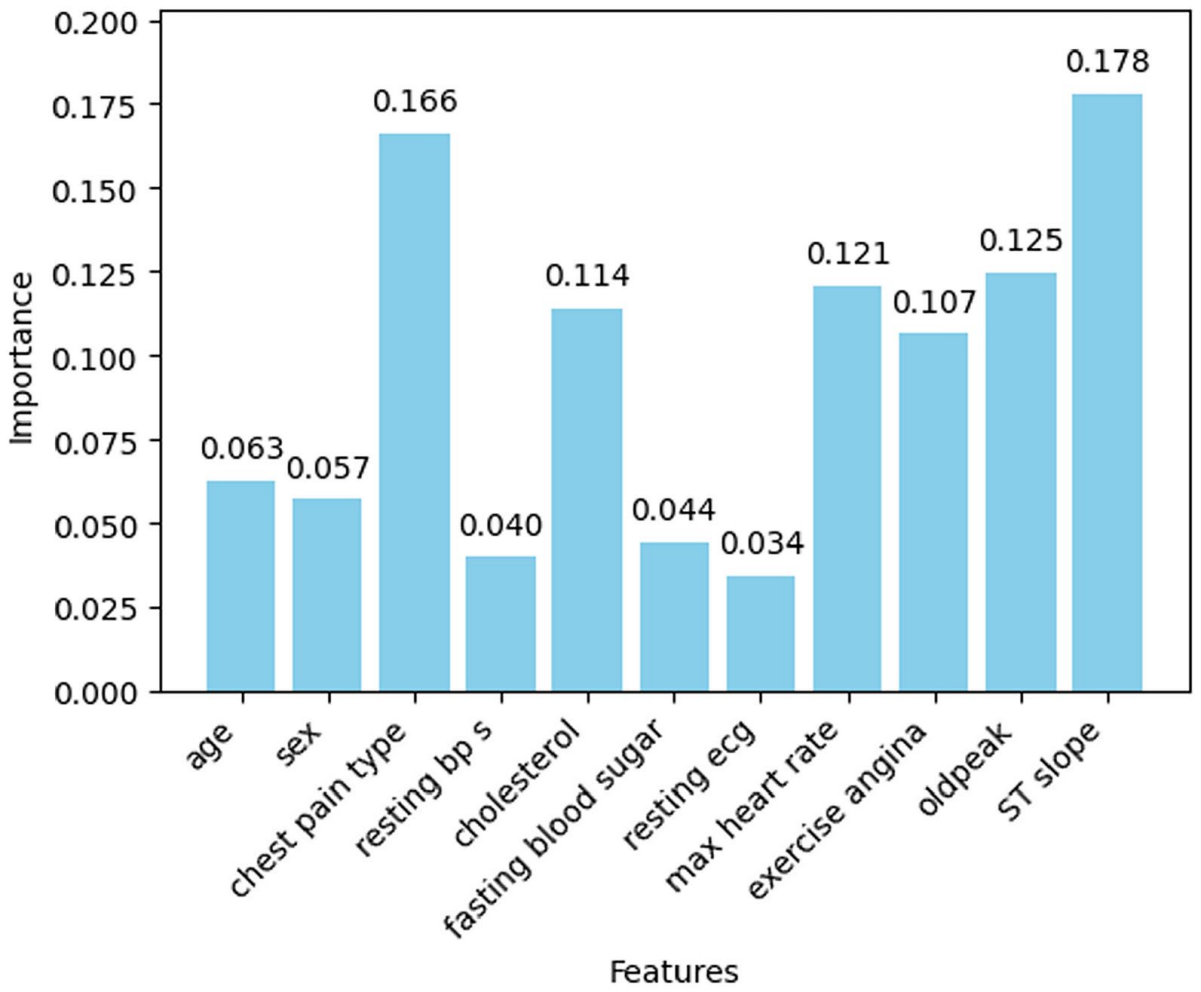
Feature importance of information gain.

The importance scores of all features are visualized in Fig. 15. From this, it is observed that almost half of the feature importance scores are above 0.1 and a few of them are below 0.1. The features with importance scores below 0.1 are removed as these features do not contribute much to heart disease prediction. So, the features age (0.063), sex (0.057), resting bp s (0.040), fasting blood sugar (0.044), and resting ecg (0.034) are removed from the dataset. Now, the prediction is done based on the features mentioned in Table 4 and accordingly tabulated the feature importance scores.

**Table 4:** Feature importance of information gain.

S. No.	Features	Importance scores
1	Chest pain type	0.166169
2	Cholesterol	0.114232
3	Max heart rate	0.120618
4	Exercise angina	0.106628
5	Oldpeak	0.124690
6	ST slope	0.178056

#### Chi-square test

**Fig. 16:**
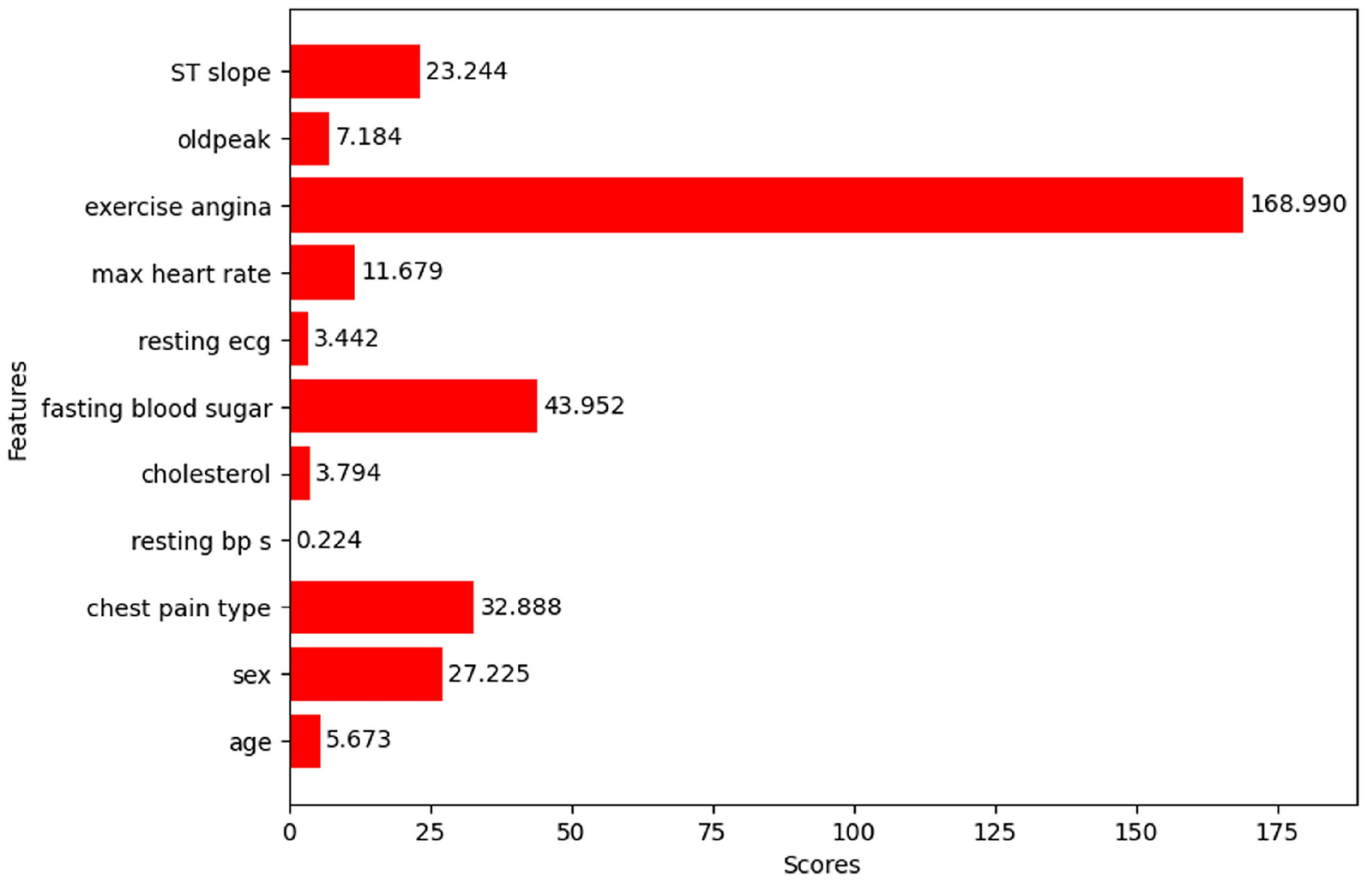
Chi-square test results of the features.

The respective feature scores of the features are shown in Fig. 16. The p-values tell whether the results are significant or not. First, the chi-square test was performed on the dataset, and the respective scores and p-values were calculated. The desired features were selected based on the p-values. Three types of thresholds can be chosen for p-values. 0.01 is the strong threshold, 0.001 is the medium threshold, and 0.005 is the weak threshold. The features with p-values below the threshold are selected. The medium and weak thresholds gave the same set of features. The features selected by strong, medium, and weak thresholds are tabulated in Table 5 and Table 6.

**Table 5:** Features selected by strong threshold.

S. No.	Features	Score	p-value
1	Sex	27.225	1.81e-07
2	Chest pain type	32.888	9.76e-09
3	Fasting blood sugar	43.952	3.37e-11
4	Max heart rate	11.679	6.32e-04
5	Exercise angina	168.990	1.23e-38
6	Oldpeak	7.184	7.36e-03
7	ST slope	23.244	1.43e-06

**Table 6:** Features selected by medium and weak thresholds.

S. No.	Features	Score	p-value
1	Sex	27.225	1.81e-07
2	Chest pain type	32.888	9.76e-09
3	Fasting blood sugar	43.952	3.37e-11
4	Max heart rate	11.679	6.32e-04
5	Exercise angina	168.990	1.23e-38
6	ST slope	23.244	1.43e-06

#### FDA

**Fig. 17:**
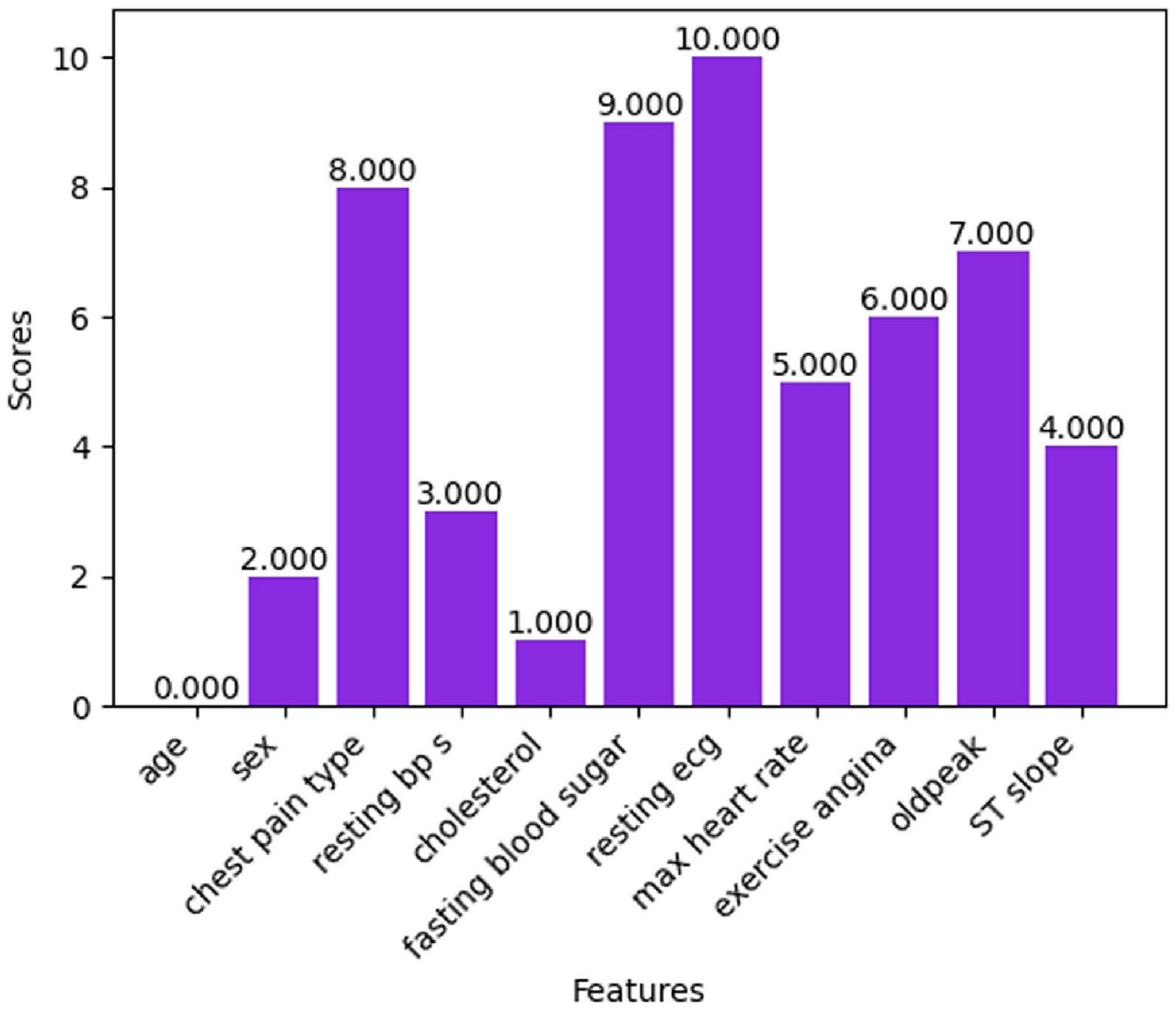
Fisher’s scores.

Fisher’s scores of the features are shown in Fig. 17. The considered heart disease dataset is divided into three sets with the top 7, top 5, and top 3 features based on the descending order of the Fisher’s scores, and then the classifiers are applied to the features for the heart disease prediction. The first set consists of the features resting ecg, fasting blood sugar, chest pain type, oldpeak, exercise angina, max heart rate, and ST slope. The second set consists of the features resting ecg, fasting blood sugar, chest pain type, oldpeak, and exercise angina. The third set consists of the features resting ecg, fasting blood sugar, and chest pain type.

#### Variance threshold

**Fig. 18:**
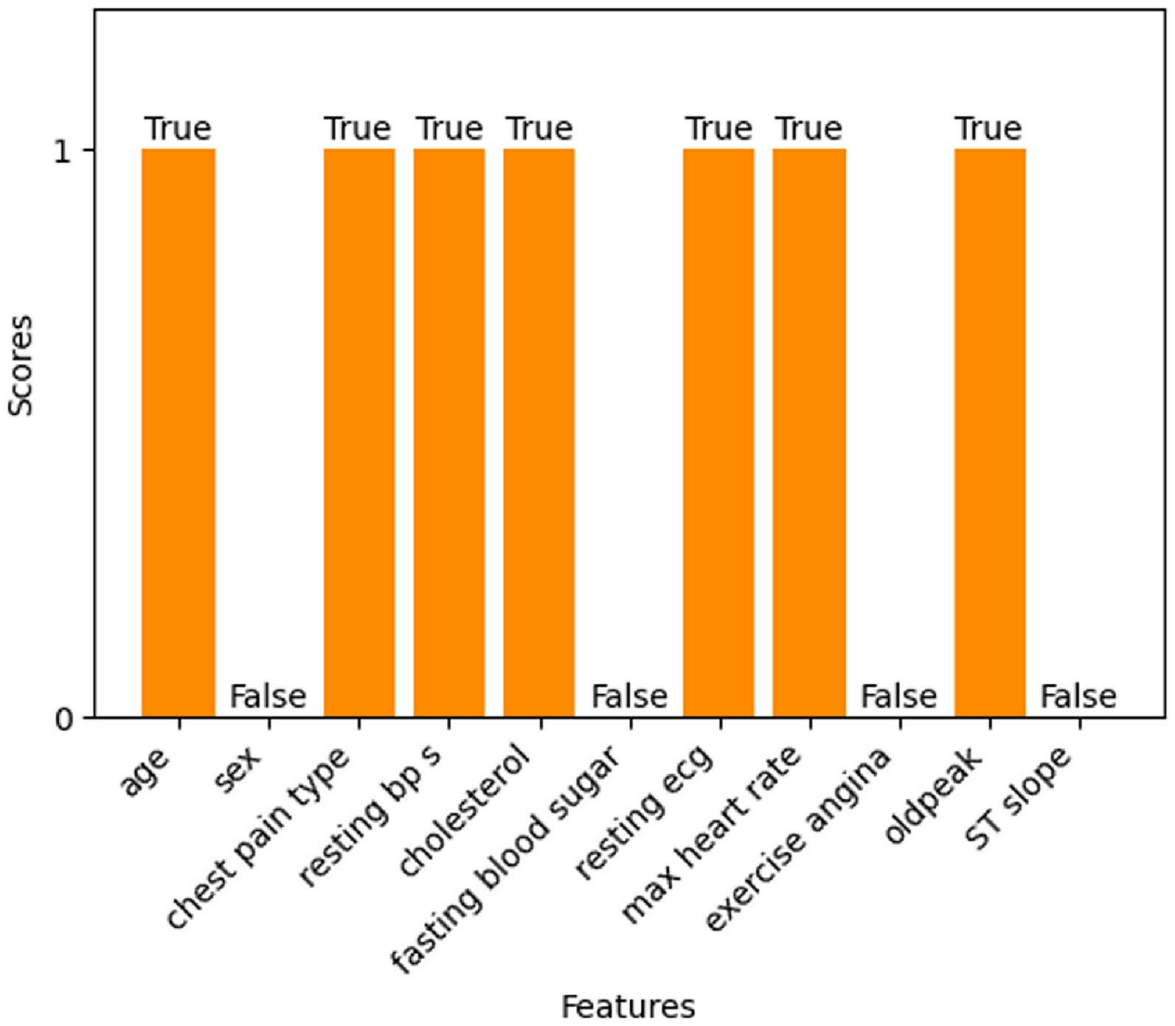
Features selected by variance threshold.

The features with zero variance will be removed by the variance threshold feature selection technique. A threshold is set for the variance. Three types of variance thresholds can be considered. The value of 0.01 is the small or weak threshold, 0.1 is the medium threshold, and 0.5 is the strong threshold. In this experiment, the threshold value of 0.5 is considered to obtain better results. From Fig. 18, it is observed that features such as sex, fasting blood sugar, exercise angina, and ST slope are shown as ‘False’ which indicates that these 4 features have lower variance than the variance threshold, contribute no information, and those features can be removed. The final feature set consists of features with a variance more than the variance threshold namely age, chest pain type, resting bp s, cholesterol, resting ecg, max heart rate, and oldpeak.

#### MAD

**Fig. 19:**
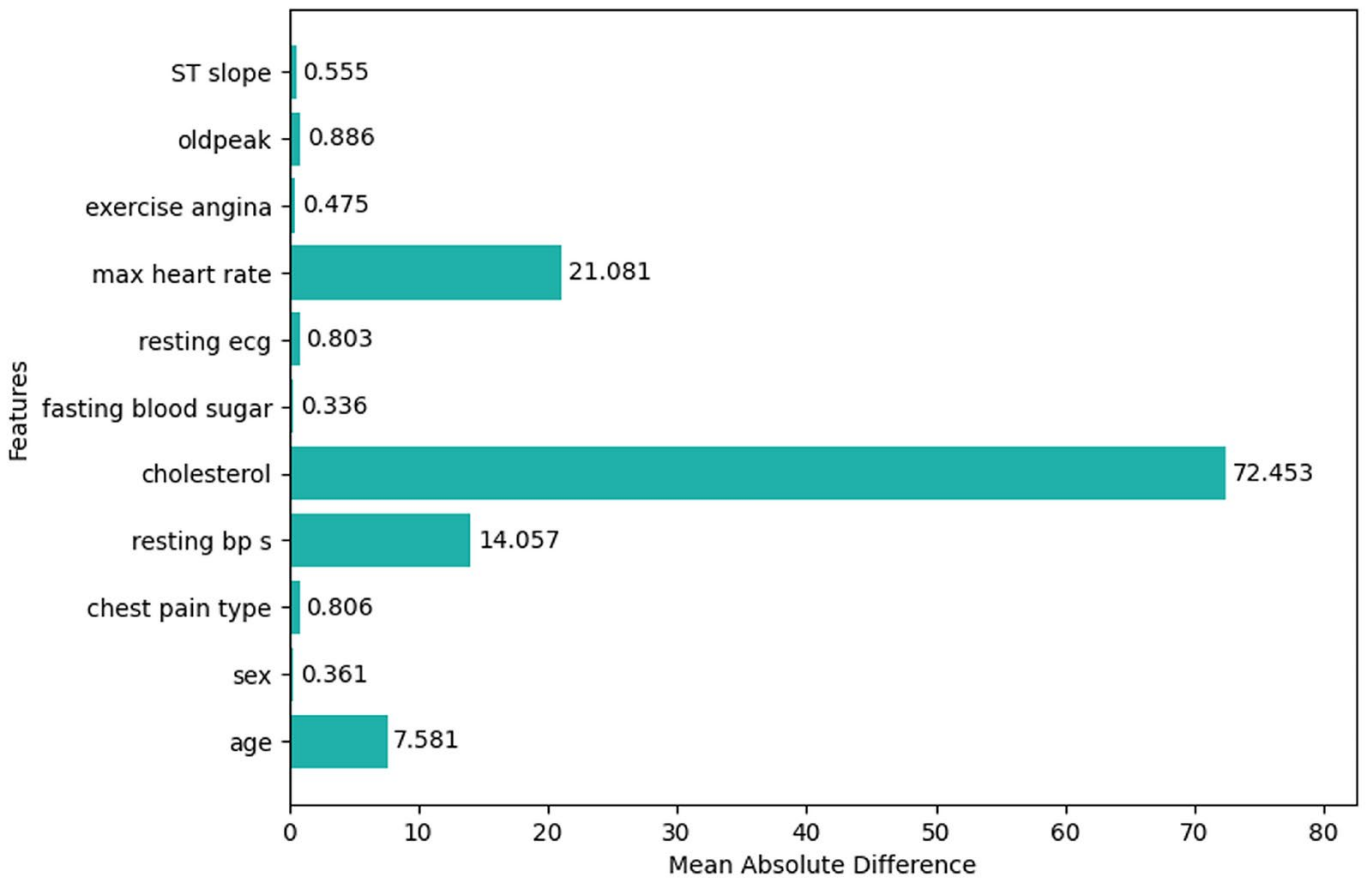
Mean absolute difference of the features.

A very small number of features shown in Fig. 19 have MAD above 1.0 which depicts that these features namely sex, chest pain type, fasting blood sugar, resting ecg, exercise angina, oldpeak, and ST slope have weak discriminatory power, so they are no longer be used for the prediction of heart disease presence. Features like age, resting bp s, cholesterol, and max heart rate have MAD 7.581, 14.057, 72.453, and 21.081 respectively. Out of these 4 features, cholesterol has a large MAD, which tells that this feature carries a larger amount of information, or this feature has a strong discriminatory power. It is also observed that these 4 features are present along with other features in the final feature set by variance threshold which depicts that the absence of a square makes the difference.

#### DR

**Fig. 20:**
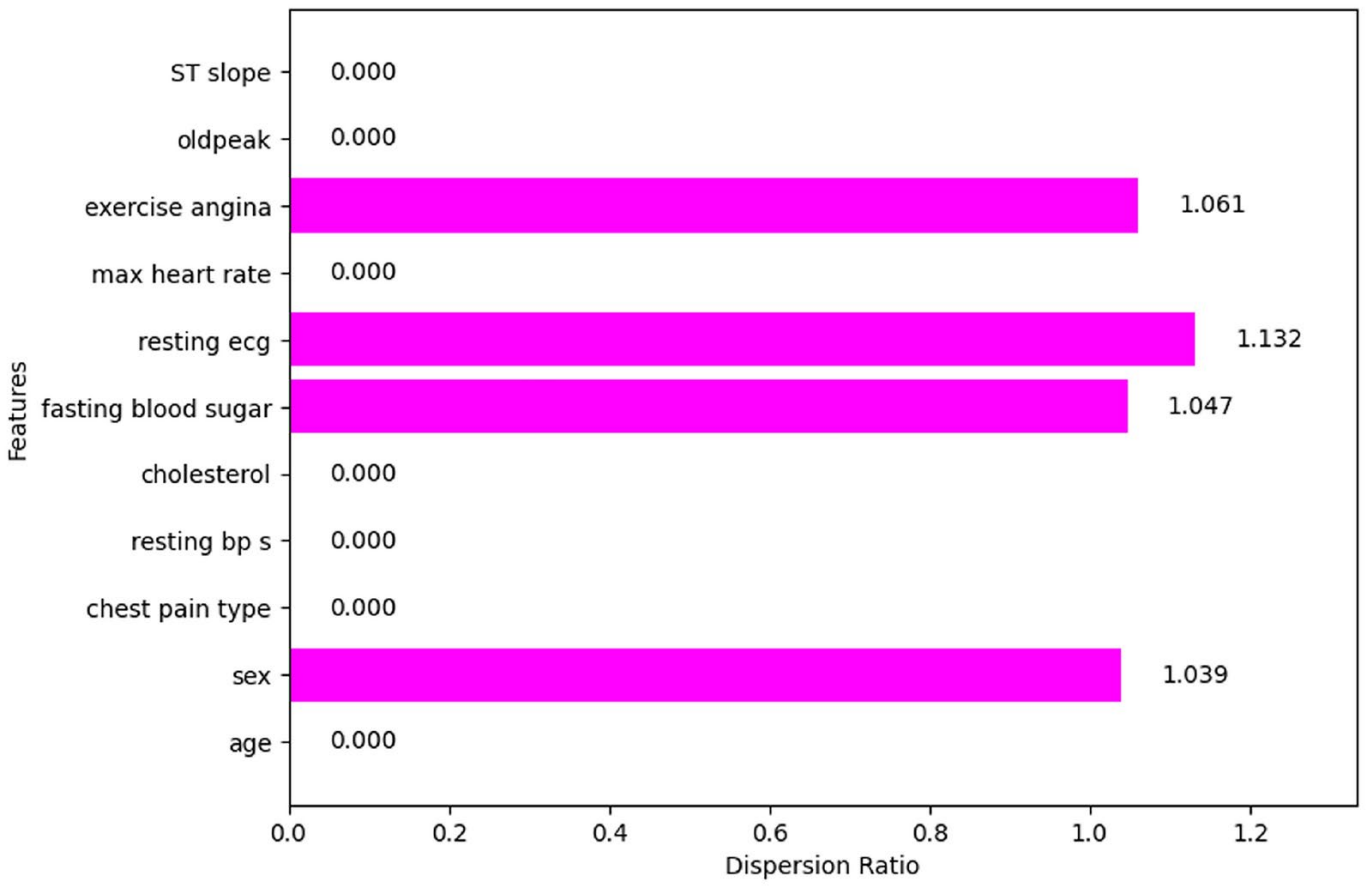
Dispersion ratio of the features.

A very small number of features have a dispersion ratio of more than one namely sex (1.039), fasting blood sugar (1.047), resting ecg (1.132), and exercise angina (1.061) are shown in Fig. 20. This shows that the features with a zero-dispersion ratio such as age, chest pain type, resting bp s, cholesterol, max heart rate, oldpeak, and ST slope are irrelevant and not required to predict the heart disease.

#### Relief

**Fig. 21:**
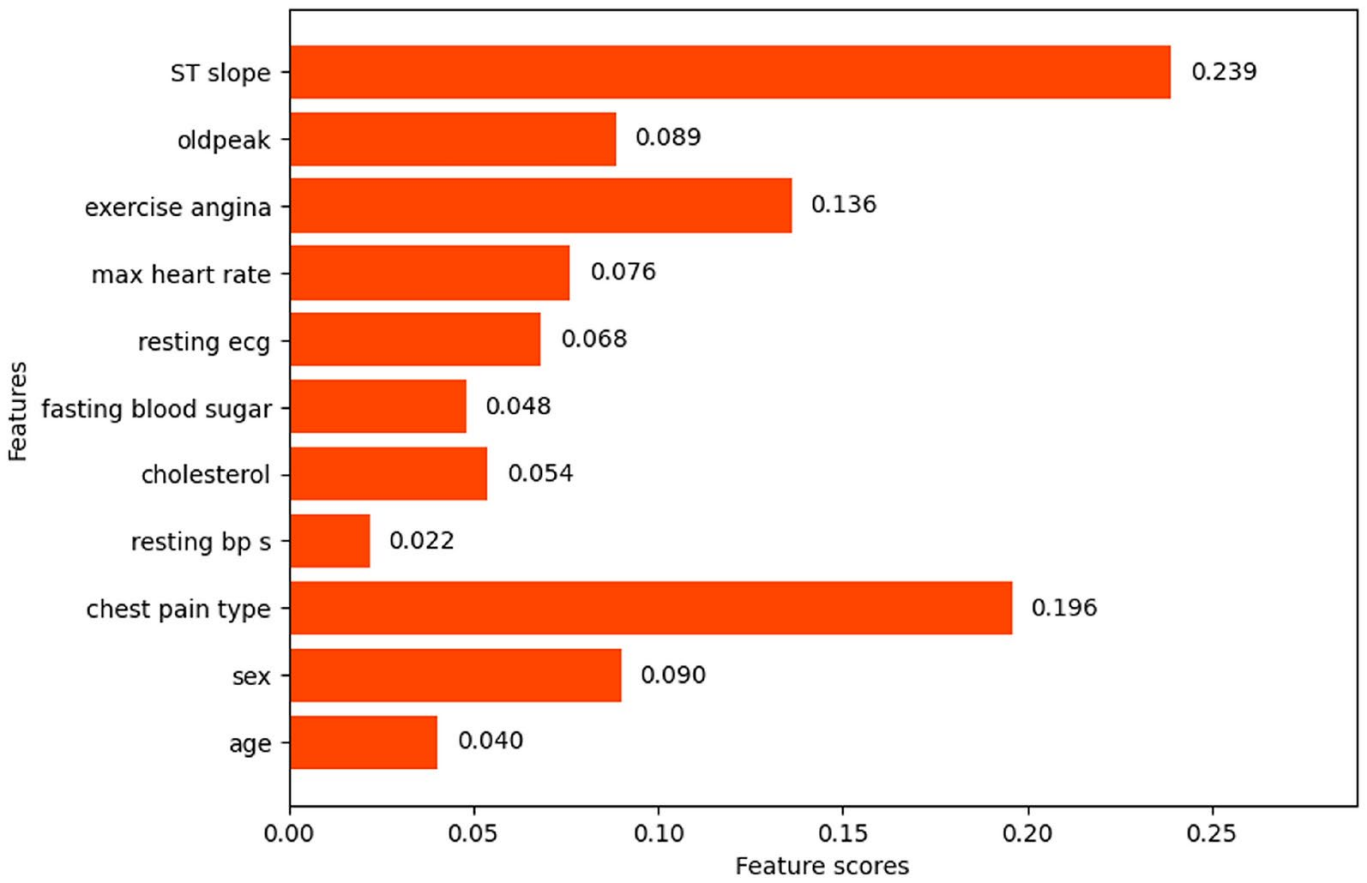
Relief feature scores.

The final output of the Relief feature selection can be observed in Fig. 21 which shows that only three features namely chest pain type, exercise angina, and ST slope have feature scores of more than 0.1. Two feature sets are used for the prediction of heart disease. The first feature set consists of the top 5 ranked features namely ST slope, chest pain type, exercise angina, sex, and oldpeak. The second feature set consists of the top 7 ranked features namely ST slope, chest pain type, exercise angina, sex, oldpeak, max heart rate, and resting ecg.

#### Lasso regularization

**Fig. 22:**
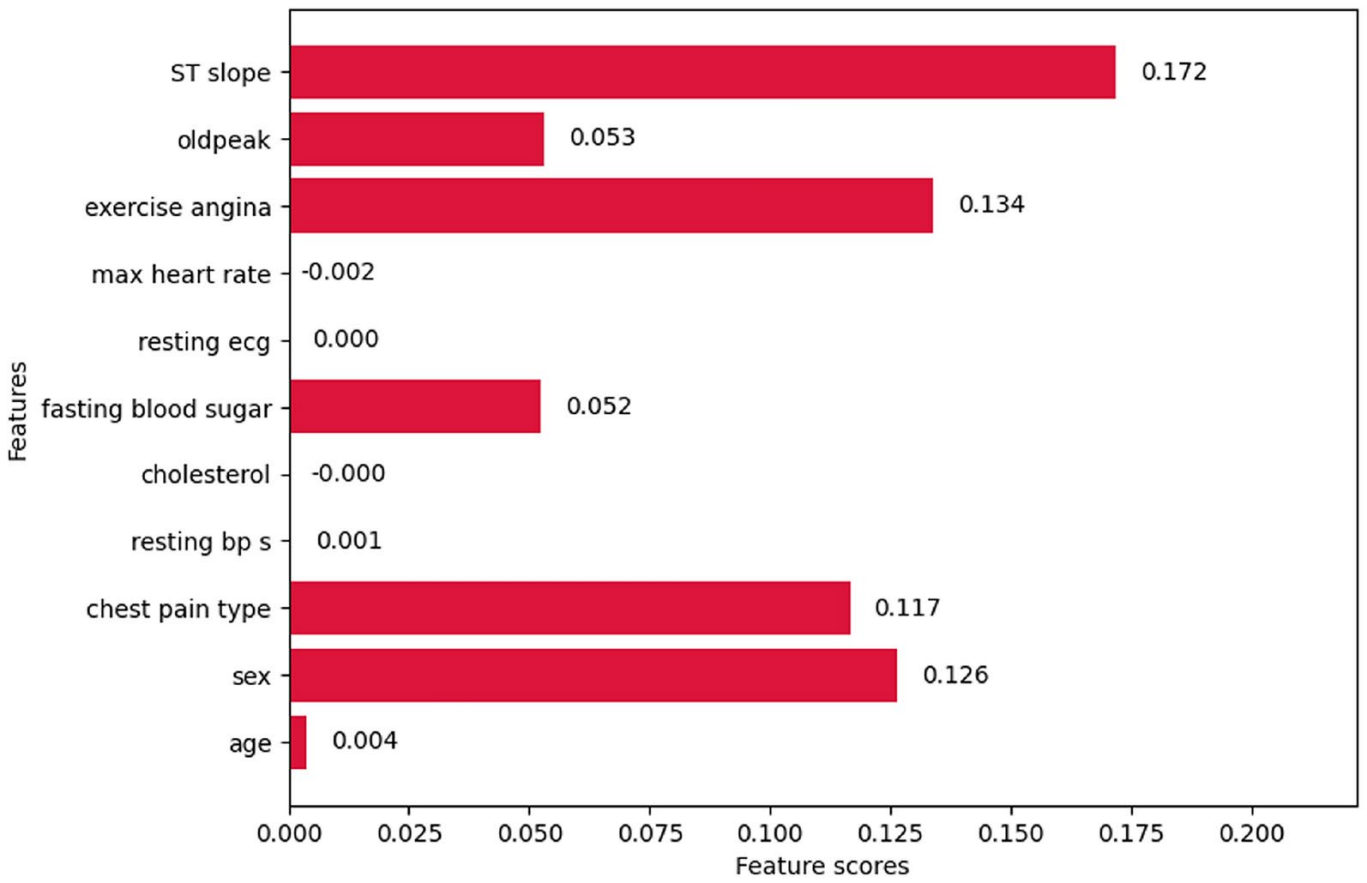
Feature scores of Lasso regularization.

The features such as sex, chest pain type, fasting blood sugar, exercise angina, oldpeak, and ST slope have non-zero coefficients shown in Fig. 22, and these can be used as the final feature set. It can also be observed that some features such as age (0.004), resting bp s (0.001), cholesterol (-0.0004), and max heart rate (-0.002) have non-zero coefficients but, the scores of these features are very near to zero when compared to the feature score of fasting blood sugar which is 0.052. The coefficient of max heart rate is zero, so its feature score is 0.0 and can be eliminated directly.

#### Random forest importance

**Fig. 23:**
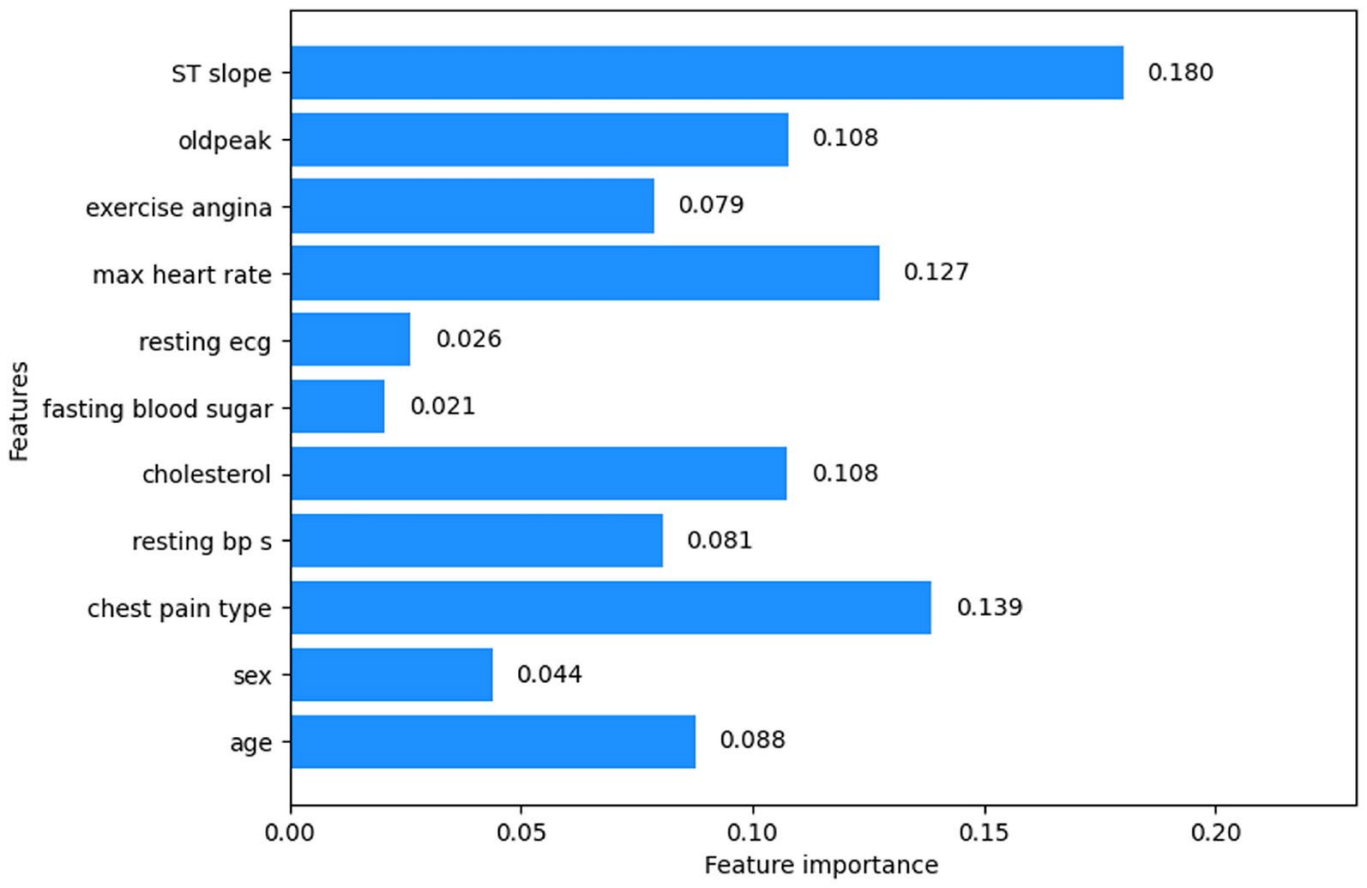
Random forest importance Feature scores.

The feature importance scores and feature ranking by random forest importance feature selection technique are shown in Fig. 23. A threshold of 0.1 was set to the importance scores to find out the efficient features. The features with importance scores lower than 0.1 namely age, resting bp s, exercise angina, sex, resting ecg, and fasting blood sugar will be eliminated. The final feature set consists of the features with an importance score of more than the threshold value namely ST slope, chest pain type, max heart rate, oldpeak, and cholesterol.

#### LDA

**Fig. 24:**
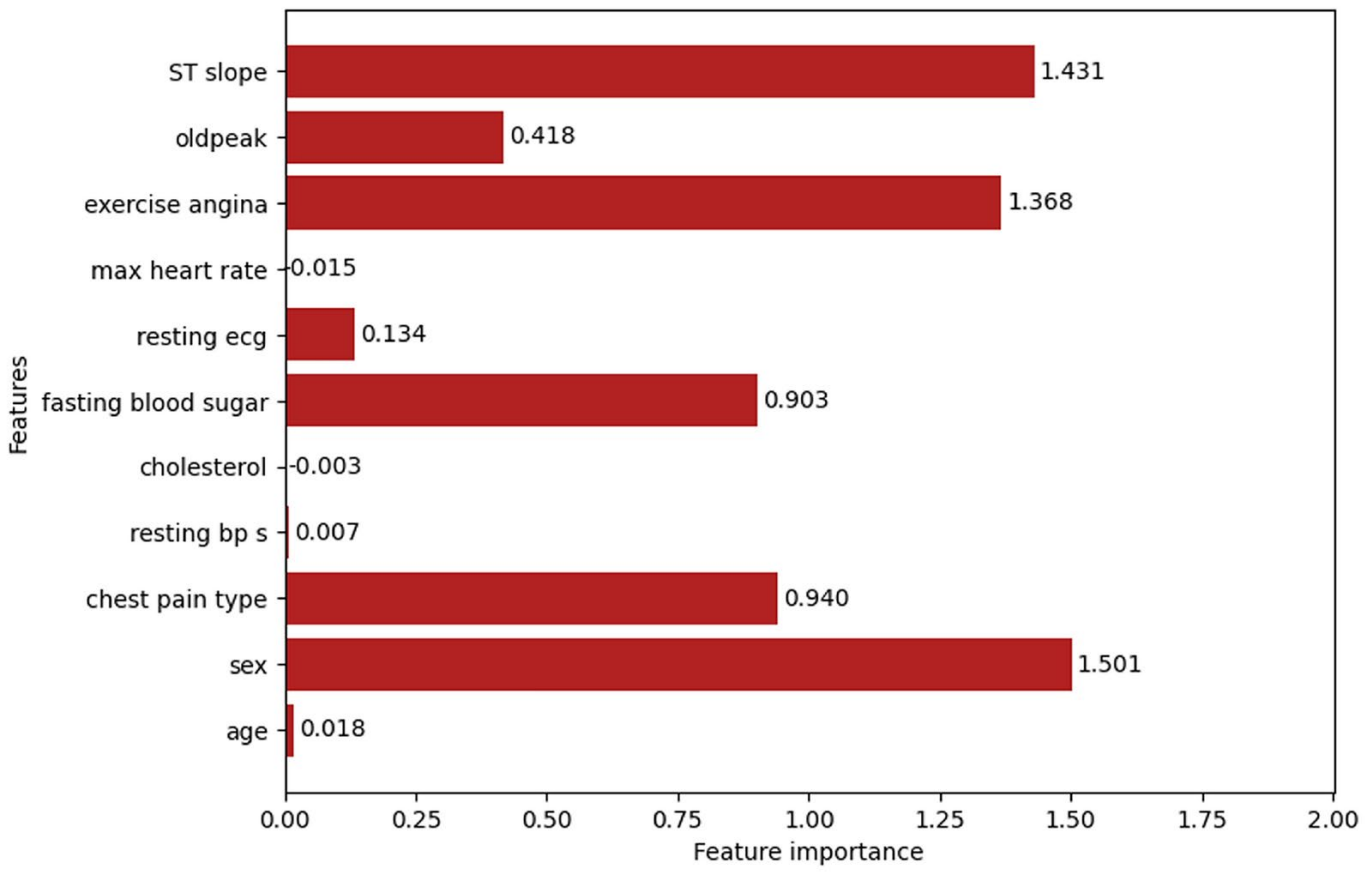
Feature importance.

From Fig. 24, It can be observed that the features such as sex, chest pain type, fasting blood sugar, resting ecg, exercise angina, oldpeak, and ST slope have importance scores more than 0.1, but some features like age, resting bp s, cholesterol, and max heart rate are very close to zero, and some features like cholesterol (-0.003) and max heart rate (-0.015) have negative feature importance scores. So, the final feature set is that shows the scores of the features based on their importance scores. A threshold for the importance scores was set to 0.9 to attain better results. Two feature sets are made accordingly. The first set consists of the top 3 features namely sex, ST slope, and exercise angina. The second set consists of the top 5 features namely sex, ST slope, exercise angina, chest pain type, and fasting blood sugar.

#### PCA

**Fig. 25:**
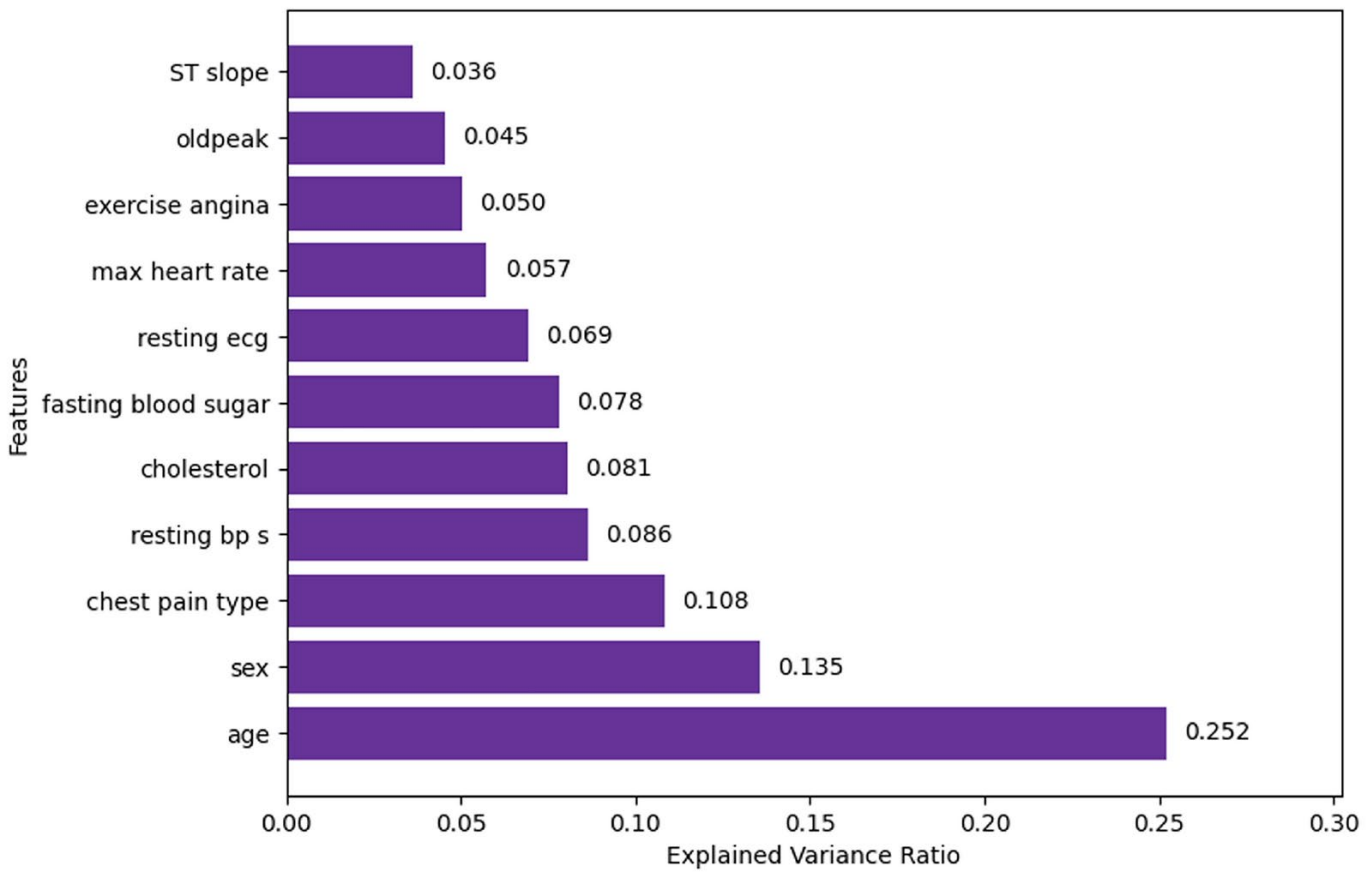
Explained variance ratio.

The explained variance ratio (EVR) of the features is shown in Fig. 25. From this, it is observed that the age has the highest EVR, and the ST slope has the least EVR. The experiment is performed on 2 feature sets. A threshold of 0.1 is chosen for the first feature set. So, it consists of the features with EVR more than 0.1 namely age, sex, and chest pain type. A threshold of 0.08 is chosen for the second feature set. It consists of the features in the first feature set along with resting bp s (0.086) and cholesterol (0.081).

### Results of classifiers without feature selection

This section presents the results obtained by applying the classifiers to the entire dataset without using feature selection techniques. Six metrics are used to evaluate the classifiers. They are accuracy, precision, sensitivity/recall, specificity, F1-score, and area under the curve (AUC). Where the term ‘curve’ in ‘area under the curve’ indicates the receiver operating characteristic (ROC). First, the dataset is divided into training and testing/validation sets in an 80:20 ratio. The training and testing/validation sets consist of 952 and 238 rows of data respectively. Initially, the training was done, then testing, and then hyper-parameter tuning was performed to improve the results of the classifiers.

**Fig. 26:**
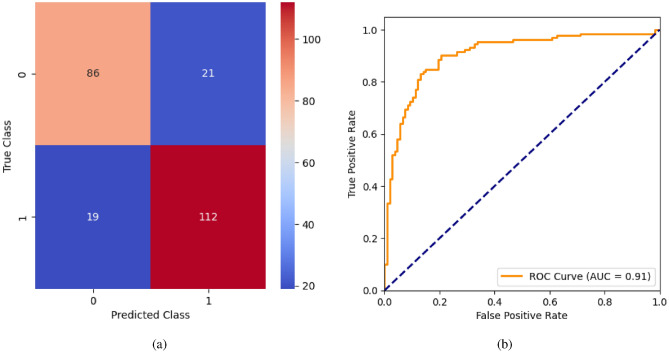
LR without feature selection (**a**) Confusion matrix (**b**) ROC Curve.

**Fig. 27:**
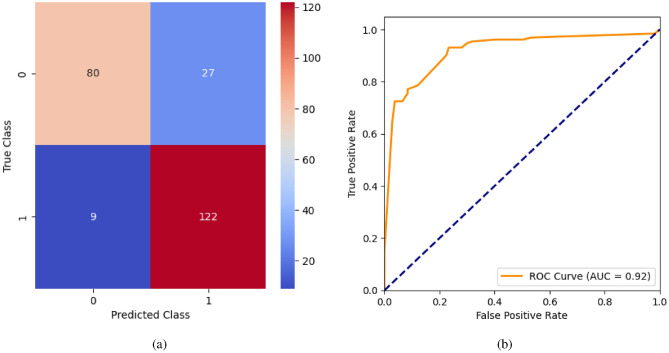
DT without feature selection (**a**) Confusion matrix (**b**) ROC Curve.

**Fig. 28:**
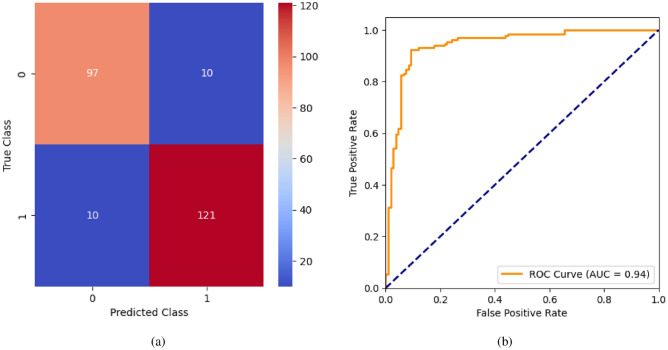
RF without feature selection (**a**) Confusion matrix (**b**) ROC Curve.

**Fig. 29:**
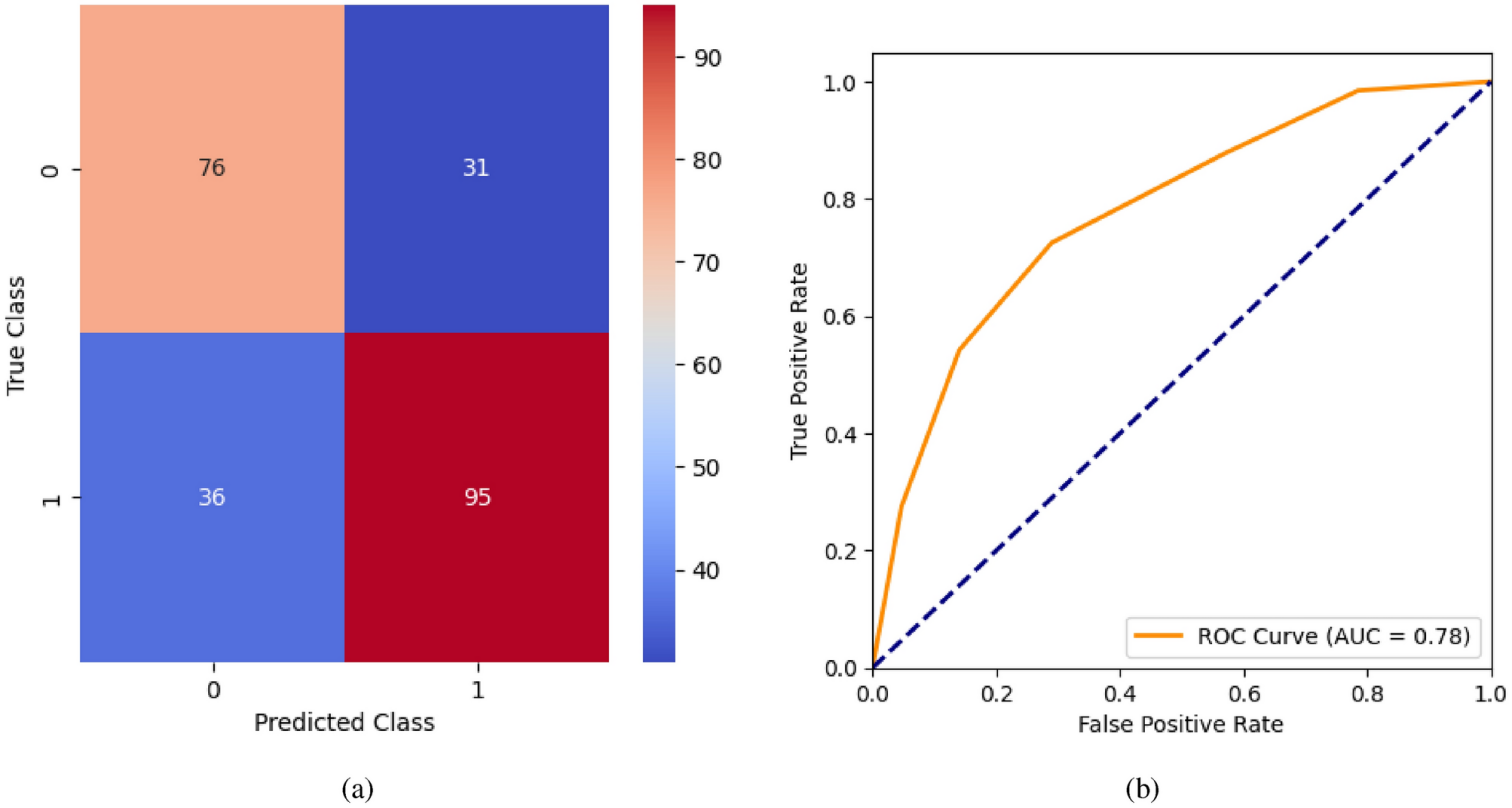
KNN without feature selection (**a**) Confusion matrix (**b**) ROC Curve.

**Fig. 30:**
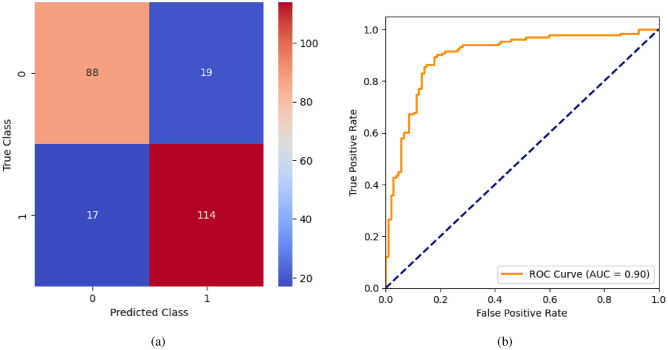
SVM without feature selection (**a**) Confusion matrix (**b**) ROC Curve.

**Fig. 31:**
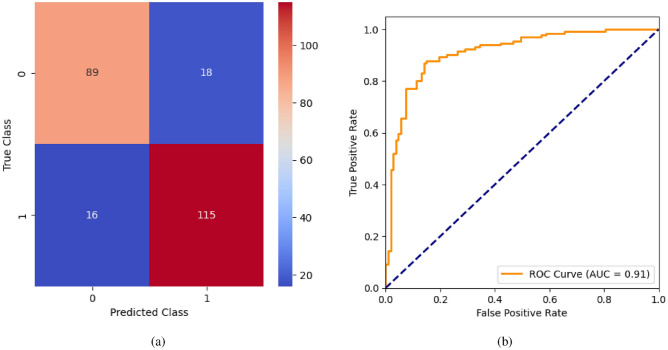
GNB without feature selection (**a**) Confusion matrix (**b**) ROC Curve.

**Fig. 32:**
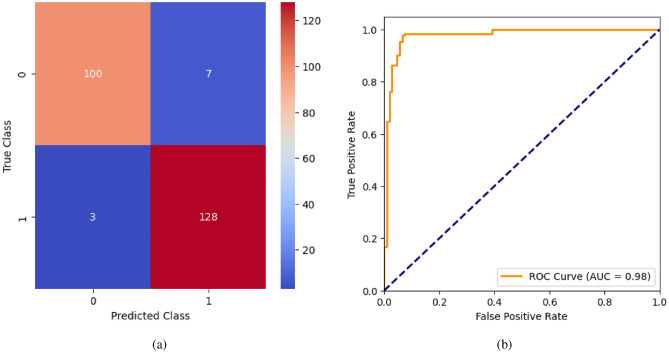
XGBoost without feature selection (**a**) Confusion matrix (**b**) ROC Curve.

When the classifier LR is applied to the dataset, it has achieved 80.99% training accuracy and 83.2% testing accuracy. It can be observed from Fig. 26(a) that the confusion matrix of LR consists of 86 true positives, 21 false negatives, 19 false positives, and 112 true negatives. The precision, sensitivity, specificity, and F1-score achieved by the LR model are 84%, 86%, 80%, and 85% respectively. The ROC curve of the model shown in Fig. 26(b) depicts that the model achieved 91% or 0.91 AUC, which indicates a decent performance. In DT, the hyperparameters such as ‘max_depth’ and ‘random_state’ were tuned to achieve better results. However, there are no major changes in results after the hyperparameter tuning. The DT model has achieved 88.1% training accuracy and 84.9% testing accuracy. It can be observed from Fig. 27(a) that the DT model predicted 80 true positives and 122 true negatives, which is a decent prediction. The precision, sensitivity, specificity, and F1-score achieved by this model are 82%, 93%, 75%, and 87% respectively which depicts that the model is good at identifying actual positives correctly and somewhat lacking in avoiding false positives. DT has scored 92% or 0.92 AUC shown in Fig. 27(b). Initially, the RF model is implemented with a maximum depth of 7 and 100 estimators. Later the maximum depth is changed to 5 with the estimator count the same. However, the RF model has achieved the same results before and after the hyperparameter tuning shown in Fig. 28(a). The RF model achieved 90% training and 91.6% testing accuracies respectively. The RF model achieved decent performance in testing on predicting 97 true positives, 121 true negatives, 10 false positives, and 10 false negatives. The RF model achieved precision, sensitivity, and F1-score of 92% each and a specificity of 91%. Also, the RF model got 94% or 0.94 AUC shown in Fig. 28(b). The KNN classifier is applied to the dataset with 7, 5, and 3 nearest neighbors. Out of those, KNN with 5 nearest neighbors somehow managed to give better results. KNN has achieved 71.9% testing accuracy. From the confusion matrix, as shown in Fig. 29(a), the KNN model predicted 76 positives out of 107 and 95 negatives out of 131, 36 false positives, and 31 false negatives. The precision, sensitivity, specificity, and F1 score achieved by this model are 75%, 73%, 71%, and 74% respectively. It has achieved 78% or 0.78 AUC shown in Fig. 29(b), which indicates that the model’s performance is not good and shouldn’t be taken into consideration. The SVM model has performed better than the KNN model in terms of true positive rate and true negative rate shown in Fig. 30(a). The SVM model achieved 84.9% testing accuracy and 82.8% training accuracy. Predicting 88 true positives out of 107 actual positives and 114 true negatives out of 131 actual negatives is fine, but it would be better if the model had a somewhat better predicting power. The precision, sensitivity, specificity, and F1 score achieved by this model are 86%, 87%, 82%, and 86% respectively. It has also achieved 90% or 0.9 AUC, which is a promising score shown in Fig. 30(b). The performance of GNB or Gaussian Naïve Bayes GNB is shown in Fig. 31. From Fig. 31(a), it can be observed that GNB classified 89 true positives out of 107 actual positives, wrongly classified 16 negatives as positives, 115 true negatives out of 131 actual negatives, and wrongly classified 18 positives as negatives. The training accuracy and testing accuracy achieved by this model are 83.3% and 85.7% respectively. The precision, sensitivity, specificity, and F1 score achieved by this model are 86%, 88%, 83%, and 87% respectively. It has also achieved 91% or 0.91 AUC shown in Fig. 31(b). The XGBoost model has achieved a training accuracy of 100%, testing accuracy of 94.5%, precision of 94%, sensitivity of 96%, specificity of 93%, F1 score of 95%, and 98% or 0.98 AUC score before hyperparameter tuning. However, the results went up very well after hyperparameter tuning. Parameters such as ‘n_estimators’, ‘random_state’, and ‘max_depth’ were changed to 100, 21, and 7 respectively, and parameters such as ‘min_child_weight’, ‘gamma’, ‘subsample’, ‘colsample_bytree’, ‘reg_alpha’, and ‘reg_lambda’ were unchanged. It can be observed from Fig. 32(a) that there are only 3 false positives and 7 false negatives that depict a decent prediction by XGBoost after hyperparameter tuning which showcases the predicting power of the model. After tuning, the XGBoost has achieved a training accuracy of 100%, testing accuracy of 97.3%, precision of 97%, sensitivity, specificity, and F1 score of 98%. It has also achieved 98% or 0.98 AUC shown in Fig. 32(b).

**Fig. 33:**
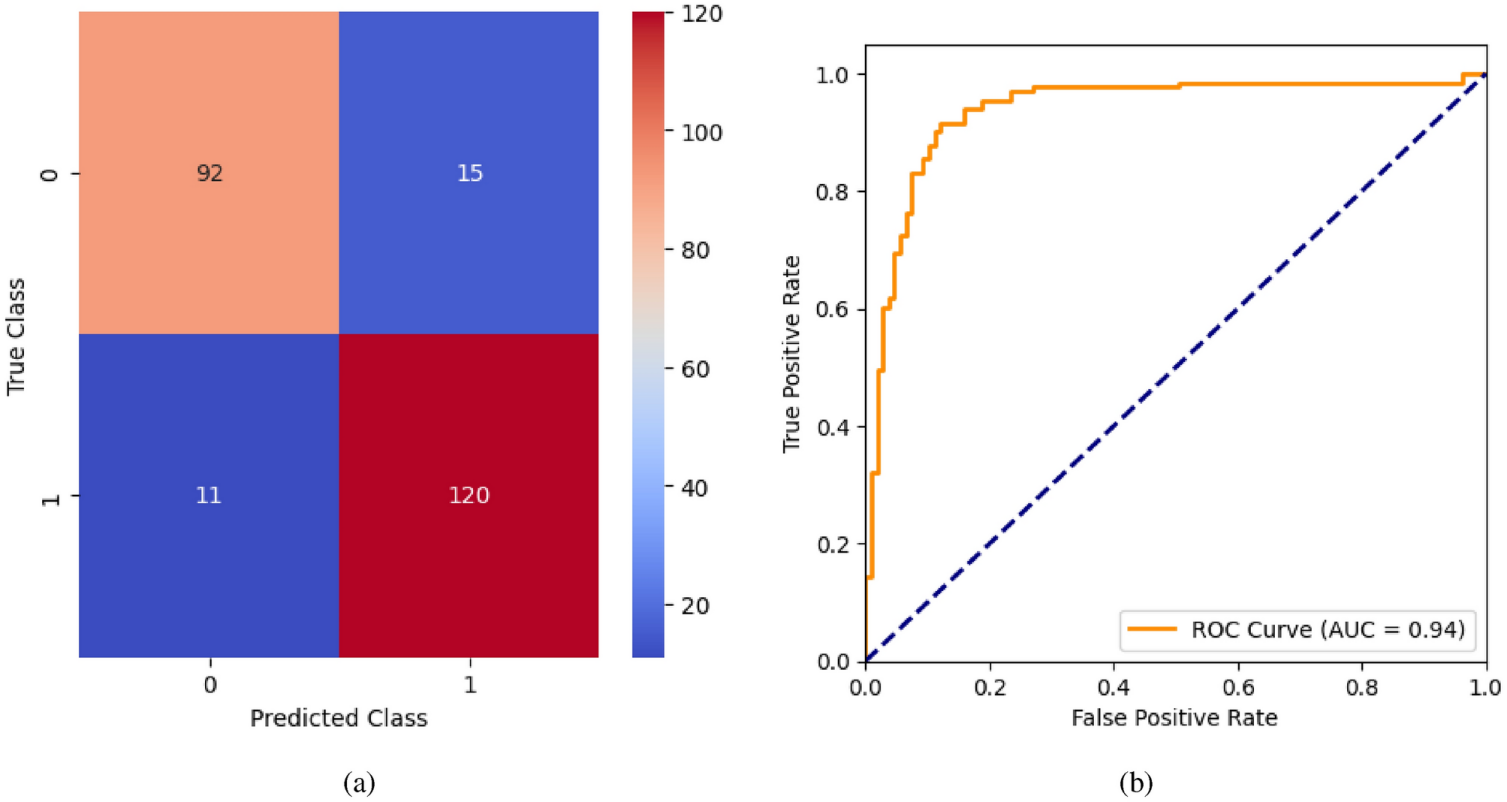
AdaBoost without feature selection (**a**) Confusion matrix (**b**) ROC Curve.

**Fig. 34:**
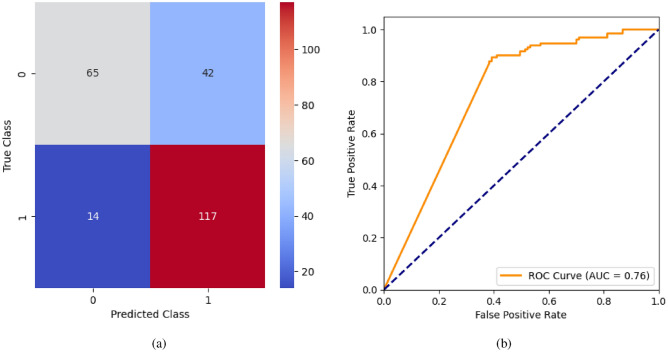
SGD without feature selection (**a**) Confusion matrix (**b**) ROC Curve.

**Fig. 35:**
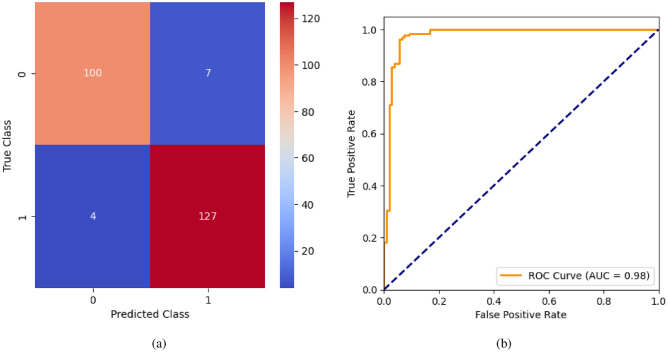
GB without feature selection (**a**) Confusion matrix (**b**) ROC Curve.

**Fig. 36:**
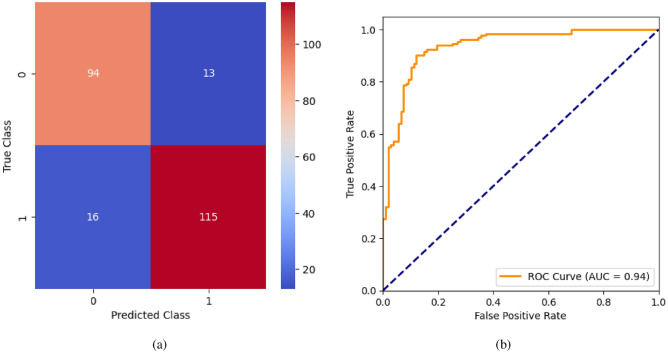
etc without feature selection (**a**) Confusion matrix (**b**) ROC Curve.

**Fig. 37:**
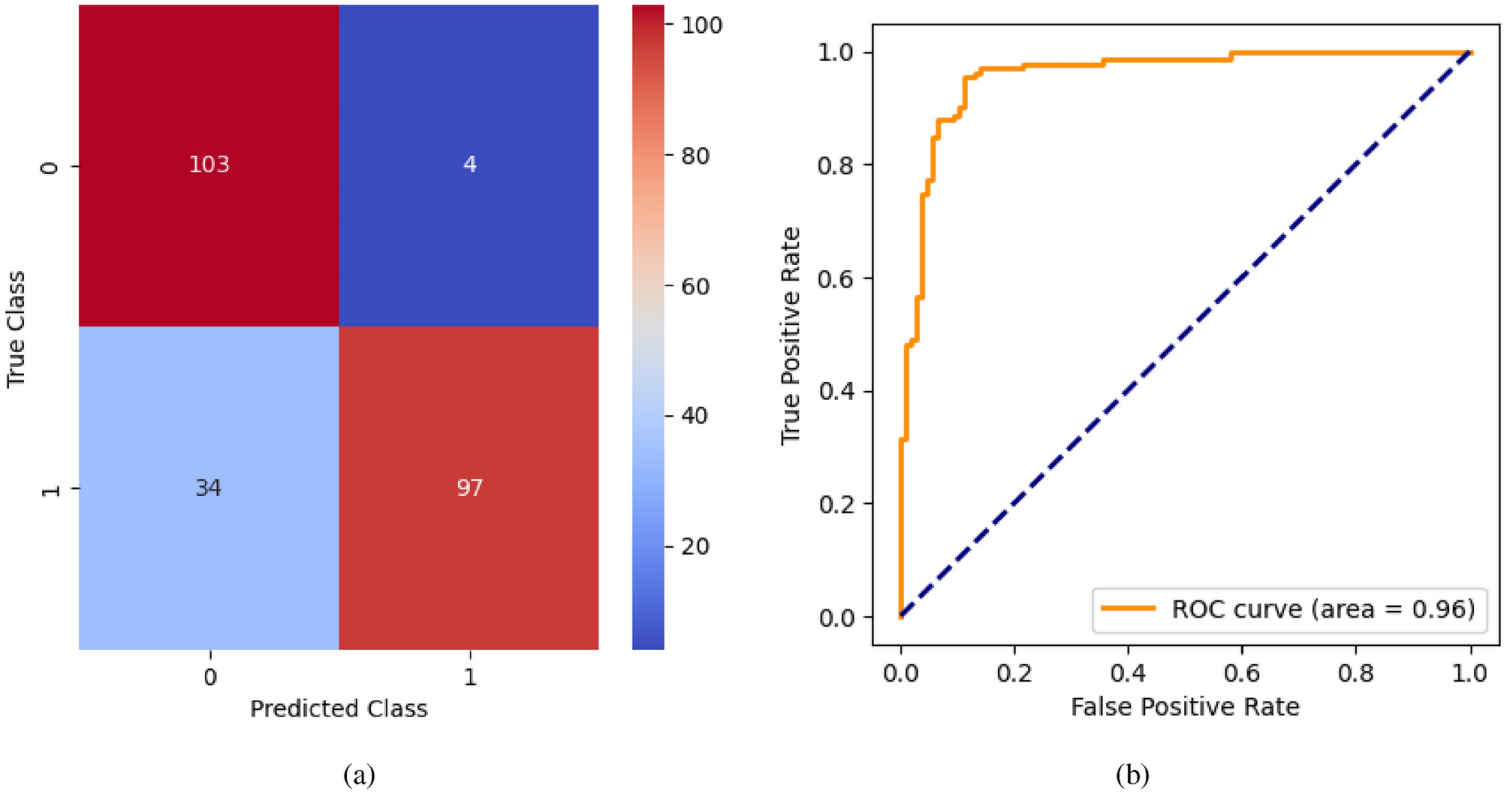
CatBoost without feature selection (**a**) Confusion matrix (**b**) ROC Curve.

**Fig. 38:**
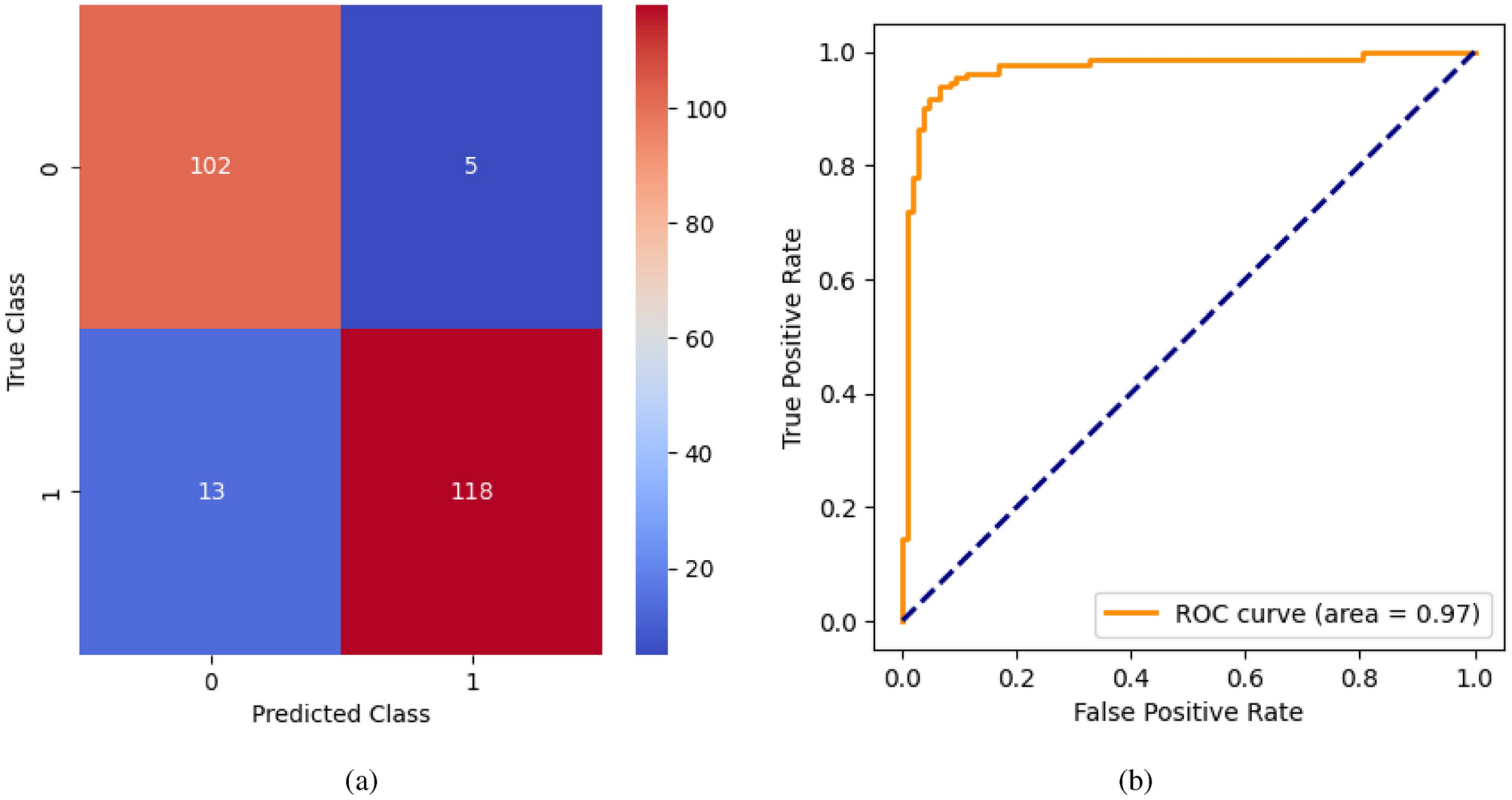
LightGBM without feature selection (**a**) Confusion matrix (**b**) ROC Curve.

**Fig. 39:**
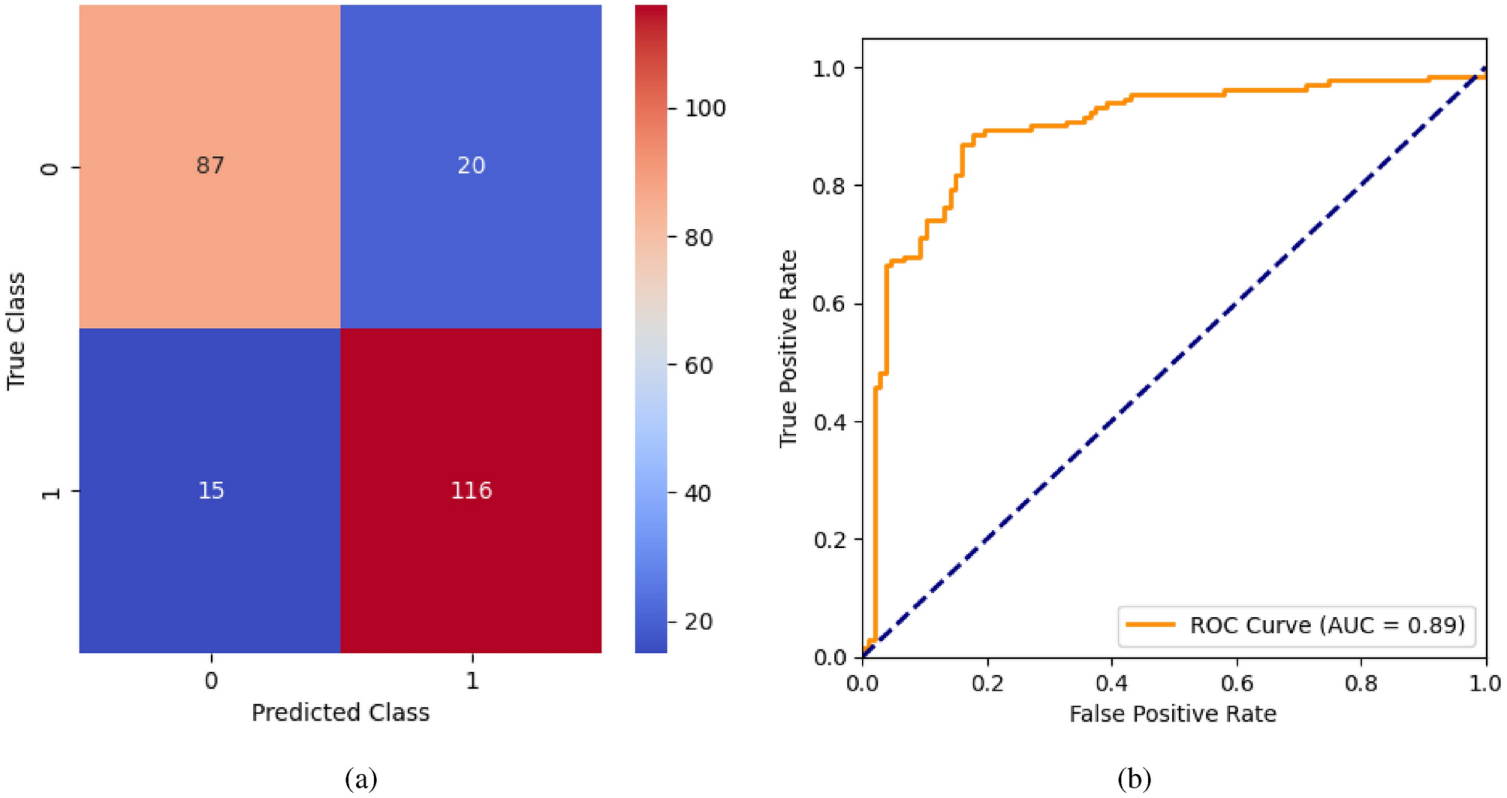
MLP without feature selection (**a**) Confusion matrix (**b**) ROC Curve.

**Fig. 40:**
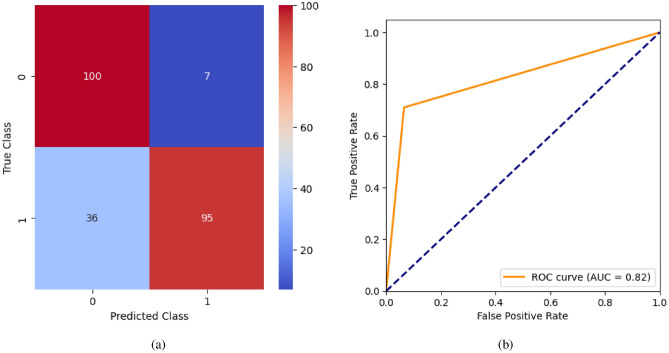
RNN without feature selection (**a**) Confusion matrix (**b**) ROC Curve.

**Fig. 41:**
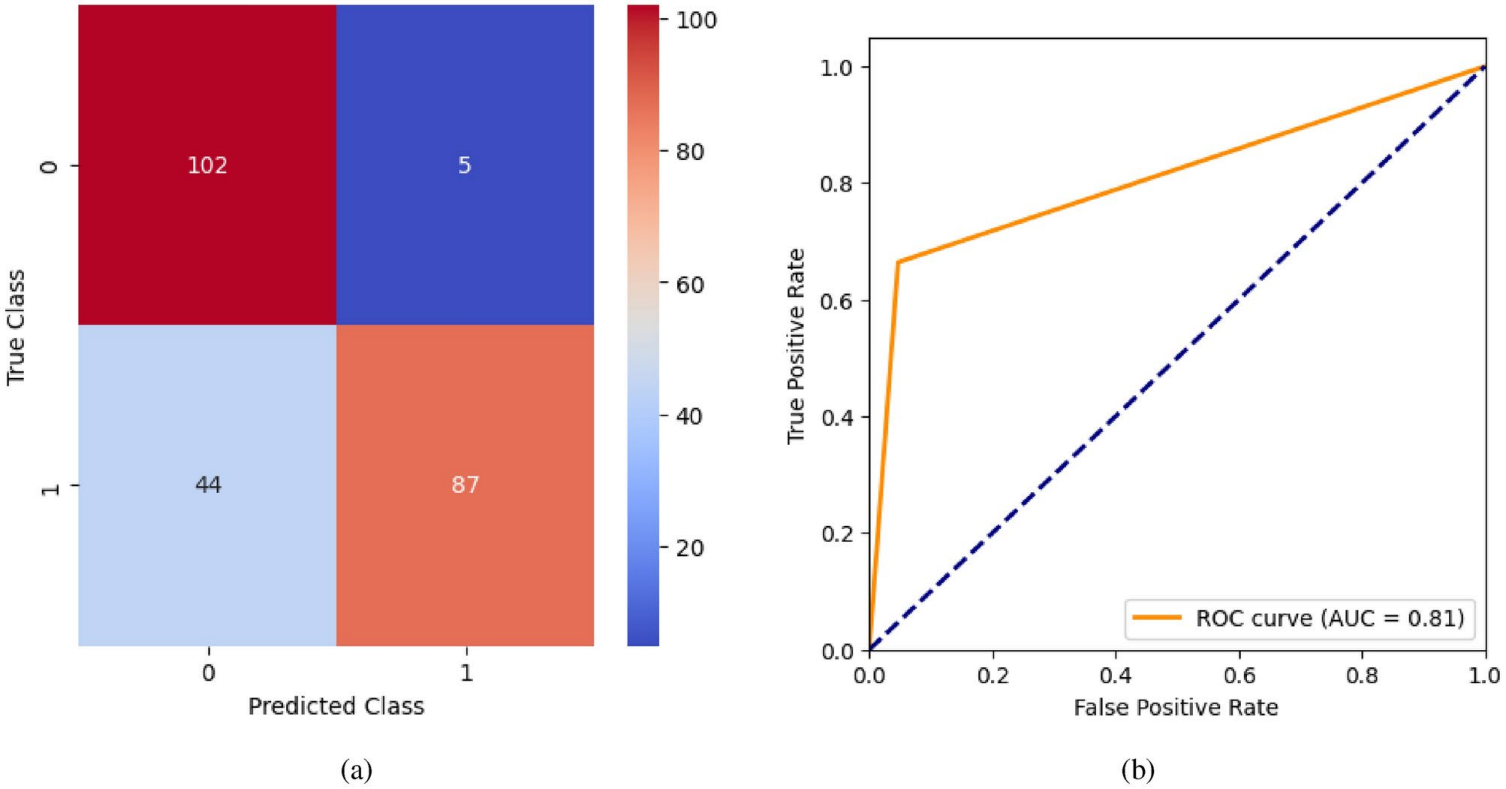
LSTM without feature selection (**a**) Confusion matrix (**b**) ROC Curve.

**Fig. 42:**
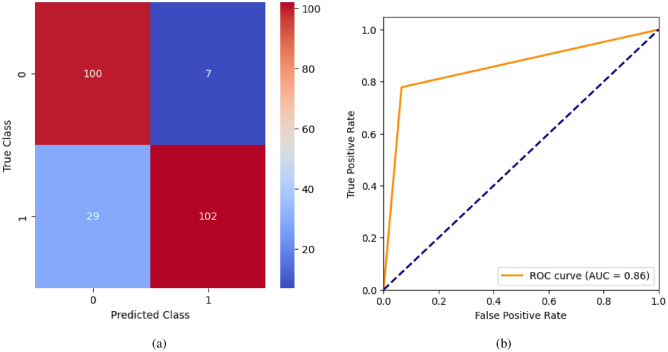
LSTM without feature selection (**a**) Confusion matrix (**b**) ROC Curve.

**Fig. 43:**
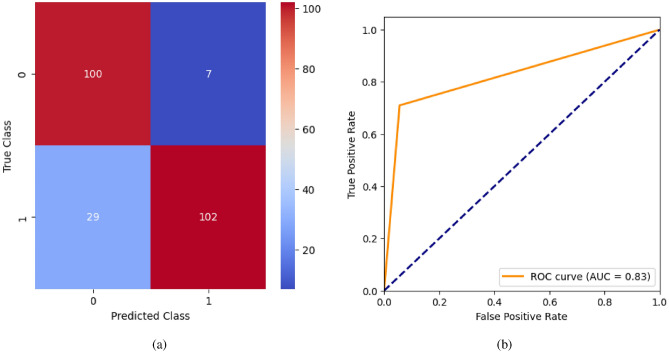
Bi-LSTM without feature selection (**a**) Confusion matrix (**b**) ROC Curve.

**Fig. 44:**
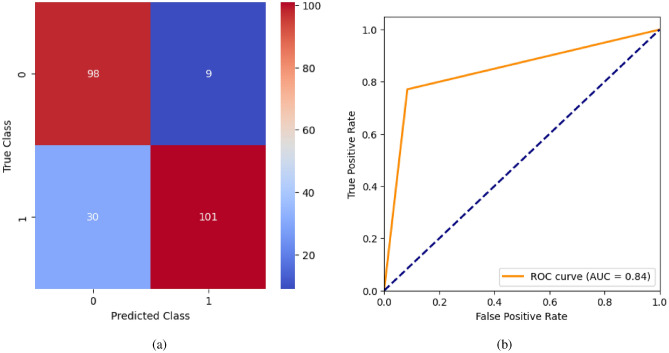
Bi-GRU without feature selection (**a**) Confusion matrix (**b**) ROC Curve.

**Fig. 45:**
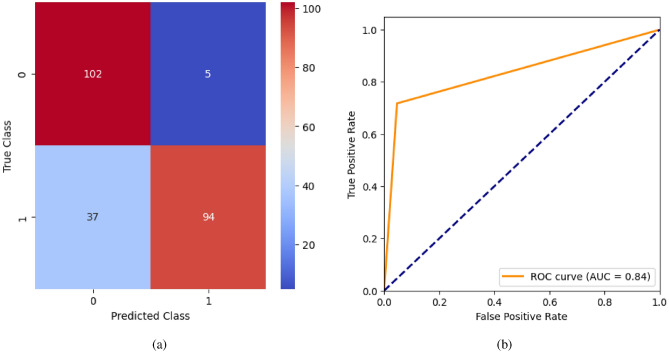
CNN without feature selection (**a**) Confusion matrix (**b**) ROC Curve.

**Fig. 46:**
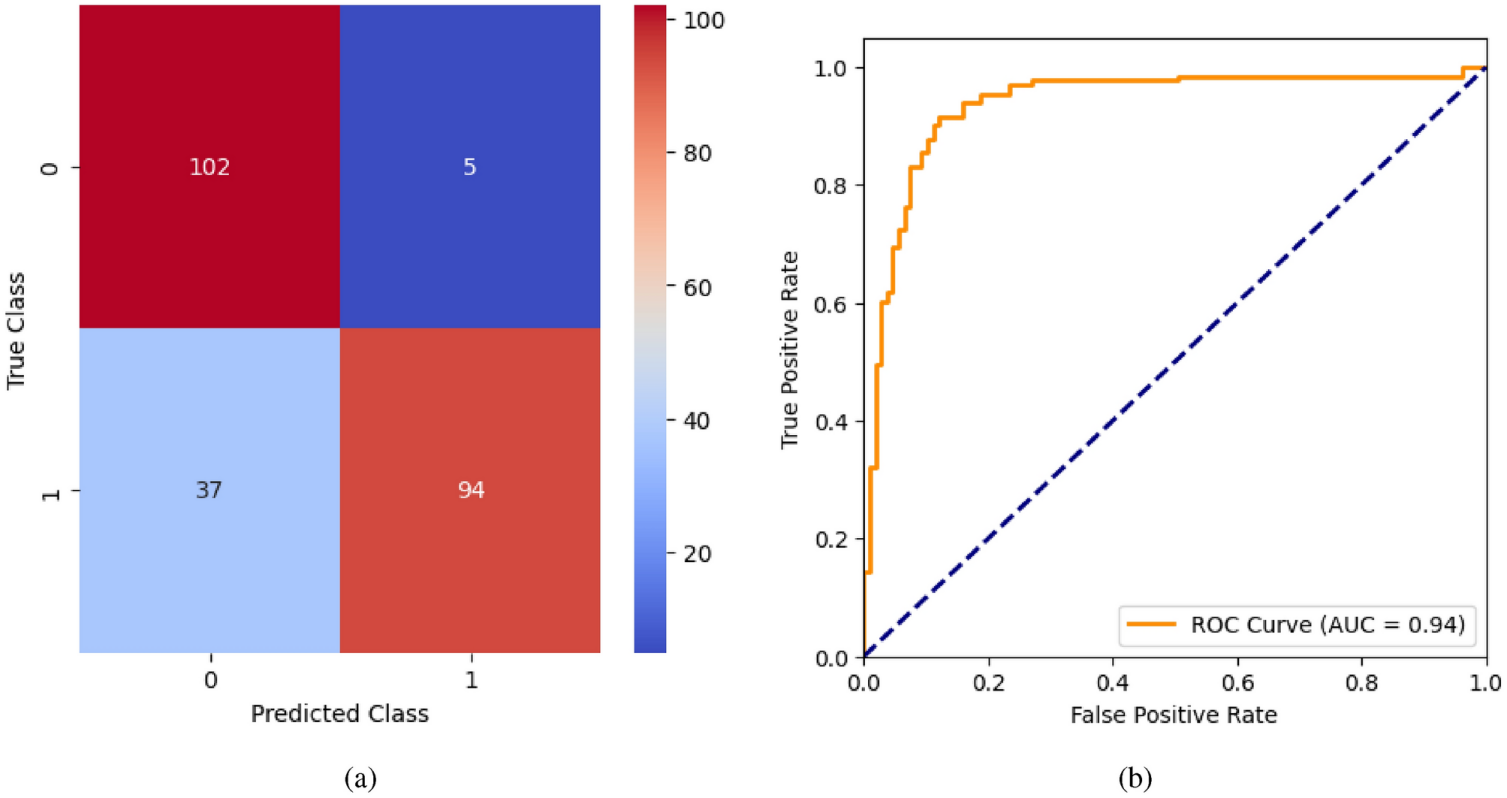
Hybrid model without feature selection (**a**) Confusion matrix (**b**) ROC Curve.

AdaBoost algorithm classified 87 true positives and 120 true negatives out of 107 actual positives and 131 actual negatives respectively, and achieved 87% testing accuracy, 86% precision, 92% sensitivity, 81% specificity, 89% F1 score, and 91% or 0.91 AUC with ‘n_estimators’ of 100. However, after changing ‘n_estimators’ to 50, the AdaBoost model predicted 92 true positives and 120 true negatives which is shown in Fig. 33(a), testing accuracy increased to 89%, and precision, specificity, and F1 score increased by 3%, 5%, 1% respectively. The AUC is increased by 3% i.e., 94% or 0.94 as shown in Fig. 33(b). The SGD model is implemented using the ‘modified_huber’ loss function and ‘random_state’ of 42, which achieved a testing accuracy of 75.2% and predicted 61 true positives and 117 true negatives. However, after the execution of the hyperparameter tuned model using the ‘log’ loss function, regularization strength of 0.001, and a maximum number of iterations of 1000 predicted 65 true positives and 117 true negatives which can be observed from Fig. 34(a). The precision, sensitivity, specificity, and F1 score achieved by this model are 74%, 89% 61% 81% respectively. It has also achieved 76% or 0.76 AUC shown in Fig. 34(b). The hyperparameter tuning of the GB model involved modifying the parameters such as ‘n_estimators’, ‘max_depth’, and ‘random_state’. The execution was done after modifying the ‘n_estimators’ to 50, 100, 150, and 200, and ‘max_depth’ to 3, 5, and 7. The best performance of the GB model is attained with ‘n_estimators’ of 100, ‘max_depth’ of 5, and ‘random_state’ of 42. The GB model predicted 100 true positives and 127 true negatives as shown in Fig. 35(a). The training and testing accuracies achieved by this model are 99.9% and 95.4% respectively. The precision, sensitivity, specificity, and F1 score achieved by this model are 95%, 97%, 94%, and 96% respectively. It has also achieved 98% or 0.98 AUC shown in Fig. 35(b). The performance of the GB model is close to the performance of the hyperparameter-tuned XGBoost model. The hyperparameter tuning of the ETC model is done similarly to the hyperparameter tuning of the GB model. The ‘random_state’ was 42, ‘n_estimators’ was 100, and ‘max_depth’ was 5. The ETC model without applying any feature selection techniques achieved a training accuracy of 87.4% and testing accuracy of 87.8%. The ETC model predicted 94 true positives and falsely predicted 13 actual positives as negatives, 115 true negatives were predicted, and 16 actual negatives were falsely predicted as positives shown in Fig. 36(a). The precision, sensitivity, specificity, and F1 score achieved by this model are 90%, 88%, 89%, and 89% respectively. It has also achieved 94% or 0.94 AUC shown in Fig. 36(b). The hyperparameter tuning is done to the parameters such as ‘iterations’, ‘depth’, ‘learning_rate’, and ‘loss_function’ and the final parameters were 100, 7, 0.1, and ‘Logloss’ respectively. The model predicted most of the positives and 4 actual positives were falsely predicted as negatives as shown in Fig. 37(a). However, 34 actual negatives were falsely predicted as positives, which depicts a lack of predicting power of the model. The model achieved a testing accuracy of 84%. The precision, sensitivity, specificity, and F1 score achieved by this model are 96%, 74%, 96%, and 84% respectively. It has also achieved 96% or 0.96 AUC shown in Fig. 37(b). The LightGBM model has predicted 102 positives and 118 negatives and falsely predicted 5 positives and 13 negatives shown in Fig. 38(a). The parameters used for the prediction were almost like the parameters of the CatBoost model. ‘random_state’ was set to 42, ‘boosting_type’ was ‘gbdt’, and early stopping was set to 10 rounds. ‘gbdt’ is gradient boosting decision tree. The runtime of the LightGBM was relatively very low compared to the other models. However, this model achieved a pretty good testing accuracy of 92.44%. The precision, sensitivity, specificity, and F1 score achieved by this model are 96%, 90%, 95%, and 93% respectively. It has also achieved 97% or 0.97 AUC as shown in Fig. 38(b). The MLP model is built by using 3 hidden layers of 150, 100, and 50 neurons each as previously shown in Fig. 2, and the maximum iterations are set to 1000 and this model is available in Scikit-Learn library. The MLP model has achieved a training accuracy of 84.7% and a testing accuracy of 85.3%. It can be observed from Fig. 39(a) that the predicting power of the MLP model is somewhat good. 87 true positives out of 107 actual positives and 116 true negatives out of 131 actual negatives were predicted and failed to predict 20 positives and 15 negatives. The precision, sensitivity, specificity, and F1 score achieved by this model are 85%, 89%, 81%, and 87% respectively. The MLP model has a decent predicting power as it scored 89% or 0.89 AUC as shown in Fig. 39(b). As previously shown in Fig. 4, the model architecture of RNN depicts that its input layer has 11 nodes which are the features of the dataset that are given as input to the RNN model, 3 simple RNN hidden layers, and a dense output layer with ‘Sigmoid’ activation function which outputs either 0 or 1. The 3 hidden layers consist of 128, 64, and 32 neurons respectively. Adam and binary cross entropy were used as an optimizer and loss function respectively. The number of epochs was set to 100 and patience was set to 10. The RNN model has achieved a testing accuracy of 89.5% and failed to predict 7 positives and 36 negatives as shown in Fig. 40(a). The precision, sensitivity, specificity, and F1 score achieved by this model are 94%, 79%, 94%, and 86% respectively. It has also achieved 82% or 0.82 AUC shown in Fig. 40(b). As previously shown in Fig. 6, the LSTM consists of 1 input layer with 11 nodes, 3 hidden layers of 128, 64, and 32 neurons each, and 1 dense output layer with a sigmoid activation function. Binary cross entropy loss function and Adam optimizer were used for the model compilation. Patience was set to 10 epochs and batch size was 32. After execution, the LSTM model achieved a testing accuracy of 87%. It can be observed from Fig. 41(a) that the LSTM model predicted most of the positives but failed to predict the negatives. The precision, sensitivity, specificity, and F1 score achieved by this model are 95%, 66%, 95%, and 78% respectively. It has also achieved 81% or 0.81 AUC shown in Fig. 41(b). The GRU model was built using an input layer with 11 nodes, 3 hidden layers with 128, 64, and 32 neurons each, and a dense output layer as previously shown in Fig. 8. The GRU model predicted most of the positives and negatives and failed to predict 7 positives and 29 negatives as shown in Fig. 42(a). The model has achieved a testing accuracy of 89.1%. The precision, sensitivity, specificity, and F1 score achieved by this model are 94%, 78%, 94%, and 85% respectively. It has also achieved 86% or 0.86 AUC shown in Fig. 42(b). The model architecture of the Bi-LSTM model as previously shown in Fig. 10 depicts the architecture similar to the architecture of LSTM, but the presence of the Bidirectional() makes the difference. It can be done by importing Bidirectional from tensorflow.keras.layers and is built by using 3 hidden layers with 128, 64, and 32 neurons each. The model has performed well in predicting the positives and achieved a testing accuracy of 87.8%. It has failed to predict 6 positives and 38 negatives as shown in Fig. 43(a). The precision, sensitivity, specificity, and F1 score achieved by this model are 94%, 71%, 94%, and 81% respectively. It has also achieved 83% or 0.83 AUC shown in Fig. 43(b). The Bi-GRU model consists of 3 hidden layers with 128, 64, and 32 neurons each as shown in the model architecture Fig. 12. Patience was set to 10 epochs and the loss function was binary cross entropy. This model has achieved a testing accuracy of 90.3%. The precision, sensitivity, specificity, and F1 score achieved by this model are 92%, 77%, 92%, and 84% respectively. This model performed well in predicting the positives and had a lack of predicting power for negative values as shown in Fig. 44(a). It has also achieved 84% or 0.84 AUC shown in Fig. 44(b). As previously shown in Fig. 13, the CNN model consists of 2 convolutional layers with 128 and 64 neurons each, a dense layer with 64 neurons, and a dense output layer with 1 neuron. Adam optimizer and binary cross entropy loss function were used. Patience was set to 10 epochs and 100 epochs were set for training the CNN model. Out of 107 actual positives and 131 actual negatives, 102 positives, and 94 negatives were predicted correctly and 5 positives and 37 were identified as false positives and false negatives respectively as shown in Fig. 45(a). This model has achieved a testing accuracy of 87.85%. The precision, sensitivity, specificity, and F1 score achieved by this model are 95%, 72%, 95%, and 82% respectively. It has also achieved 84% or 0.84 AUC shown in Fig. 45(b). As previously shown in Fig. 14, the input given to the hybrid model was a concatenated output that came from the layers of the CNN, RNN, LSTM, GRU, Bi-LSTM, and Bi-GRU parallelly. The CNN consists of a convolutional layer with 64 neurons, a max pooling layer with a pool size of 2, and a flatten () layer. The RNN, LSTM, GRU, Bi-LSTM, and Bi-GRU consist of 3 layers with 64, 32, and 16 neurons each respectively. Then, the outputs were concatenated from all branches and an additional dense layer with 128 neurons and a ‘ReLU’ activation function was added before the output layer. Adam optimizer and binary cross entropy loss function were used for the model, and patience was set to 10 epochs. The model has performed well in predicting the positives and somewhat lacking in predicting negatives. It predicted 102 positives out of 107 actual negatives and 94 negatives out of 131 actual negatives as shown in Fig. 46(a). The hybrid model has achieved a testing accuracy of 90.34%. The precision, sensitivity, specificity and F1 score achieved by this model is 92%, 76%, 92%, and 83% respectively. It has also achieved 94% or 0.94 AUC shown in Fig. 46(b).

The summarized results of all the classifiers without feature selection are given in Table 7.

**Table 7:** Results of classifiers (in %) without feature selection.

Classifier	Training accuracy	Testing accuracy	Precision	Sensitivity	Specificity	F1 Score	AUC
LR	80.99	83.2	84	86	80	85	91
DT	88.1	84.9	82	93	75	87	92
RF	90	91.6	92	92	91	92	94
KNN	78.8	71.9	75	73	71	74	78
SVM	82.8	84.9	86	87	82	86	90
GNB	83.3	85.7	86	88	83	87	91
**XGBoost**	**100**	**97.3**	**97**	**98**	**98**	**98**	**98**
AdaBoost	87.8	89	89	92	86	90	94
SGD	75	76.5	74	89	61	81	76
**GB**	**99.9**	**95.4**	**95**	**97**	**94**	**96**	**98**
ETC	87.4	87.8	90	88	89	89	94
CatBoost	86.8	84	96	74	96	84	96
LightGBM	96.5	92.44	96	90	95	93	97
MLP	84.7	85.3	85	89	81	87	89
RNN	89.1	89.5	94	79	94	86	82
LSTM	84.5	87	95	66	95	78	81
GRU	88.7	89.1	94	78	94	85	86
Bi-LSTM	87.8	87.8	94	71	94	81	83
Bi-GRU	88.2	90.3	92	77	92	84	84
CNN	83.2	87.85	95	72	95	82	84
Hybrid Model	89.1	90.34	92	76	92	83	94

Significant values are in [bold].

### Results of classifiers with feature selection

This section discusses the results of classifiers with feature selection and the evaluation and performance of the classifiers.

#### Information gain

The classifiers’ performance in Table 8 is trained based on the features tabulated in Table 4 whose importance scores are greater than 0.1. From Table 8, it is evident that the XGBoost model has given the best results with an accuracy of 94.1%, precision, sensitivity, and F1 score of 95%, specificity of 94%, and AUC of 96%. However, the GB model can also be considered as it has more sensitivity and AUC like the XGBoost model. Tree-based classifiers such as DT, RF, ETC have given almost similar results. LightGBM model outperformed both XGBoost and GB classifiers in AUC, which is 97%. Deep Learning classifiers such as MLP, RNN, LSTM, GRU, Bi-LSTM, Bi-GRU, and Hybrid Model have not performed well enough compared to tree-based classifiers and boosting classifiers.

#### Chi-square test

The classifiers and the respective performance given in Table 9 are trained based on the features selected by strong, medium, and weak thresholds tabulated in Tables 5 and 6 respectively. From Table 9, it is evident that both the XGBoost classifier and the GB classifier have performed well and have almost the same evaluation metrics but GB outperformed XGBoost in AUC, which makes it a considerable model. RF performed well among tree-based classifiers. GB outperformed other boosting classifiers. Bi-LSTM and Bi-GRU performance was almost the same, but sensitivity makes the difference. However, the hybrid model and the CNN has outperformed other deep learning classifiers since it has decent figures in all evaluation metrics. RF, SVM, GNB, XGBoost, SGD, GB, ETC, CatBoost, LightGBM, RNN, GRU, and hybrid model have given their best results with the Chi-square Test using strong threshold features.

#### FDA

The following classifiers are implemented on the 3 feature sets based on Fig. 17. The results of the above classifiers were based on the top 7, top 5, and top 3 features according to their importance scores. LR, KNN, SVM, and GNB achieved those results with the top 5 features. The remaining classifiers’ results were based on the top 7 features. Among the tree-based classifiers, boosting classifiers, and deep learning classifiers, XGBoost and GB have achieved good performance. However, XGBoost dominated GB in some metrics namely accuracy, precision, and specificity. GB outperformed XGBoost in AUC with a small difference. From Table 10, it can be concluded that the XGBoost classifier outperformed the other classifiers.

#### Variance threshold

The following classifiers are implemented on the features that are indicated as ‘True’ in Fig. 18 namely age, chest pain type, resting bp s, cholesterol, resting ecg, max heart rate, and oldpeak. In Table 11, the results of the deep learning classifiers are almost similar. The SGD classifier achieved its best performance by using the modified Huber loss function instead of log loss. As usual, the XGBoost and the GB classifiers outperformed the other classifiers including the tree-based classifiers such as DT, RF, ETC. Boosting algorithms like AdaBoost, CatBoost, and LightGBM could not even compete with XGBoost and GB.

**Table 8:** Results of classifiers (in %) with Information Gain.

Classifier	Training accuracy	Testing accuracy	Precision	Sensitivity	Specificity	F1 score	AUC
LR	81.7	83.2	84	86	79	85	88
DT	87.5	84.5	83	91	77	87	91
RF	88.1	87	86	91	82	88	93
KNN	80	76	77	80	71	71	82
SVM	81.6	83.6	84	87	79	85	88
GNB	83.1	82.8	83	86	79	85	89
**XGBoost**	**97.5**	**94.1**	**95**	**95**	**94**	**95**	**96**
AdaBoost	87	86.6	87	89	84	88	91
SGD	77.1	79	80	82	76	81	79
**GB**	**99.2**	**92.9**	**93**	**95**	**91**	**94**	**96**
ETC	84.7	86.6	88	88	85	88	91
CatBoost	86.8	79.8	96	66	96	78	94
**LightGBM**	**94.6**	**89.5**	**95**	**86**	**94**	**90**	**97**
MLP	82.4	81.1	80	81	72	84	89
RNN	86.1	87	92	61	94	73	70
LSTM	85.3	86.1	91	76	91	83	83
GRU	86.1	85.7	92	72	93	81	82
Bi-LSTM	86.1	84.5	94	69	94	79	82
Bi-GRU	84.5	86.1	93	67	94	78	80
CNN	85.6	85	93	66	94	79	82
Hybrid Model	85.7	84.5	85	68	95	79	92

Significant values are in [bold].

**Table 9:** Results of classifiers (in %) with Chi-Square Test.

Classifier	Training accuracy	Testing accuracy	Precision	Sensitivity	Specificity	F1 score	AUC
LR	82.9	85.7	87	87	84	87	90
DT	85.6	84.5	83	91	78	87	92
RF	87.8	89	89	92	86	90	94
KNN	87.1	82.8	85	83	82	84	89
SVM	83.6	85.7	87	87	84	87	91
GNB	82.4	86.1	88	87	85	87	91
**XGBoost**	**97.6**	**90.3**	**91**	**92**	**89**	**91**	**94**
AdaBoost	85.1	87.4	87	90	84	89	92
SGD	80.3	82.4	85	83	81	84	83
**GB**	**98.3**	**90.3**	**91**	**92**	**89**	**91**	**95**
ETC	85.6	86.9	88	88	86	88	93
CatBoost	86.3	80.7	93	70	94	80	94
LightGBM	93.7	85.3	95	77	95	85	95
MLP	81.1	81.1	86	78	85	82	90
RNN	87	86.1	93	70	94	80	82
LSTM	87	86.9	94	72	94	81	83
GRU	86.1	86.6	93	73	94	82	83
Bi-LSTM	86.1	87.4	93	75	93	83	89
Bi-GRU	87.4	87.4	96	68	96	80	82
CNN	86.7	87.2	94	72	94	81	85
Hybrid Model	86.7	88.7	93	73	93	82	93

Significant values are in [bold].

**Table 10:** Results of classifiers (in %) with FDA.

Classifier	Training accuracy	Testing accuracy	Precision	Sensitivity	Specificity	F1 score	AUC
LR	78.2	82.8	86	82	83	84	87
DT	86.5	83.2	82	89	77	85	91
RF	87.4	86.6	87	89	83	88	93
KNN	81.9	84.9	87	85	85	86	89
SVM	78.8	83.6	85	85	81	85	89
GNB	78	82.4	86	82	83	84	88
**XGBoost**	**98.9**	**89.5**	**91**	**90**	**89**	**90**	**94**
AdaBoost	84.5	84	86	85	83	85	90
SGD	79.3	82.8	86	82	83	84	87
GB	98.3	87.1	90	90	88	90	95
ETC	83.8	84.1	87	86	84	86	92
CatBoost	85.9	81.9	96	70	96	81	94
LightGBM	93.9	81.5	92	73	93	81	94
MLP	80.5	81.1	84	82	80	83	89
RNN	84.8	86.6	94	69	94	79	82
LSTM	84	85.3	93	79	93	85	86
GRU	89.4	85.3	95	74	95	83	85
Bi-LSTM	89.4	85.7	94	76	94	84	85
Bi-GRU	90	85.7	93	73	94	82	83
CNN	86.4	86	94	73	94	82	86
Hybrid Model	80.7	85.3	95	67	95	79	93

Significant values are in [bold].

**Table 11:** Results of classifiers (in %) with Variance Threshold.

Classifier	Training accuracy	Testing accuracy	Precision	Sensitivity	Specificity	F1 score	AUC
LR	78.2	78.2	80	80	76	80	86
DT	84.7	79	81	80	78	80	86
RF	86.6	81.5	83	83	79	83	90
KNN	78.9	71.8	75	73	71	71	74
SVM	78.5	77.3	79	79	75	79	85
GNB	79.7	77.7	81	77	79	79	85
**XGBoost**	**100**	**87.8**	**93**	**85**	**92**	**88**	**94**
AdaBoost	83.6	79.4	84	78	81	81	86
SGD	72	75.6	73	89	80	80	74
**GB**	**99.3**	**87.4**	**89**	**88**	**87**	**89**	**93**
ETC	83.3	81.5	82	85	78	84	88
CatBoost	84.9	81.1	96	69	96	80	92
LightGBM	94.3	79.4	93	68	94	78	91
MLP	72.7	70.6	88	54	91	67	84
RNN	84.5	78.4	92	56	94	70	75
LSTM	77.7	77.3	91	53	94	67	73
GRU	85.7	76.1	91	57	94	70	75
Bi-LSTM	77.7	79	95	53	96	68	74
Bi-GRU	79.4	78.2	92	54	94	68	74
CNN	81	77.7	92	55	94	69	76
Hybrid Model	81	76.9	92	54	94	68	86

Significant values are in [bold].

#### MAD

The following classifiers given in Table 12 are implemented on the features such as namely age, resting bp s, cholesterol, and max heart rate selected by MAD feature selection technique. From Table 12, it can be observed that most of the classifiers have performed worse, mainly SGD and MLP. Many classifiers couldn’t even achieve a minimum of 80% AUC, which shows that those classifiers shouldn’t be considered for real-time prediction of heart disease. Very few classifiers namely XGBoost, GB, CatBoost, and LightGBM that only boosting algorithms could achieve an AUC of more than 80%. Out of these 4 classifiers, XGBoost has decently balanced the evaluation metrics.

#### Dispersion ratio

The following classifiers were implemented on the relevant features namely sex, fasting blood sugar, resting ecg, and exercise angina, whose importance score is more than 1.0, and their performances are given in Table 13. From this table, among tree-based classifiers, RF, ETC, and boosting algorithms, XGBoost and GB have shown a decent performance. However, it can be observed that there is small competition in the performances between XGBoost and GB. The XGBoost achieved above 90% in all evaluation metrics, whereas GB lacked in some metrics.

#### Relief

The results of the classifiers implemented on 2 feature sets consisting of the top 5 and top 7 features based on the importance scores given in Table 14. From this table, it can be observed that both XGBoost and GB performed well among others. Deep learning classifiers have also performed well, but lacked in sensitivity, whereas MLP lacked in specificity. The tree-based classifiers like DT, RF, ETC have shown a decent performance. SGD has also shown a decent performance and balanced all the metrics. The GB classifier achieved a testing accuracy of 89.9% and scored more than the XGBoost in AUC. The XGBoost classifier managed to cross 90% in all evaluation metrics and dominated the remaining classifiers.

#### Lasso

From the results of the classifiers given in Table 15, it can be observed that Bi-LSTM achieved the highest accuracy but lacked in sensitivity and F1 score, but MLP has managed to cross 80% score in all evaluation metrics. Among the tree-based classifiers, RF achieved the highest accuracy but lacked specificity. ETC classifier has managed all the metrics. Among the boosting classifiers, XGBoost outperformed AdaBoost, CatBoost, and LightGBM. Deep learning classifiers like RNN, LSTM, GRU, Bi-LSTM, Bi-GRU, CNN, and Hybrid Model achieved decent accuracy and managed to cross 90% in precision and specificity, but failed to do the same with sensitivity, F1 score, and AUC.

#### RF importance

The performance of the RF importance is given in Table 16. From this, it is observed that among the tree-based classifiers, RF performed better than DT, ETC. Deep learning classifiers have shown decent results in precision and specificity, but couldn’t score well in sensitivity, F1 score, and AUC. The SGD has shown its best performance with the Log Loss function. XGBoost, GB, and LightGBM have shown good results. However, XGBoost outperformed every classifier and managed to score more than 90% in all evaluation metrics.

#### LDA

The results given in Table 17 are based on the feature set whose feature score is more than 0.9. Most of the classifiers except KNN have given their best with the feature set consisting of 5 features, whereas KNN has given its best with the feature set consisting of 3 features. The highest testing accuracy was achieved by XGBoost and Hybrid Model. The XGBoost and GB classifiers managed to achieve a decent score in all evaluation metrics, but the Hybrid Model lacked in sensitivity and F1 score. Tree-based classifiers have also achieved decent performance. Among the boosting algorithms, CatBoost and LightGBM couldn’t show promising results.

#### PCA

From the results given in Table 18, most of the classifiers couldn’t even achieve a testing accuracy of more than 80% except RF, XGBoost, GB, and Bi-LSTM. SGD classifier couldn’t even achieve a minimum of 70% testing accuracy but managed to achieve an AUC of 81%. Deep learning models somehow managed to achieve an accuracy and precision of nearly 80% and 85% respectively but lacked in sensitivity, F1 score, and AUC. Among the tree-based classifiers, RF has shown good results.

**Table 12:** Results of classifiers (in %) with MAD.

Classifier	Training accuracy	Testing accuracy	Precision	Sensitivity	Specificity	F1 score	AUC
LR	69	71	75	71	71	73	75
DT	76.7	71.4	73	76	66	74	76
RF	79.3	76.5	78	80	72	79	83
KNN	78.7	71	75	72	70	73	77
SVM	68.9	69.3	74	69	70	71	75
GNB	70	68.9	76	63	76	69	76
**XGBoost**	**99.8**	**81.9**	**85**	**82**	**82**	**83**	**88**
AdaBoost	75.3	72.7	78	70	76	74	78
SGD	56.7	58	57	98	10	72	54
GB	97.2	77.7	80	80	76	80	86
ETC	74.1	71.8	75	73	71	94	79
CatBoost	76.7	74	92	58	94	71	89
LightGBM	90.8	73.1	89	58	92	70	88
MLP	70.9	68.9	78	60	79	68	78
RNN	75	71	88	34	94	49	64
LSTM	74.1	72.7	86	37	93	52	65
GRU	74.5	72.3	87	31	94	46	63
Bi-LSTM	72.4	71.4	88	35	94	50	65
Bi-GRU	74.6	73.1	90	29	96	44	63
CNN	74.1	71.9	87	34	94	49	67
Hybrid Model	74.1	70.6	85	36	93	51	79

Significant values are in [bold].

**Table 13:** Results of classifiers (in %) with Dispersion Ratio.

Classifier	Training accuracy	Testing accuracy	Precision	Sensitivity	Specificity	F1 score	AUC
LR	80.7	80.3	80	85	75	83	87
DT	87.6	82.4	79	93	63	85	91
RF	88.6	85.3	85	89	80	87	93
KNN	78.8	71.4	75	72	71	73	76
SVM	80.9	81.9	83	85	79	84	87
GNB	81.5	79.8	82	81	79	82	87
**XGBoost**	**100**	**91.2**	**92**	**92**	**91**	**92**	**96**
AdaBoost	86.7	83.6	85	86	81	85	90
SGD	74.7	72.1	84	62	86	71	75
**GB**	**99.1**	**89.9**	**90**	**92**	**87**	**91**	**96**
ETC	86.1	84	84	88	79	86	91
CatBoost	87.4	84.5	96	75	96	84	95
LightGBM	95.3	86.1	95	79	94	86	96
MLP	71	67.6	64	94	33	77	85
RNN	87.4	83.6	92	55	94	69	75
LSTM	86.7	83.2	91	62	93	74	77
GRU	87.4	84	92	63	94	75	78
Bi-LSTM	87.4	81.1	94	66	94	77	80
Bi-GRU	82.4	83.2	94	66	94	77	80
CNN	86.3	85.9	94	69	95	79	82
Hybrid Model	100	100	100	100	100	100	100

Significant values are in [bold].

**Table 14:** Results of classifiers (in %) with Relief.

Classifier	Training accuracy	Testing Accuracy	Precision	Sensitivity	Specificity	F1 score	AUC
LR	82.9	85.3	87	86	84	87	90
DT	85.8	86.6	86	90	82	88	90
RF	86.2	88.2	89	89	87	89	94
KNN	86.9	85.2	84	86	79	85	91
SVM	82.8	84.9	87	86	84	86	90
GNB	81.6	84.5	86	86	82	86	90
**XGBoost**	**98**	**90.3**	**92**	**90**	**91**	**91**	**94**
AdaBoost	84.8	85.7	85	90	80	87	91
SGD	83.1	84.5	87	85	84	86	90
**GB**	**91.1**	**89.9**	**90**	**92**	**88**	**91**	**95**
ETC	84.2	86.1	87	89	83	88	92
CatBoost	85.4	81.9	95	71	95	81	94
LightGBM	91.3	84.9	95	76	95	85	94
MLP	86.9	84.9	83	92	77	87	92
RNN	87.5	87	94	69	94	79	82
LSTM	85.4	85.7	91	71	92	80	81
GRU	86.1	89.1	95	74	95	83	85
Bi-LSTM	85.8	88.2	93	76	93	84	84
Bi-GRU	86.9	88.7	94	74	94	83	84
CNN	86.3	87.8	94	73	94	82	85
Hybrid Model	86.3	88.2	95	73	95	82	92

Significant values are in [bold].

**Table 15:** Results of classifiers (in %) with Lasso.

Classifier	Training accuracy	Testing accuracy	Precision	Sensitivity	Specificity	F1 score	AUC
LR	83.4	85.3	87	86	84	87	91
DT	85.7	86.1	86	90	81	88	92
RF	86.9	88.2	86	94	81	90	94
KNN	87.2	84.5	85	87	81	86	90
SVM	83.6	85.7	87	87	84	87	90
GNB	82.8	86.1	87	88	89	87	91
**XGBoost**	**88**	**87.4**	**88**	**90**	**85**	**89**	**93**
AdaBoost	85.1	84	85	86	81	86	92
SGD 8	3.7	86.1	88	87	85	87	91
GB	91.8	85.3	88	85	86	86	94
ETC	85.2	85.3	87	86	84	87	93
CatBoost	85.9	80.7	96	68	96	80	94
LightGBM	88.3	82.4	92	74	93	82	94
MLP	90.2	83.6	88	82	86	85	91
RNN	87.7	85.3	96	67	96	79	82
LSTM	86.7	88.2	93	70	93	80	81
GRU	87.1	86.6	94	70	94	80	82
Bi-LSTM	86.2	88.7	94	70	94	80	82
Bi-GRU	87.3	88.2	95	66	95	78	80
CNN	87.3	87.2	94	70	94	80	83
Hybrid Model	88.6	86.1	93	70	94	80	93

Significant values are in [bold].

**Table 16:** Results of classifiers (in %) with RF Importance.

Classifier	Training accuracy	Testing accuracy	Precision	Sensitivity	Specificity	F1 score	AUC
LR	80.8	81.1	83	83	79	83	88
DT	87.7	84.5	83	90	78	86	90
RF	87.8	86.1	85	91	80	88	93
KNN	78.9	76.1	77	80	71	79	82
SVM	80.7	81.5	81	86	76	84	87
GNB	81.1	79.8	81	83	76	82	87
**XGBoost**	**99.8**	**92.4**	**94**	**92**	**93**	**93**	**97**
AdaBoost	85.6	84.9	86	86	83	86	91
SGD	73.1	75.6	73	89	59	80	75
GB	98.5	91.6	90	95	87	93	96
ETC	85.7	85.3	86	88	82	87	91
CatBoost	87.1	82.4	94	73	94	82	95
LightGBM	94.6	88.7	96	83	95	89	96
MLP	79.2	76.5	86	69	86	76	86
RNN	88	85.3	89	63	90	74	77
LSTM	87.1	84	90	67	90	77	79
GRU	85.8	85.3	92	65	94	76	79
Bi-LSTM	86.5	82.4	93	68	94	78	81
Bi-GRU	88.4	84	91	66	92	77	79
CNN	87.2	84.3	91	60	92	76	81
Hybrid Model	87.2	84.5	92	59	94	72	91

Significant values are in [bold].

**Table 17:** Results of classifiers (in %) with LDA.

Classifier	Training accuracy	Testing accuracy	Precision	Sensitivity	Specificity	F1 score	AUC
LR	83.7	85.7	88	86	85	87	90
**DT**	**84.9**	**87**	**87**	**90**	**83**	**88**	**92**
**RF**	**85.2**	**87**	**87**	**90**	**83**	**88**	**93**
KNN	83.4	84.9	85	89	80	87	84
SVM	83.1	86.6	87	89	84	88	90
GNB	81.3	83.2	86	82	84	84	89
**XGBoost**	**85.1**	**87.8**	**88**	**91**	**84**	**89**	**92**
**AdaBoost**	**83.7**	**87**	**86**	**91**	**82**	**89**	**92**
SGD	83.5	84.5	86	86	83	86	89
**GB**	**85.5**	**87**	**87**	**90**	**83**	**88**	**92**
ETC	84.7	84.9	88	84	86	86	92
CatBoost	84.9	79	91	69	92	78	92
LightGBM	84.8	79	91	69	92	78	92
MLP	85.7	85.7	86	89	82	87	93
RNN	85	87	90	73	90	80	81
LSTM	85.3	87	93	66	94	77	80
GRU	84.8	87.4	92	64	94	76	79
Bi-LSTM	85.4	85.7	92	64	94	76	79
Bi-GRU	85.9	87.4	94	61	94	74	78
CNN	85.4	87.1	92	67	93	77	82
Hybrid Model	85.3	87.8	89	71	90	79	92

Significant values are in [bold].

**Table 18:** Results of classifiers (in %) with PCA.

Classifier	Training accuracy	Testing Accuracy	Precision	Sensitivity	Specificity	F1 score	AUC
LR	77.4	78.6	79	83	73	81	83
DT	83.2	78.6	72	49	79	80	83
RF	80.7	80.7	81	85	76	83	88
KNN	82.3	74.8	76	79	69	78	80
SVM	79	78.1	78	84	71	81	83
GNB	77.4	77.3	77	83	70	80	83
**XGBoost**	**99.6**	**83.6**	**87**	**82**	**85**	**85**	**91**
AdaBoost	81	76.9	79	79	74	79	86
SGD	70.7	68.9	84	54	87	66	81
**GB**	**96.9**	**84.5**	**86**	**86**	**82**	**86**	**90**
ETC	80.9	79.8	82	81	79	82	87
CatBoost	81.6	75.6	95	59	96	73	92
LightGBM	82.5	76.1	93	61	94	74	92
MLP	76.5	75.6	82	72	80	76	82
RNN	79.6	77.3	86	45	91	59	68
LSTM	82.9	79.8	87	51	91	64	71
GRU	82.4	79.4	85	43	90	58	67
Bi-LSTM	82.8	80.3	85	46	90	60	68
Bi-GRU	79	76.5	87	80	91	63	70
CNN	81.3	78.8	86	52	91	61	72
Hybrid Model	81.3	79.4	87	45	92	59	87

Significant values are in [bold].

#### Discussion and insights

The proposed model is compared with the existing works and the comparison of various metrics is given in Table 19. It is to be noticed that the metrics that were not discussed are represented with ‘_‘ symbol in the table. The proposed XGBoost model demonstrates superior performance across all evaluation metrics, achieving an accuracy of 97.3%, precision of 97%, sensitivity of 98%, specificity of 98%, F1-score of 98%, and AUC of 98%, significantly outperforming state-of-the-art models. For instance, the DNN model tested on the Cleveland dataset^5^ reported an accuracy of 93.33% with a sensitivity of 87.8% and an AUC of 94%, while another DNN model evaluated on multiple datasets^6^ achieved an accuracy of 83.03% with a least execution time of 0.49 seconds but with a sensitivity of 90.9% and specificity of only 69.27%. The HRFLM model^12^ showed an accuracy of 88.4%, a sensitivity of 92.8%, 82.6% specificity, and a 90% F1 score, whereas the RF + FAMD model^14^ achieved slightly higher accuracy at 93.44% with 89.28% sensitivity, a specificity of 96.96%, and 92.5% F1 score. Similarly, the KNN model on the Heart Failure Clinical Records dataset^15^ achieved an accuracy of 90.789% by performing better than Naïve Bayes, Decision Tree, and Random Forest, and the ETC model with SMOTE^26^ achieved an accuracy of 92.62% with precision, sensitivity, and F1 score at 93%. Models like FCMIM + SVM^27^ and GNB^28^ also performed well, with FCMIM + SVM on the Cleveland dataset achieving an accuracy of 92.37%, 89% sensitivity, and 98% specificity and with GNB on the Z-Alizadeh Sani, Statlog, and Cardiovascular disease datasets achieving 95.43%, 93.3%, and 73.2% accuracies, 95.84%, 89.2%, and 69.3% sensitivities, 94.44%, 96.7%, and 77% specificities, and 96.77%, 92.1%, and 71.9% F1 scores. LightGBM achieved a good AUC of 97.8% compared to the results of various methods reported in^29^. However, none of these models match the balanced and superior performance of the proposed XGBoost model, which demonstrates good generalizability and classification robustness on a comprehensive heart disease dataset. Its consistent metrics make it a good candidate for heart disease prediction.

**Table 19:** Performance of proposed model and state-of-the-art models.

Refs.	Dataset	Model	Performance metric (in %)					
			Accuracy	Precision	Sensitivity	Specificity	F1 Score	AUC
^5^	Cleveland	DNN	93.33	–	87.8	91.83	-	94
^6^	Cleveland, Hungarian, Switzerland, Long Beach VA	DNN	83.03	–	90.9	69.27	87.37	–
^12^	Cleveland	HRFLM	88.4	90.1	92.8	82.6	90	-
^14^	Cleveland	RF + FAMD	93.44	–	89.28	96.96	92.59	93.12
^15^	Heart-failure- clinical-records- dataset	KNN	90.78	–	–	–	–	–
^26^	Heart-failure- clinical-records- dataset	ETC	92.62	93	93	–	93	–
^27^	Cleveland	FCMIM + SVM	92.37	–	89	**98**	-	–
^28^	Z-Alizadeh Sani dataset	GNB	95.43	–	95.84	94.44	96.77	–
	Statlog	GNB	93.3	–	89.2	96.7	92.1	–
	Cardiovascular disease dataset	GNB	73.2	–	69.3	77	71.9	–
^29^	CHD dataset	LightGBM	93	96.3	89.7	96.3	92.9	97.8
Proposed	Heart disease Dataset (Comprehensive)	XGBoost	**97.3**	**97**	**98**	**98**	**98**	**98**
		**Superior model**	XGBoost	XGBoost	XGBoost	XGBoost, FCMIM + SVM	XGBoost	XGBoost

Significant values are in [bold].

## Conclusions

In this paper, after conducting a wide range of experiments, the most suitable classification model for heart disease prediction was investigated. Although many models were used to experiment with various feature selection techniques in this research work, the best results were achieved by the XGBoost classifier without feature selection after hyperparameter tuning. The proposed model has achieved test accuracy of 97.3%, precision of 97%, sensitivity, specificity, and F1 score of 98%. Further, the AUC of 98% outperformed the existing models.

### Limitations

A key limitation of the proposed methodology is a requirement of high-end computational facilities when implementing on a large dataset. Thus, this research work considers a limited dataset for the implementation of the proposed methodology. Working with a small-sized dataset may limit the model’s ability to generalize across diverse populations. Therefore, the model’s robustness and generalizability need to be validated using larger, more diverse datasets that better represent real-world scenarios. However, it’s always recommended to apply this methodology on large and diverse datasets to understand its wider applicability. Real-world validation through clinical trials is essential to assess the model’s performance and usability in practical healthcare environments. Additionally, developing strategies to handle noisy data in real-world applications can further enhance the model’s reliability and robustness.

### Practical implications

The proposed model can assist clinicians in early and accurate heart disease prediction, enabling timely interventions and personalized treatment plans. By incorporating such predictive tools into clinical workflows, healthcare professionals can optimize resource allocation, reduce diagnostic errors, and improve patient outcomes. Furthermore, this model can be integrated into hospital information systems to provide real-time decision support, especially in resource-constrained environments where expertise might be limited.

### Future scope

The models performance can be further enhanced with the help of more data. Even though there are a decent number of datasets available on the web, still the amount of data is lacking. Several future directions for dealing with this are given as follows.

- Generative Adversarial Networks (GANs) can be implemented on the dataset to increase its size by generating data that closely resembles the original data.
- IoT devices can be integrated to continuously monitor and extract the relevant data from the patients, which helps increase the quantity of data.
- Future research on Explainable AI (XAI) techniques for heart disease prediction provides an interpretable explanation of the model prediction.
- Integrating federated learning techniques such as differential privacy with heart disease prediction helps in maintaining the confidentiality of the patient‘s data.
- A small language model specifically trained on the data of every existing disease and deploying it as an Android application helps provide suggestions for the patients to prevent the occurrence of the disease.

## Data Availability

The datasets analysed during the current study are available in the [Heart disease dataset (comprehensive)] repository, [https://ieee-dataport.org/open-access/heart-disease-dataset-comprehensive].
